# Interactions among mitochondrial proteins altered in glioblastoma

**DOI:** 10.1007/s11060-014-1430-5

**Published:** 2014-04-13

**Authors:** Ruth F. Deighton, Thierry Le Bihan, Sarah F. Martin, Alice M. J. Gerth, Mailis McCulloch, Julia M. Edgar, Lorraine E. Kerr, Ian R. Whittle, James McCulloch

**Affiliations:** 1 Centre for Integrative Physiology, School of Biomedical Sciences, University of Edinburgh, Hugh Robson Building, Edinburgh, EH8 9XD UK; 2 SynthSys, Synthetic and Systems Biology, University of Edinburgh, Edinburgh, UK; 3 Centre for Cognitive and Neural Systems, University of Edinburgh, Edinburgh, UK; 4 Applied Neurobiology Group, Institute of Infection, Immunity and Inflammation, College of Medical, Veterinary & Life Sciences, University of Glasgow, Glasgow, UK; 5 Department of Clinical Neurosciences, Western General Hospital, Edinburgh, UK

**Keywords:** Glioblastoma, Mitochondria, Clinical proteomics

## Abstract

Mitochondrial dysfunction is putatively central to glioblastoma (GBM) pathophysiology but there has been no systematic analysis in GBM of the proteins which are integral to mitochondrial function. Alterations in proteins in mitochondrial enriched fractions from patients with GBM were defined with label-free liquid chromatography mass spectrometry. 256 mitochondrially-associated proteins were identified in mitochondrial enriched fractions and 117 of these mitochondrial proteins were markedly (fold-change ≥2) and significantly altered in GBM (*p* ≤ 0.05). Proteins associated with oxidative damage (including catalase, superoxide dismutase 2, peroxiredoxin 1 and peroxiredoxin 4) were increased in GBM. Protein–protein interaction analysis highlighted a reduction in multiple proteins coupled to energy metabolism (in particular respiratory chain proteins, including 23 complex-I proteins). Qualitative ultrastructural analysis in GBM with electron microscopy showed a notably higher prevalence of mitochondria with cristolysis in GBM. This study highlights the complex mitochondrial proteomic adjustments which occur in GBM pathophysiology.

**Electronic supplementary material:**

The online version of this article (doi:10.1007/s11060-014-1430-5) contains supplementary material, which is available to authorized users.

## Introduction

Survival times of patients with glioblastoma (GBM; WHO-IV glioma), the most frequent and malignant type of adult brain tumour, remain dismal [1]. Proteomic analyses of glioma have identified numerous altered proteins but have mostly concentrated on whole cell lysates and been dominated by the high abundance proteins [2]. Prospective proteomic studies need to analyse relevant subcellular proteomes and assess protein–protein interactions between altered proteins to gain further insight into glioma pathophysiology and development of targeted therapies.

Mitochondrial dysfunction plays an important role in the pathogenesis of GBM [3–5]. Mitochondria are essential cellular organelles involved in numerous complex physiological processes including energy generation, regulation of cellular proliferation and apoptosis [5]. GBM, like many malignant cancers, favour abnormal energy production via aerobic glycolysis, and show an inherent resistance to apoptosis [6–8]. To provide an insight into mitochondrial dysfunction in GBM, we have quantified the proteomic alterations in mitochondrial fractions from GBM using a label-free proteomics (LC–MS) approach. We highlight the extensive interactions between the altered proteins in relation to oxidative damage and energy metabolism, as well as proteins that are associated with the nuclear transcription factor hepatocyte nuclear factor 4alpha (HNF4A), which plays a pivotal role in gut neoplasia and the targeting of oxidoreductase-related genes [9].

## Materials and methods

Brain samples were obtained from patients undergoing resective brain tumour surgery after informed written consent (ethical approval: LREC/2004/4/16). GBM and control samples were resected from viable tumour tissue and peritumoural brain respectively (patient C1 as exception; see supplementary S1 for details). Peritumoural-control brain was determined using a BrainLAB MRI guided system (merged T1 contrast enhanced plus T2). For proteomics, fresh samples were snap-frozen immediately following resection and stored at −70 °C prior to mitochondrial extraction. Irrespective of the pathology, the global proteomic signature of individual peritumoural-control samples could not be differentiated from the group proteomic signature (supplementary S1). For electron microscopy, samples were placed immediately after surgical excision into fixative (4 %-paraformaldehyde, 5 %-glutaraldehyde, 0.08 M sodium-cacodylate-buffer, pH 7.2).

Mitochondrial-enriched fractions were prepared from GBM (*n* = 6) and peritumoural-control brain (*n* = 6) using the Human Tissue Mitochondria-Isolation Kit (Mitosciences). Immunoblotting with COXI, COXIV and VDAC1 was performed on all samples prior to proteomic analysis to check the integrity of the mitochondrial-enriched fractions.

### Mitochondrial Proteomics of GBM using LC–MS

Protein extracts (100 μg) from mitochondrial fractions were digested and cleaned on SCX column [10]. Capillary–HPLC–MSMS data were acquired using an on-line system consisting of a micro-pump coupled to a hybrid LTQ-Orbitrap XL instrument (using Xcalibur 2.0.7). HPLC–MS methods have been described previously [10–12].

LC–MS runs were analysed using the label-free intensity analysis software Progenesis (NonlinearDynamics, UK). MSMS data were searched using MASCOT Version2.3 against a human plus-contaminant IPI database with 55413 sequences downloaded from www.ebi.ac.uk (v3.42). Variable methionine oxidation, STY phosphorylation, protein N-terminal acetylation and fixed cysteine carbamidomethylation were used in searches.

Progenesis normalises sample intensities and calculates protein intensities from MS peak data as the sum of MS-peak intensities of identified peptides. Protein intensities were used as a relative abundance measure between samples. Within group means were calculated to determine the fold-change and this data was used to calculate the *p*-values using one-way ANOVA. The data were converted using Pride converter v2.5.4 [13] and are available on the public data repository PRIDE (http://www.ebi.ac.uk/pride/; accession numbers 20946-20957). All proteins identified with ≥2 peptides are listed in supplementary S2. Differentially expressed proteins were only considered significant when detected by ≥2 peptides, ≥2-fold-change and *p* ≤ 0.05 for protein intensity change. Hierarchical clustering on the normalized protein intensity was performed using Rheatmap2.

### Bioinformatic analysis of subcellular localisation and protein–protein interactions

All quantified proteins were uploaded to the Database for Annotation, Visualization and Integrated Discovery (DAVID) (http://david.abcc.ncifcrf.gov [14, 15]) to determine subcellular localization based on gene-ontology (GO). Cognizant of the fact that mitochondrial preparations are enriched in, but are not exclusively mitochondrial proteins, significantly altered proteins (*p* ≤ 0.05, ≥2-fold-change) included in the GO term mitochondrion (GO:0005739) were identified from the master list, filtered out, reported and used for network analysis.

Identifiers for altered proteins were uploaded to Ingenuity Pathway Analysis (IPA; http://www.ingenuity.com). Interactomes were algorithmically generated based on direct relationships (physical interactions and/or associations) between eligible proteins. Protein–protein interaction scores are putatively a measure of probability for the interactomes [16].

### Mitochondrial morphology with electron microscopy (EM)

Following a minimum of 20 h in aldehyde fixative, GBM (*n* = 6) and peritumoural-control (*n* = 7) biopsies were dissected into 1–2 mm^3^ pieces suitable for EM processing. Processing, embedding and staining were performed, as described [17]. Evaluation of tissue fixation was carried out by an assessor, blinded to the status of the tissue. Only samples judged to be well fixed under light microscopy were submitted for further analysis.

EM sections were evaluated on a JEOL-CX-100-II transmission electron microscope at 8000× magnification. Images were captured within 3 grid squares selected in a predetermined unbiased pattern.

A multiple squares grid mask was superimposed on the digitised electron micrographs. Mitochondria that lay beneath an intersection were evaluated on the basis of morphology, into one of three classes: normal (with cristae clearly visible over >50 % of the matrix), abnormal (swollen with most of the matrix lost), or uncertain (fewer than normal cristae or condensed) (see supplementary S3). The assessor was blinded to the nature of the tissue samples. A minimum of 119 mitochondria were assessed per tissue sample.

## Results

### Overview of proteomic data from mitochondrial fractions

**Fig. 1:**
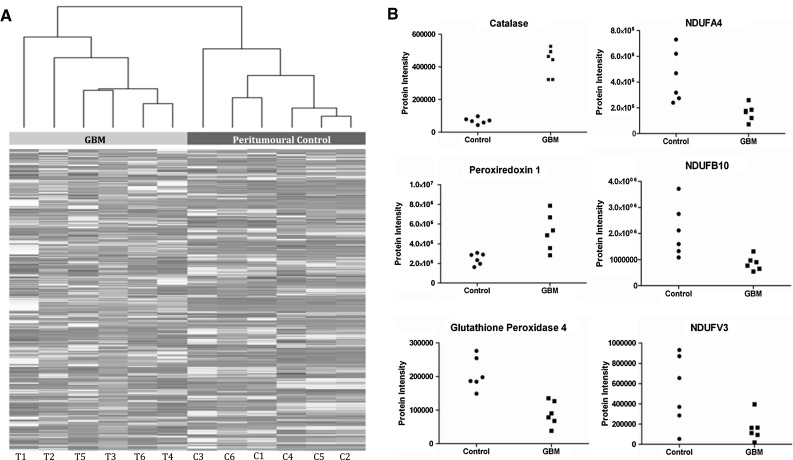
Overview of proteomic data from mitochondrial fractions. Hierarchical clustering of the 902 proteins (normalised protein intensities) detected by LC–MS in mitochondrial fractions of GBM (T) and peritumoural control brain (C). *Each column* (in *greyscale*) represents the proteomic profile (intensities of the 902 proteins) in a single sample. Protein intensities were extracted from Progenesis software. The dendrogram (*x*-axis) provides a visual representation of sample–sample correlations, with correlated samples grouped in branches. Note there are two main branches to this dendrogram which precisely correspond to the two experimental groupings GBM and peritumoural control. The data highlight that there are global differences in the mitochondrial enriched proteome in GBM compared to peritumoural control. Illustrative changes in 3 proteins associated with oxidative damage (catalase, peroxiredoxin 1 and glutathione peroxidase 4) and 3 proteins associated with the Electron Transport Chain (NDUFA4, NDUFB10 and NDUFV3) in GBM relative to peritumoural controls. Each point represents an individual patient

A total of 902 proteins were identified. Hierarchical clustering of the 902 normalized protein intensities revealed two major clusters corresponding to GBM and peritumoural-control cohorts (Fig. 1a). Western-blotting using markers for outer (VDAC1) and inner (COX1, COXIV) mitochondrial membranes indicated that all three proteins were present in the mitochondrial-enriched fraction and absent in the post-mitochondrial supernatant fraction from GBM and peritumoural-control samples (supplementary S4). These data indicate that there has been minimal breakdown of mitochondrial membranes during the fractionation procedure, and that mitochondrial fractionation or integrity is not different in GBM compared to peritumoural-control brain.

### Alterations in the mitochondrial proteome of GBM

The 902 proteins identified by mass spectrometry were categorised using DAVID and 256 proteins were classified as mitochondrially associated based on the GO designation *mitochondrion*. This fraction of mitochondrial (28 %) versus other proteins (72 %), is consistent with published studies using similar preparations [10, 18].

117 of these mitochondrial proteins were significantly (*p* ≤ 0.05, ≥2-fold change) altered between the GBM and peritumoural-control groups: 39 proteins increased and 78 proteins decreased in GBM. All proteins are listed with names and statistical parameters in supplementary S2, while the numbers of proteins in each category are summarised in supplementary S5. The top 40 altered proteins (ranked by *p* value) are listed in Table 1 and illustrative changes presented (Fig. 1b). Some proteins (for example, CAT, PRDX1, GPX4) display a small variation across the different samples, and others (for example NDUFA4, NDUFB10, NDUFV3; all Electron Transport Chain (ETC) complex I proteins) display a broader variation, particularly in peritumoural-control samples.

**Table 1:** Mitochondrial proteins altered in GBM

Protein ID	Gene name	Protein name	*p*-value^a^	Fold change^b^	# pep^c^	Score^d^
INCREASED in GBM						
IPI00465436	CAT	Catalase	1.45E–07	6.1	3	97
IPI00002520	SHMT2	Serine hydroxymethyltransferase	3.48E–06	5.5	2	82
IPI00419237	LAP3	Isoform1 of aminopeptidase	0.0001	5.1	7	347
IPI00026105	SCP2	Isoform SCPx Non-specific lipid-transfer protein	0.0002	5.5	2	62
IPI00215901	AK2	Adenylate kinase 2	0.0002	5.3	4	223
IPI00017726	HSD17B10	Isoform 1 3-hydroxyacyl-CoA dehydrogenase 2	0.0003	3.4	7	538
IPI00032103	GATM	Glycine amidinotransferase	0.0006	6.0	9	595
IPI00096066	SUCLG2	Succinyl-CoA ligase [GDP-forming] subunit β	0.0008	10	4	167
IPI00033217	AASS	Alpha-aminoadipic semialdehyde synthase	0.0012	4.5	4	184
IPI00019906	BSG	Isoform 2 of Basigin	0.0014	2.1	3	137
IPI00026958	FDXR	NADPH:adrenodoxin oxidoreductase	0.0015	3.2	3	107
IPI00306748	ABCB7	ATP-binding cassette sub-family B member	0.0016	2.2	2	133
IPI00011201	ME2	NAD-dependent malic enzyme	0.0023	2.2	4	309
IPI00218342	MTHFD1	C-1-tetrahydrofolate synthase	0.0024	8.3	2	102
IPI00291262	CLU	Isoform1 of Clusterin	0.0027	5.7	8	545
IPI00022314	SOD2	Superoxide dismutase [Mn]	0.0029	4.5	16	1,408
IPI00910602	NEFH	Isoform1 of Neurofilament heavy polypeptide	0.0032	3.5	9	377
IPI00000874	PRDX1	Peroxiredoxin-1	0.0034	2.1	13	664
IPI00003482	DECR1	2,4-Dienoyl-CoA reductas	0.0045	2.4	9	497
IPI00001960	CLIC4	Chloride intracellular channel protein 4	0.0048	6.3	10	500
IPI00005040	ACADM	Medium-chain specific acyl-CoA dehydrogenase	0.0049	4.5	6	219
IPI00011937	PRDX4	Peroxiredoxin-4	0.0052	2.9	5	279
DECREASED in GBM						
IPI00032904	SNCB	Beta-synuclein	8.81E–05	0.2	4	355
IPI00333763	GLRX5	Glutaredoxin-related protein 5	0.0005	0.3	2	64
IPI00018246	HK1	Hexokinase-1	0.0014	0.4	48	3,168
IPI00304814	GPX4	Phospholipid hydroperoxide glutathione peroxidase	0.0016	0.4	3	84
IPI00026516	OXCT1	Succinyl-CoA:3-ketoacid-coA transferase 1	0.0018	0.4	21	1,684
IPI00658109	CKMT1B	Creatine kinase, ubiquitous	0.0019	0.3	23	1,698
IPI00017802	AUH	Methylglutaconyl-CoA hydratase	0.0021	0.1	2	50
IPI00217232	SUCLA2	Isoform2 succinyl-CoA ligase [ADP-forming] β	0.0025	0.3	9	357
IPI00010415	ACOT7	Isoform1 cytosolic acyl co-A thioester hydrolase	0.0026	0.4	3	113
IPI00003970	ME3	NADP-dependent malic enzyme	0.0030	0.3	9	466
IPI00011770	NDUFA4	NADH dehydrogenase [ubiquinone]1α, subunit 4	0.0035	0.4	3	78
IPI00289159	GLS	Isoform KGA of Glutaminase kidney isoform	0.0038	0.3	22	1,484
IPI00216085	COX6B1	Cytochrome c oxidase subunit 6B1	0.0039	0.3	7	434
IPI00386271	SLC25A12	Calcium-binding mitochondrial carrier Aralar1	0.0039	0.3	22	1,460
IPI00020510	CISD1	CDGSH iron sulfur domain-containing protein 1	0.0041	0.3	6	272
IPI00479905	NDUFB10	NADH dehydrogenase [ubiquinone]1β, 10	0.0042	0.4	6	303
IPI00029558	NDUFC2	NADH dehydrogenase [ubiquinone] 1, C2	0.0048	0.3	2	45
IPI00003856	ATP6V1E1	V-type proton ATPase subunit E 1	0.0049	0.4	5	278

The 40 most significantly different mitochondrial proteins in GBM relative to peritumoural controls (*p* ≤ 0.05, ≥2-fold change, ranked by *p*-value). For a list of all 117 significantly altered mitochondrial proteins (*p* ≤ 0.05, ≥2-fold change) see supplementary information S2. S2 also lists non-mitochondrial proteins significantly altered and all unaltered proteins identified with ≥2 peptides

The protein accession number (IPI), gene name, and protein name are listed for each altered protein, together with: ^a^ *p*-value evaluated by one-way ANOVA on intensity data (computed using Progenesis)

^b^ Ratio of the average protein intensity in GBM and control samples, measured by Progenesis

^c^ Number of peptides used for quantitation

^d^ Protein identification score (calculated by Mascot)

### Functional analysis of differentially expressed mitochondrial proteins

Functional categorization of the 117 mitochondrial proteins altered in GBM revealed two prominent functional groups associated with antioxidants and energy metabolism.

#### Antioxidants

A general increase in several proteins involved in antioxidant defence (including CAT, PRDX1, PRDX4 and SOD2) were observed in GBM, with the exception of GPX4, which was significantly decreased in GBM.

#### Energy metabolism (ETC, TCA cycle, lipid and amino acid metabolism)

Reductions in the levels of numerous proteins involved in energy metabolism were observed in GBM. Multiple components of the ETC were decreased in GBM (~40 proteins; including NDUFA4, NDUFB10, NDUFC2 and COX6B1). Of these ETC proteins, 23 proteins were components of ETC Complex-I. In contrast a number of proteins involved in lipid metabolism (for example, ACADM, DECR1 and SCP2) and amino acid synthesis and metabolism (AASS, LAP3, MTHFD1L and SHMT2) were increased in GBM. Also several proteins integral to energy production prior to the ETC were dysregulated in GBM: AK2, GATM, ME2 and SUCLG2 were increased; and CKMT1B, GLS, HK1, ME3 and SUCLA2 were decreased.

#### Other proteins of interest

Several proteins, for example BSG (increased in GBM), SNCB (decreased in GBM) and IDH3 (with IDH3A, IDH3G and IDH3B all decreased in GBM), did not fall into an obvious functional grouping but are pertinent to tumour pathophysiology (see “Discussion” section).

### Interactomes of mitochondrial proteins altered in GBM

**Fig. 2:**
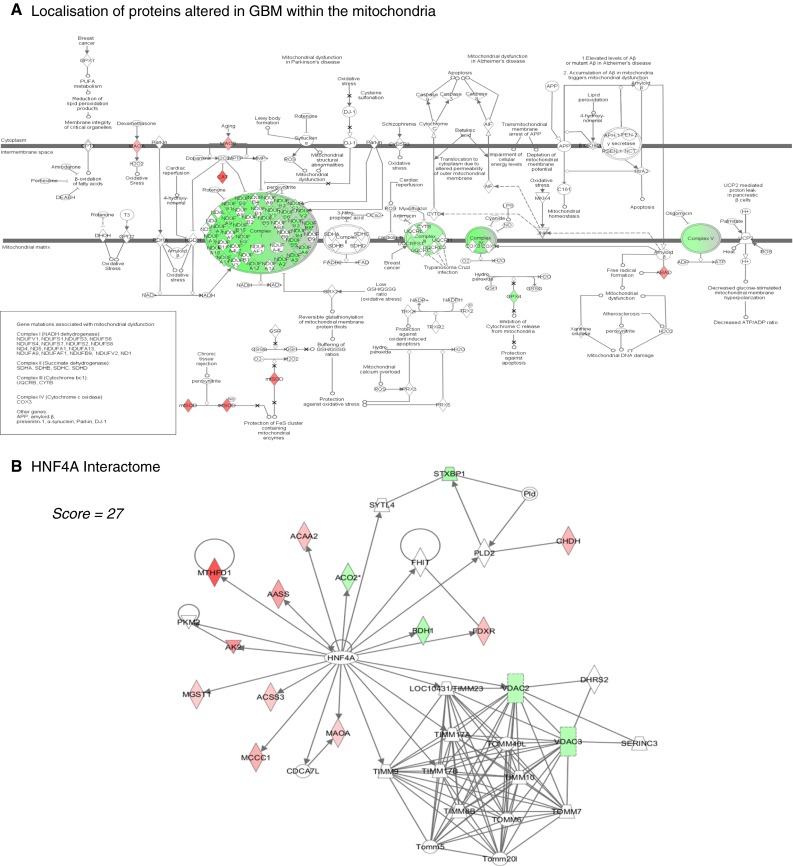
Interactome analysis of mitochondrial proteins altered in GBM. Putative localization of many of the proteins altered in GBM within the mitochondria (based upon the mitochondrial canonical pathway in Ingenuity Pathway Analysis (IPA); www.ingenuity.com). Note particularly the large number of proteins that are significantly less abundant in *green* (*p* < 0.05 and a ratio GBM/control <0.5) in GBM which are localised to the Electron Transport Chain Complex 1 (23 proteins). Proteins highlighted in *red* are significantly more abundant in GBM. Protein–protein interactions between mitochondrial proteins altered in GBM. Each node (*shape*) represents a protein and its association with other proteins is represented by a *line*. Nodes have different shapes that represent different molecule types, for example transcription factors, enzymes, kinases and phosphatases (refer to Ingenuity Systems Software for detailed node information). A high scoring interactome generated by IPA with the nuclear transcription factor HNF4A as an inserted hub protein (*no colour*). HNF4A linking 10 of the proteins increased (*red*) in GBM and 5 of the proteins decreased (*green*) in GBM. For details of all of the protein–protein interactomes generated by IPA from the 117 mitochondrial proteins altered, see supplementary information S6 (protein lists) and S7 (diagrammatic representations of networks)

To further interrogate the proteomic data, putative interactions (“interactomes”) between mitochondrial proteins significantly altered in GBM were identified using IPA (supplementary S6) [26]. The seven high-scoring networks (score >20) are depicted in Fig. 2 and supplementary S7. Three of the highest scoring interactomes were related to components of the ETC, notably complex-I, IV and V and a further interactome described protein–protein interactions between the ETC and Synuclein (*inter alia*). The interactome dominated by Complex-IV proteins also highlighted a cluster of isocitrate-dehydrogenase proteins (IDH3A, IDH3G and IDH3B), decreased in GBM. A high scoring network with the nuclear transcription factor HNF4A as an inserted hub protein links 10 proteins increased in GBM as well as 5 proteins reduced in GBM (Fig. 2b). The other two interactomes included one with proteins interacting with MYC and creatine-kinase, and another with interactions between proteins involved in ion-transport and related processes. The proteins involved in protecting the cells from oxidative damage (for example, CAT, SOD, PRDX1, GPX4) do not directly interact with each other despite similar functional roles but these appear in networks where their interactions with other proteins (altered in GBM) have been described.

### Morphology of mitochondria in GBM

**Fig. 3:**
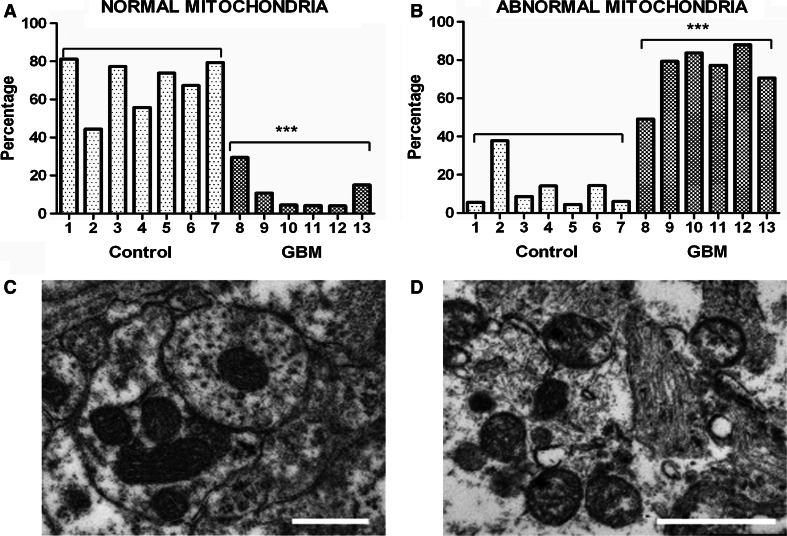
Morphology of mitochondria in GBM. The morphology of ~150 mitochondria was assessed in each of 6 GBM and 7 peritumoural control samples using Electron Microscopy (EM). **a** Percentage of normal mitochondria (i.e. where cristae are visible throughout the mitochondria, or in at least 50 % of the mitochondrial interior area) in peritumoural control and GBM samples (*each bar* represents one sample; *** *p*-value = 0.0001); **b** Percentage of abnormal mitochondria (i.e. with very few cristae, interior matrix condensed and dark or round swollen with interior missing) in peritumoural control and GBM samples (please see supplementary information S3 for more details; *** *p*-value = 0.0001). **c**, **d** Representative EM images of normal and abnormal mitochondria respectively. The *scale bars* represent 0.5 μm

The ultrastructure of mitochondria in GBM was quantitatively assessed and compared to control brain using Electron Microscopy (EM). In peritumoural-control brain, the ultrastructure of 69 % (range 44–81 %) of mitochondria analysed were normal (i.e. cristae visible with no swelling or condensed matrix) and 16 % (6–30 %) of mitochondria were abnormal (Fig. 3). In GBM, 11 % (4–30 %) of mitochondria were normal and 75 % (49–88 %) of mitochondria were abnormal (Fig. 3). 15 % of mitochondria in peritumoural-control brain and 14 % in GBM could not be unambiguously classified as either normal or abnormal.

## Discussion

This study provides a comprehensive proteomic and morphological characterisation of mitochondria in GBM. Numerous alterations in the levels of mitochondrial proteins were detected in GBM compared to control brain. Multiple proteins associated with oxidative damage were up-regulated in GBM and multiple proteins involved in energy metabolism were down-regulated. In addition a much greater prevalence of cristolysis was observed in GBM compared to control brain mitochondria by quantitative assessment of EM images. The abnormal mitochondrial ultrastructure could underlie the shift in energy generating pathways in GBM for cell survival and progression.

The role of reactive oxygen species (ROS) and antioxidants in cancer is highly complex. ROS can cause DNA damage that generates pro-oncogenic mutations, but a build up of ROS and damaged proteins in the mitochondria can also trigger apoptosis and autophagy [19]. ROS are a by-product of aerobic ATP generation. Increases in GBM in CAT, PRDX1, PRDX4 and SOD2 and a decrease in GPX4 may be a response to the increased ROS present due to the high energy demands of the GBM. Peroxiredoxin antioxidants are increased in various solid tumours [20–22] and PRDX1 is up-regulated in GBMs compared to low-grade gliomas. Peroxiredoxins 1 and 4 form a heterodimer and play a key role in regulating nuclear factor kB (NFkB) activity. NFkB is a transcription factor that modulates oncogenesis, tumour progression and chemotherapy resistance in a range of cancers [23, 24]. CAT is an enzyme that converts H_2_O_2_ to H_2_O and O_2_ and plays a multifaceted role in pro- and anti-apoptotic pathways. Over-expression of CAT decreases ROS levels thereby reducing apoptosis, but also decreases sensitivity to tumour necrosis factor alpha (TNFα) (by reducing H_2_O_2_ [25]) which leads to increased resistance to apoptosis. SOD2 plays a dual role in tumourigenic progression, but generally overexpression of SOD2 enhances the metastatic phenotype that is reversed by efficient H_2_O_2_ scavenging [26]. The reduction of GPX4 is pro-oncogenic since GPX4 has been shown to halt tumour proliferation and progression [27]. Antioxidants in healthy cells protect against tumour genesis by preventing oxidative damage but in cells that are already aberrant inhibition of these antioxidants could generate catastrophic damage by inducing apoptosis [28].

Energy pathways (comprising TCA cycle, ETC, lipid and amino acid metabolism) were also found severely altered in this study, in agreement with aberrant energy metabolism in gliomas and other cancers [8, 29–32]. Multiple protein alterations integral to energy production prior to the ETC were detected (for example, AK2, CKMT1B, GATM, GLS, HK1, ME2, ME3, SUCLA2 and SUCLG2), with these changes emphasising a disruption rather than a coordinated response. An increase in ME2 was found which converts malic acid and NAD + *into* pyruvate and NADH, increasing levels of NADH for the ETC [33]. SUCLG2 which catalyses GTP, succinate and CoA *into* GDP, phosphate and succinyl-CoA was markedly increased. In contrast, a decrease was seen in the ATP isoform SUCLA2 which reduces availability of succinyl-CoA [34]. The increase in GATM, which catalyses production of creatine precursor, suggests an increase in creatine which can act as a phosphate store in the brain to rapidly replenish ATP supplies [35]. Another altered protein, AK2, is found in the intermembrane space of mitochondria and catalyses the reversible reaction ATP and AMP *into* 2ADP. When AK2 is translocated from the nucleus to the cytoplasm it triggers apoptosis and AK2 knock-down decreases apoptosis. An increase in AK2 in the mitochondria may favour a decrease in translocation and therefore a decrease in apoptosis promoting tumour growth [36].

The ETC consists of five (I–V) transmembrane protein complexes that act in consort to transfer electrons and drive protons into the intermembrane space to create a proton motive gradient across the inner mitochondrial membrane and generate ATP [37]. Numerous ETC proteins not previously identified in whole cell lysate studies were detected in our study and the majority of these were down-regulated in GBM (including 23 Complex-I proteins) indicating that Complex-I function and oxidative phosphorylation (normal aerobic respiration) is reduced in GBM.

Proteins involved in lipid metabolism (for example, ACADM, ACOT7, DECR1, SCP2) and amino acid metabolism (for example, AASS, LAP3, MTHFD1, SHMT2) were up-regulated. ACADM and DECR1 are enzymes required for the mitochondrial β oxidation of lipids [38, 39] and SCP2 is a lipid transfer molecule that ensures a sufficient supply of lipids to the mitochondria [40]. AASS catalyses the metabolism of lysine to glutamate which can be converted to α-ketoglutarate, a component of the TCA cycle [41]. SHMT2 is a protein responsive to the pro-oncogenic gene cMyc and catalyses the conversion of serine to glycine with a single carbon by-product for cell proliferation [42].

Network analysis highlighted a cluster of altered mitochondrial proteins associated with the nuclear transcription factor HNF4A. Although frequently described as a tumour-suppressor gene, HNF4A plays a role in cancer initiation and intracellular protection against cancer-related ROS production [9]. HNF4A has also recently been shown to be expressed and regulated in meningioma brain tumours [43]. HNF4A interacts with proteins that are little characterised such as ACSS3, BDH1 and FDXR, and highlights the need for further functional investigations of these proteins in tumour pathogenesis.

A number of mitochondrial proteins with relevance to tumour pathophysiology were altered, including BSG, SNCB and three Isocitrate dehydrogenase 3 forms (IDH3A, IDH3B, and IDH3G). BSG is associated with tumour invasiveness, metastasis, drug resistance and glycolysis [44] and activates multiple pathways including NFkB and JNK which stimulate matrix metalo-proteinases (MMP) [45]. MMPs promote invasion by breaking down the intracellular matrix. BSG levels in GBM tissue were more than twice that of control indicating a contributory role in tumour progression providing a potential therapeutic target. Calveolin1 is an effective inhibitor of BSG by reducing BSG glycosylation [46]. SNCB, known for its role in Alzheimer’s disease, was decreased in GBM tissue 4-fold compared to peritumoural-control tissue and inhibits phospholipase D2 (PLD2) [47] which is oncogenic [48]. Restoration of SNCB levels may suppress tumour progression. Downregulated isoforms of IDH3 were also noted, and IDH1 and IDH2 mutations have recently been reported in gliomas [49]. IDH1 (cytoplasmic) and IDH2 (mitochondrial) catalyse the reaction *Isocitrate*-*dehydrogenase* + *NADP* ^+^ *→2*-*α*-*ketoglutarate*(*αKG*) + *CO*_*2*_ + *NADPH*. IDH1 mutations are present in low but not high-grade gliomas and preferentially occur in young patients with improved prognosis [50]. Both IDH1 and IDH2 mutations have a loss-of-function and gain-of-function consequence. No mutations of IDH3, which normally catalyse the same reaction as IDH1/IDH2 (but use NAD^+^ as substrate) have been noted in gliomas, so the consequence of its downregulation can only be extrapolated from reports on IDH1/IDH2. The reduction in IDH3 would produce the loss-of-function phenotype without the gain. Loss-of-function causes decreased α-KG and NADH, and the decreased α-KG results in an increased hypoxia inducible factor 1 alpha (HIF-1α) which promotes glioma development [51].

We describe for the first time systematic differences in the mitochondrial proteome in GBM relative to peritumoural-control tissue. Although casual perusal of the data (for example Fig. 1b) offers tantillising suggestions of heterogeneity of particular protein levels in peritumoural control or GBM, the variance within the two groups are similar to that observed in our other studies (mouse, cell culture). The small sample size (groups of 6) relative to the number of proteins assessed (256) precludes meaningful subgroup analysis, of for example IDH1 mutations, methylation status and age related changes, despite its considerable clinical importance. The clear demonstration of biologically coherent changes in mitochondrial proteins in GBM highlights the importance of further proteomic analysis of this brain pathology.

## Electronic supplementary material

Below is the link to the electronic supplementary material.

S1 Clinical details for the peritumoural control and GBM samples analysed. [A] Clinical details (patient age, gender and pathology) for the peritumoural control and GBM samples analysed by LC–MS. Peritumoural control tissue was harvested from patients undergoing various types of brain tumour surgery (see ‘patient pathology’) with the exception of patient ID 1 (see below). A BrainLAB MRI guided system was used (merged T1 contrast enhanced plus T2) to determine ‘peritumoural brain’. The peritumoural control tissue was harvested before tumour removal to minimise brain movement artefact. Abbreviations: OII = oligodendroglioma, AII = WHO grade II astrocytoma, AIII = WHO grade III astrocytoma. The patient (sample ID 1) with the colloidal cyst had the brain tissue specimen taken using a transcortical approach to the ventricle. All the fresh tissue biopsies for proteomic analysis were harvested by a single neurosurgeon and collected in the surgical theatre where they were immediately frozen on dry-ice. [B] Correlation analysis of log protein levels in individual peritumoural control samples (y-axis) relative to mean log protein levels in peritumoural control group (x-axis). Each point represents the abundance of a protein. Sample IDs are the same as in supplementary Table 1. The global proteomic pattern in individual samples cannot be differentiated from that of the group mean. In the control material (‘peritumoural control brain’) the proteomic findings were qualitatively and quantitatively consistent despite heterogeneity of aetiology. [C] Correlation analysis of log protein levels in individual GBM samples (y-axis) relative to mean log protein levels in peritumoural control group (x-axis). Each point represents the abundance of a protein. Sample IDs are the same as in supplementary Table 1. All GBM samples were harvested from patients with primary GBM and were confirmed by a consultant neuropathologist. Resections in all cases were maximal. [D] Clinical details (patient age, gender and pathology) for the peritumoural control and GBM samples analysed by Electron Microscopy. Supplementary material 1 (DOC 354 kb)

S2: Data for all proteins in the enriched mitochondrial fractions identified by ≥2 peptides by LC–MS. Proteins are listed by category: 1) mitochondrial proteins significantly increased in GBM (*p* ≤ 0.05, ≥2-fold change), 2) mitochondrial proteins significantly decreased in GBM (*p* ≤ 0.05, ≥2-fold change), 3) non-mitochondrial proteins significantly increased in GBM (*p* ≤ 0.05, ≥2-fold change), 4) non-mitochondrial proteins significantly decreased in GBM (*p* ≤ 0.05, ≥2-fold change), 5) all other proteins identified in GBM (no significant change). The protein accession number (IPI), gene name, p-value, magnitude of protein response, the number of peptides for protein identification and the Mascot identification score are listed for each protein. The raw data are available on the public data repository PRIDE (see methods). Supplementary material 2 (DOC 1004 kb)

S3: Morphological Classification of Mitochondria. The morphology of mitochondria in GBM (relative to control brain) was assessed using Electron Microscopy and 6 a priori categories of morphology represented by images A – F. The asterisks indicate the mitochondria that represent the category in each image where there is more than one mitochondrion present. Image [A] represents ‘normal’ mitochondria, where cristae are visible throughout the mitochondria or in at least 50 % (as judged qualitatively) of the mitochondria interior area. Image [B] shows a mitochondrion that is predominantly normal but shows few cristae occupying less than 50 % of the interior area. Images [C, D, E,] depict ‘abnormal’ mitochondria with very few cristae, interior matrix condensed and dark or round swollen with interior missing. The images show a progression of morphological disruption with image [C] showing mitochondria that are vesicular, [D] showing mitochondria that are part swollen and part vesicular, and [E] showing mitochondria that are significantly swollen. Image [F] shows mitochondria that the evaluator could not classify due to undetermined abnormality. These might be arising from artefacts i.e. cutting, fixation or obstruction. Images [B & F] represent mitochondria that could not be straightforwardly classified as ‘normal’ or ‘abnormal’. The scale bar represents 0.5 μm. Supplementary material 3 (PPT 294 kb)

S4: Integrity of enriched mitochondrial fractions. Western blot analysis of 3 mitochondrial membrane proteins (VDAC1 localised to the outer mitochondrial membrane; COXI and COXIV localised to the inner mitochondrial membrane) in the supernatant (s) and mitochondrial (m) fractions generated for LC–MS (n = 6 pertitumoural control; n = 6 GBM; see methods section on mitochondrial fractionation). All 3 proteins are present in the mitochondrial fractions (m) but absent in the supernatant fractions (s) in both GBM and peritumoural control. There is no evidence that there are differential effects of fractionation in GBM and peritumoural control brain tissue. Supplementary material 4 (PPT 174 kb)

S5: Summary of LC–MS identifications from GBM and peritumoural control brain mitochondrial fractions. Total number of proteins identified (with ≥2 peptides) in the mitochondrial enriched fractions extracted from GBM and peritumoural control brain; total number of proteins recognised by DAVID software; total number of proteins identified as being mitochondrially-associated based on the GO designation *mitochondrion*; and total number of dysregulated mitochondrial proteins (*p* ≤ 0.05, ≥2-fold change) in GBM compared to peritumoural control brain. Supplementary material 5 (DOC 27 kb)

S6: Putative interactions between mitochondrial proteins altered in GBM. Protein–protein interaction networks (‘interactomes’) generated by Ingenuity Pathway Analysis (http://www.ingenuity.com). The proteins highlighted in bold are the mitochondrial proteins found significantly altered in the study (*t*-test *p* ≤ 0.05, ≥2-fold change) in GBM, and are termed ‘Focus Molecules’. Proteins not in bold have been inserted by IPA and are proteins (not exclusive to mitochondria) that interact with the focus molecules. The coloured arrows indicate the direction of response of the focus molecule in GBM (red = increased; green = decreased). Each network is assigned a score by IPA. Network scores are putatively a measure of probability for the network (but see Deighton et al. [16], for critical analysis of this issue). Supplementary material 6 (DOC 47 kb)

S7: Mitochondrial Interactomes in GBM. Visual representations of putative protein–protein interactions in the high scoring networks (‘ETC Networks 1-3 and 5’ (A-D); ‘MYC + Creatine Kinase’ network (E); and ‘Ion Transport’ network (F); as listed in S4) generated by IPA from mitochondrial proteins altered in GBM. In the network, each node (shape) represents a protein and its association with other proteins is represented by a line. Nodes have different shapes that represent different molecule types, for example transcription factors, enzymes, kinases and phosphatases (refer to Ingenuity Systems Software for detailed node information). Mitochondrial proteins or ‘nodes’ with a coloured background were regulated in the study (green = decreased; red = increased) whilst other interacting proteins with no background are proteins not detected in this study that have been inserted by IPA (and are not exclusive to mitochondria) to produce a highly connected network. The solid lines represent direct interactions or associations between proteins. Supplementary material 7 (PPT 819 kb)

## References

[1] Anderson E, Grant R, Lewis SC, Whittle IR (2008). Randomized phase III controlled trials of therapy in malignant glioma: where are we after 40 years?. Br J Neurosurg.

[2] Deighton RF, McGregor R, Kemp J, McCulloch J, Whittle IR (2010). Glioma pathophysiology: insights emerging from proteomics. Brain Path.

[3] Ordys BB, Launay S, Deighton RF, McCulloch J, Whittle IR (2010). The role of mitochondria in glioma pathophysiology. Mol Neurobiol.

[4] Griguer CE, Oliv CR (2011). Bioenergetics pathways and therapeutic resistance in gliomas: emerging role of mitochondria. Curr Pharm Des.

[5] Bernard G, Rossignal R (2008). Ultrastructure of the mitochondrion and its bearing on function and bioenergetics. Antioxid Redox Signal.

[6] Furnari FB, Fenton T, Bachoo RM (2007). Malignant astrocytic glioma: genetics, biology and pathways to treatment. Genes Dev.

[7] Ziegler DS, Kung AL, Kieran MW (2008). Anti-apoptosis mechanisms in malignant gliomas. J Clin Oncol.

[8] Seyfried TN, Mukherjee P (2005). Targeting energy metabolism in brain cancer: review and hypothesis. Nutr Metab.

[9] Darsigny M, Babeu JP, Seidman EG (2010). Hepatocyte nuclear factor-4alpha promotes gut neoplasia in mice and protects against the production of reactive oxygen species. Cancer Res.

[10] James R, Searcy JL, LeBihan T (2012). Proteomic analysis of mitochondria in APOE transgenic mice and in response to an ischemic challenge. J Cereb Blood Flow Metab.

[11] Le Bihan T, Grima R, Martin S, Forster T, Le Bihan Y (2010). Quantitative analysis of low-abundance peptides in HeLa cell cytoplasm by targeted liquid chromatography/mass spectrometry and stable isotope dilution: emphasising the distinction between peptide detection and peptide identification. Rapid Commun Mass Spectrom.

[12] Herrmann AG, Deighton RF, LeBihan T, McCulloch MC, Searcy JL, Kerr LE, Kerr LE, McCulloch J (2013). Adaptive changes in the neuronal proteome: mitochondrial energy production, endoplasmic reticulum stress, and ribosomal dysfunction in the cellular response to metabolic stress. J Cereb Blood Flow Metab.

[13] Barsnes H, Vizcaino JA, Eidhammer I, Martens L (2009). PRIDE converter: making proteomics data-sharing easy. Nat Biotechnol.

[14] da Huang W, Sherman BT, Lempicki RA (2009). Systematic and integrative analysis of large gene lists using DAVID bioinformatics resources. Nat Protoc.

[15] Dennis G, Sherman BT, Hosack DA (2003). DAVID: database for annotation, visualisation, and integrated discovery. Genome Biol.

[16] Deighton RF, Kerr LE, Short DM, Allerhand M, Whittle IR, McCulloch J (2010). Network generation enhances interpretation of proteomic data from induced apoptosis. Proteomics.

[17] Griffiths IR, Duncan ID, McCulloch M (1981). Shaking pup: a disorder of central myelination in the spaniel dog. II. Ultrastructural observations on the white matter of cervical spinal cord. J Neurocytol.

[18] Forner F, Arriaga EA, Mann M (2006). Mild protease treatment as a small-scale biochemical method for mitochondria purification and proteomic mapping of cytoplasm-exposed mitochondrial proteins. J Proteome Res.

[19] Li Z-Y, Yang Y, Ming M, Liu B (2011). Mitochondrial ROS generation for regulation of autophagic pathways in cancer. Biochim Biophys Res Commun.

[20] Noh DY, Ahn SJ, Lee RA, Kim SW, Park IA, Chae HZ (2001). Overexpression of peroxiredoxin in human breast cancer. Anticancer Res.

[21] Kinnula VL, Lehtonen S, Sormunen R (2002). Overexpression of peroxiredoxins I, II, III, V, and VI in malignant mesothelioma. J Pathol.

[22] Park JH, Kim YS, Lee HL (2006). Expression of peroxiredoxin and thioredoxin in human lung cancer and paired normal lung. Respirology.

[23] Jin DY, Chae HZ, Rhee SG, Jeang KT (1997). Regulatory role for a novel human thioredoxin peroxidase in NF-kappaB activation. J Biol Chem.

[24] Cortes SM, Rodriguez FV, Sanchez PI, Perona R (2008). The role of the NFkappaB signalling pathway in cancer. Clin Transl Oncol.

[25] Lupertz RY, Chovolou Y, Kampkotter A, Watjen W, Kahl R (2008). Catalase overexpression impairs TNF-alpha induced NF-kappaB activation and sensitizes MCF-7 cells against TNF-alpha. J Cell Biochem.

[26] Hempel N, Carrico PM, Melendez JA (2011). Manganese superoxide dismutase (Sod2) and redox-control of signalling events that drive metastasis. Anticancer Agents Med Chem.

[27] Brigelius-Flohe R, Kipp A (2009). Glutathione peroxidases in different stages of carcinogenesis. Biochim Biophys Acta.

[28] Acharya A, Das I, Chandhok D, Saha T (2010). Redox regulation in cancer: a double-edged sword with therapeutic potential. Oxid Med Cell Longev.

[29] Warburg O (1931). The metabolism of tumours.

[30] Shaw RJ (2006). Glucose metabolism and cancer. Curr Opin Cell Biol.

[31] Moreno-Sanchez R, Rodriguez-Enriquez S, Saavedra E, Marin-Hernandez A, Gallardo-Perez JC (2009). The bioenergetics of cancer: is glycolysis the main ATP supplier in all tumour cells?. BioFactors.

[32] Cuezva JM, Ortega AD, Willers I, Sanchez-Cenizo L, Aldea M, Sanchez-Arago M (2009). The tumour suppressor function of mitochondria: translation into the clinics. Biochim Biophys Acta.

[33] Yang Z, Lanks CW, Tong L (2002). Molecular mechanism for the regulation of human mitochondrial NAD(P)+-dependent malic enzyme by ATP and fumarate. Structure.

[34] Miller C, Wang L, Ostergaard E, Dan P, Saada A (2011). The interplay between SUCLA2, SUCLG2 and mitochondrial DNA depletion. Biochim Biophys Acta.

[35] Cullen ME, Yuen AH, Felkin LE (2006). Myocardial expression of the arginine:glycine amidinotransferase gene is elevated in heart failure and normalised after recovery: potential limitations for local creatine synthesis. Circulation.

[36] Lee HJ, Pyo JO, Oh Y (2007). AK2 activates a novel apoptotic pathway through formation of a complex with FADD and caspase-10. Nat Cell Biol.

[37] Rich PR, Marechal A (2010). The mitochondrial respiratory chain. Essays Biochem.

[38] Thorpe CK, Kim JJ (1995). Structure and mechanism of action of the acyl-CoA dehydrogenases. FASEB.

[39] Alphey MS, Yu W, Byres E, Li D, Hunter WN (2005). Structure and reactivity of human mitochondrial 2,4-dienoyl-CoA reductase:enzyme-ligand interactions in a distinctive short chain reductase active site. J Biol Chem.

[40] Gallegos AM, Atshaves BP, Storey SM (2001). Gene structure, intracellular localization, and functional roles of sterol carrier protein-2. Prog Lipid Res.

[41] Sacksteder KA, Biery BJ, Morrell JC (2000). Identification of the [alpha]-aminoadipic semialdehyde synthase gene, which is defective in familial hyperlysinemia. Am J Hum Genet.

[42] Nikiforov MA, Chandriani S, O’Connell B (2002). A functional screen for Myc-responsive genes reveals serine hydroxymethyltransferase, a major source of the one-carbon unit for cell metabolism. Mol Cell Biol.

[43] Herrmann A, Ooi J, Launay S, Searcy JL, Deighton RF (2011). Proteomic data in meningiomas: post-proteomic analysis can reveal novel pathophysiological pathways. J Neurooncol.

[44] Kanekura T, Chen X (2010). CD147/basigin promotes progression of malignant melanoma and other cancers. J Dermatol Sci.

[45] Venkatesan B, Valente AJ, Prabhu SD, Shanmugam P, Delafontaine P, Chandrasekar B (2010). Empirin activates multiple transcription factors in cardiomyocytes and induces interleukin-18 expression via Rac1-dependent PI3K/Akt/IKK/NF-kappaB and MKK7/JNK/AP-1 signalling. J Mol Cell Cardiol.

[46] Tang W, Chang SB, Hemler ME (2004). Links between CD147 function, glycosylation and caveolin-1. Mol Biol Cell.

[47] Payton JE, Perrin RJ, Woods WS, George JM (2004). Structural determinants of PLD2 inhibition by alpha-synuclein. J Mol Biol.

[48] Kang DW, Choi KY, Min S (2011). Phospholipase D meets Wnt signalling: a new target for cancer therapy. Cancer Res.

[49] Yan H, Parsons DW, Jin G (2009). IDH1 and IDH2 mutations in gliomas. N Engl J Med.

[50] Dang L, Jin S, Su SM (2010). IDH mutations in glioma and acute myeloid leukaemia. Trends Mol Med.

[51] Fu Y, Huang R, Zheng Y, Zhang Z, Liang A (2011). Glioma-derived mutations in isocitrate dehydrogenase 2 beneficial to traditional chemotherapy. Biochem Biophys Res Commun.

