# Supplementary material for: Interactions among mitochondrial proteins altered in glioblastoma
Source: J Neurooncol. 2014 Apr 13;118(2):247–56. doi: 10.1007/s11060-014-1430-5 (PMC4048470; doi:10.1007/s11060-014-1430-5)
Supplement: Supplementary file 1 — Clinical details for the peritumoural control and GBM samples analysed. [A] Clinical details (patient age, gender and pathology) for the peritumoural control and GBM samples analysed by LC–MS. Peritumoural control tissue was harvested from patients undergoing various types of brain tumour surgery (see ‘patient pathology’) with the exception of patient ID 1 (see below). A BrainLAB MRI guided system was used (merged T1 contrast enhanced plus T2) to determine ‘peritumoural brain’. The peritumoural control tissue was harvested before tumour removal to minimise brain movement artefact. Abbreviations: OII = oligodendroglioma, AII = WHO grade II astrocytoma, AIII = WHO grade III astrocytoma. The patient (sample ID 1) with the colloidal cyst had the brain tissue specimen taken using a transcortical approach to the ventricle. All the fresh tissue biopsies for proteomic analysis were harvested by a single neurosurgeon and collected in the surgical theatre where they were immediately frozen on dry-ice. [B] Correlation analysis of log protein levels in individual peritumoural control samples (y-axis) relative to mean log protein levels in peritumoural control group (x-axis). Each point represents the abundance of a protein. Sample IDs are the same as in supplementary Table 1. The global proteomic pattern in individual samples cannot be differentiated from that of the group mean. In the control material (‘peritumoural control brain’) the proteomic findings were qualitatively and quantitatively consistent despite heterogeneity of aetiology. [C] Correlation analysis of log protein levels in individual GBM samples (y-axis) relative to mean log protein levels in peritumoural control group (x-axis). Each point represents the abundance of a protein. Sample IDs are the same as in supplementary Table 1. All GBM samples were harvested from patients with primary GBM and were confirmed by a consultant neuropathologist. Resections in all cases were maximal. [D] Clinical details (patient age [file 11060_2014_1430_MOESM1_ESM.doc]

**Supplementary Information 1**

*Supplementary information 1 provides:*

**[A]** Detailed clinical information on the material used in the proteomic analyses. Correlation analysis (r^2^ and slope) for the peritumoural control samples suggests that the global proteomic pattern in no individual sample deviates from the overall peritumoural control group.

**[B]** Correlation analyses for the global proteomic pattern of individual samples of peritumoural control samples are provided.

**[C]** Correlation analyses for the global proteomic pattern of individual samples of GBM samples are provided.

**[D]** Detailed clinical information on the material used in the EM analysis.

A. Proteomic Samples

|  | **Sample ID** | **Age** | **Sex** | **Patient pathology** | **Correlation analysis** | |
| --- | --- | --- | --- | --- | --- | --- |
|  |  |  |  |  | **r^2^** | **Slope (m)** |
| PERITUMOURAL CONTROL  Cohort | 1 (C1) | 54 | M | Colloidal cyst | 0.96 | 1.04 |
|  | 2 (C2) | 51 | F | AII | 0.98 | 1.04 |
|  | 3 (C3) | 60 | M | GBM | 0.94 | 1.03 |
|  | 4* (C4) | 51 | F | OII | 0.94 | 0.97 |
|  | 5* (C5) | 26 | M | AIII | 0.97 | 1.02 |
|  | 6* (C6) | 74 | F | GBM | 0.97 | 1.02 |
| GBM  Cohort | 7 (T1) | 48 | M | GBM | 0.62 | 0.72 |
|  | 8 (T2) | 61 | F | GBM | 0.76 | 0.81 |
|  | 9 (T3) | 63 | F | GBM | 0.92 | 0.89 |
|  | 10* (T4) | 60 | M | GBM | 0.72 | 0.81 |
|  | 11* (T5) | 66 | M | GBM | 0.64 | 0.72 |
|  | 12* (T6) | 72 | M | GBM | 0.79 | 0.80 |

(***** supernatants analysed by western blotting)

**B. Peritumoural control brain proteomic profiles (relative to group mean)**

| **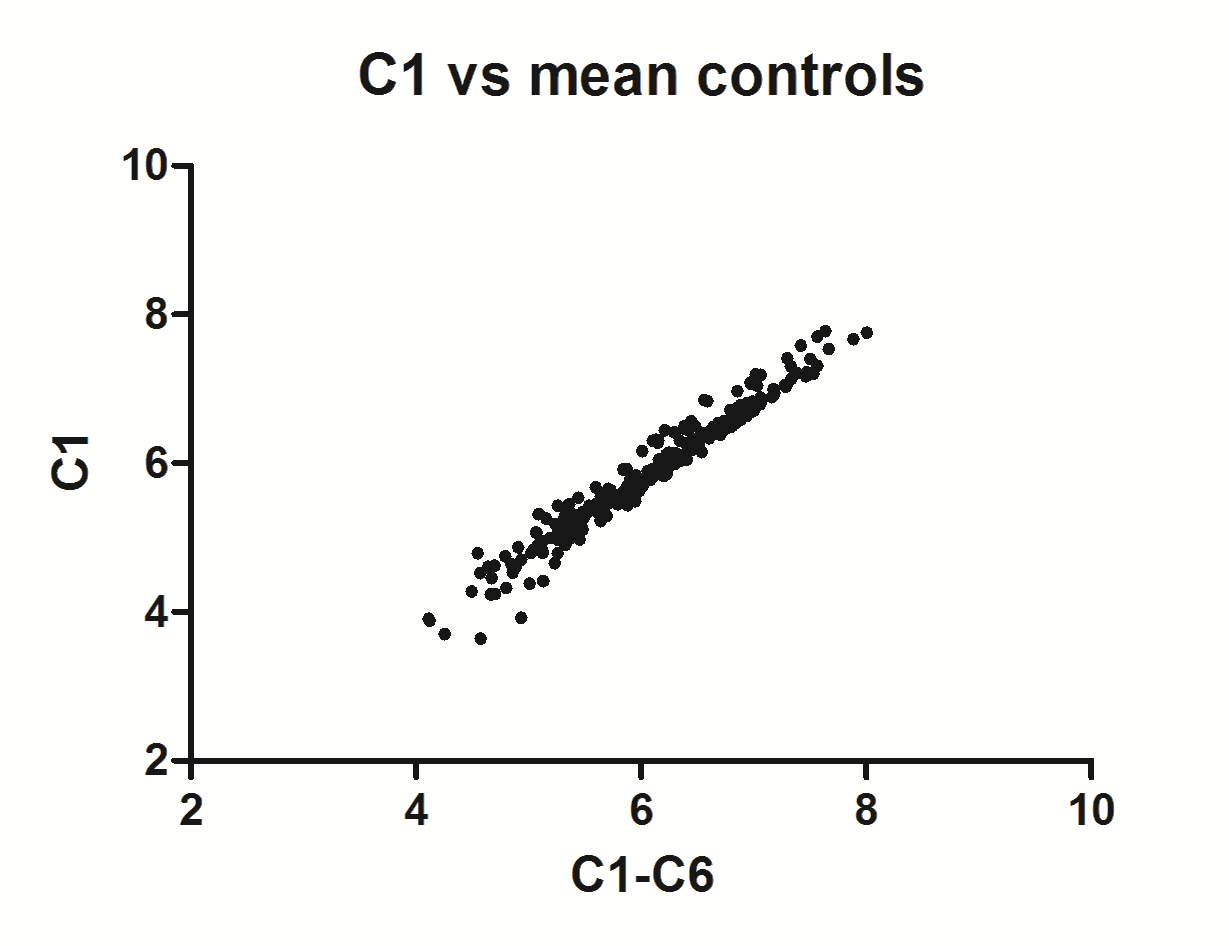**  r^2^=0.96 | **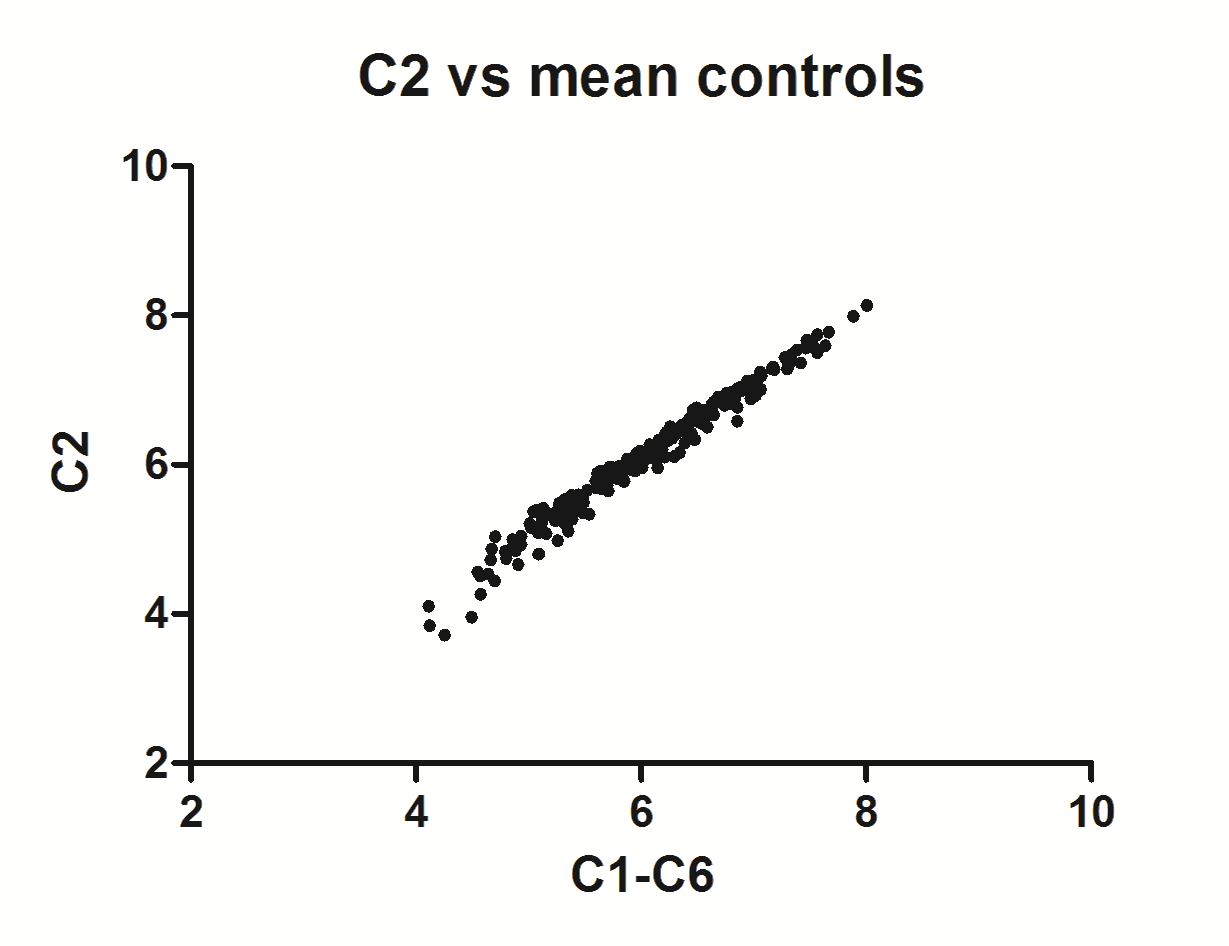**  r^2^=0.98 |
| --- | --- |
| **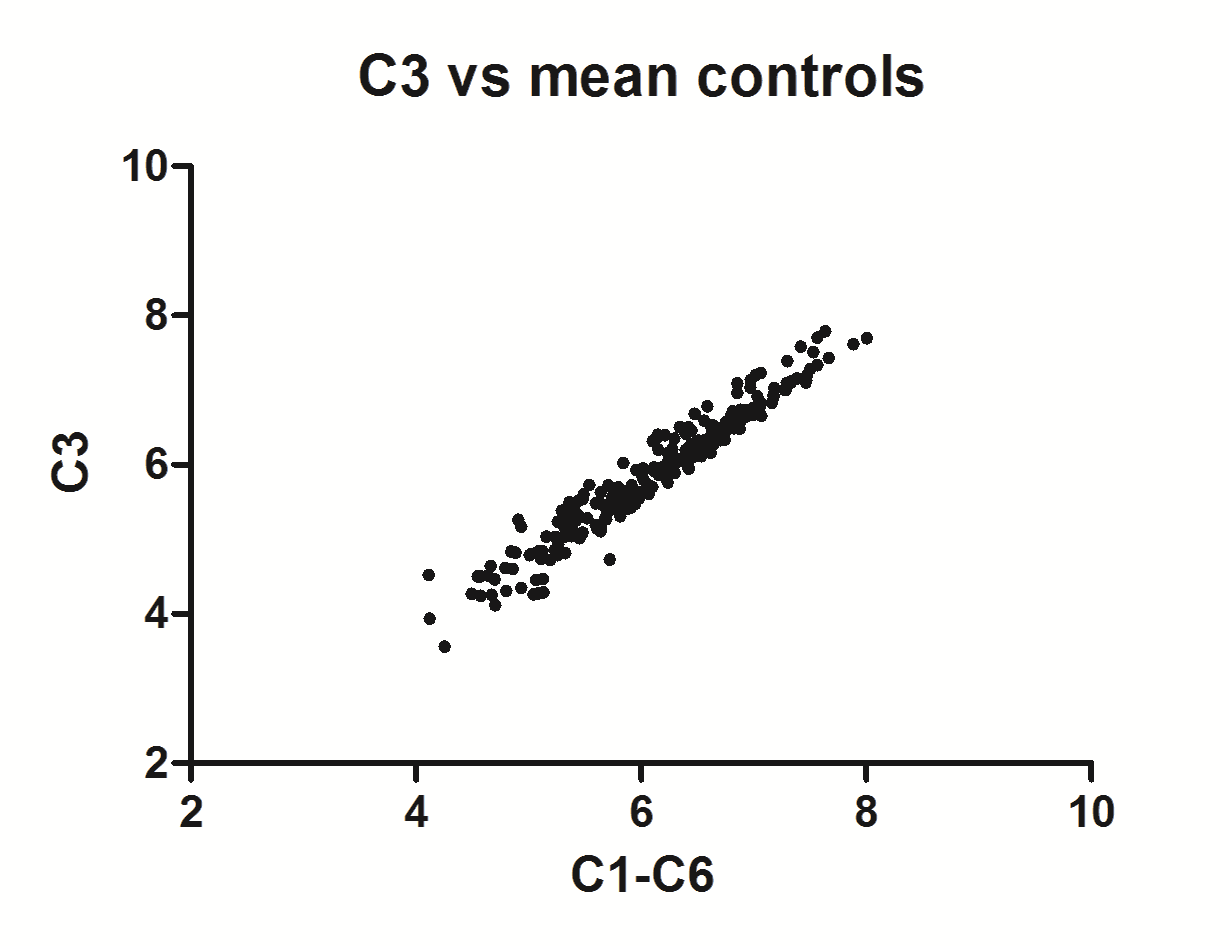**  r^2^=0.94 | **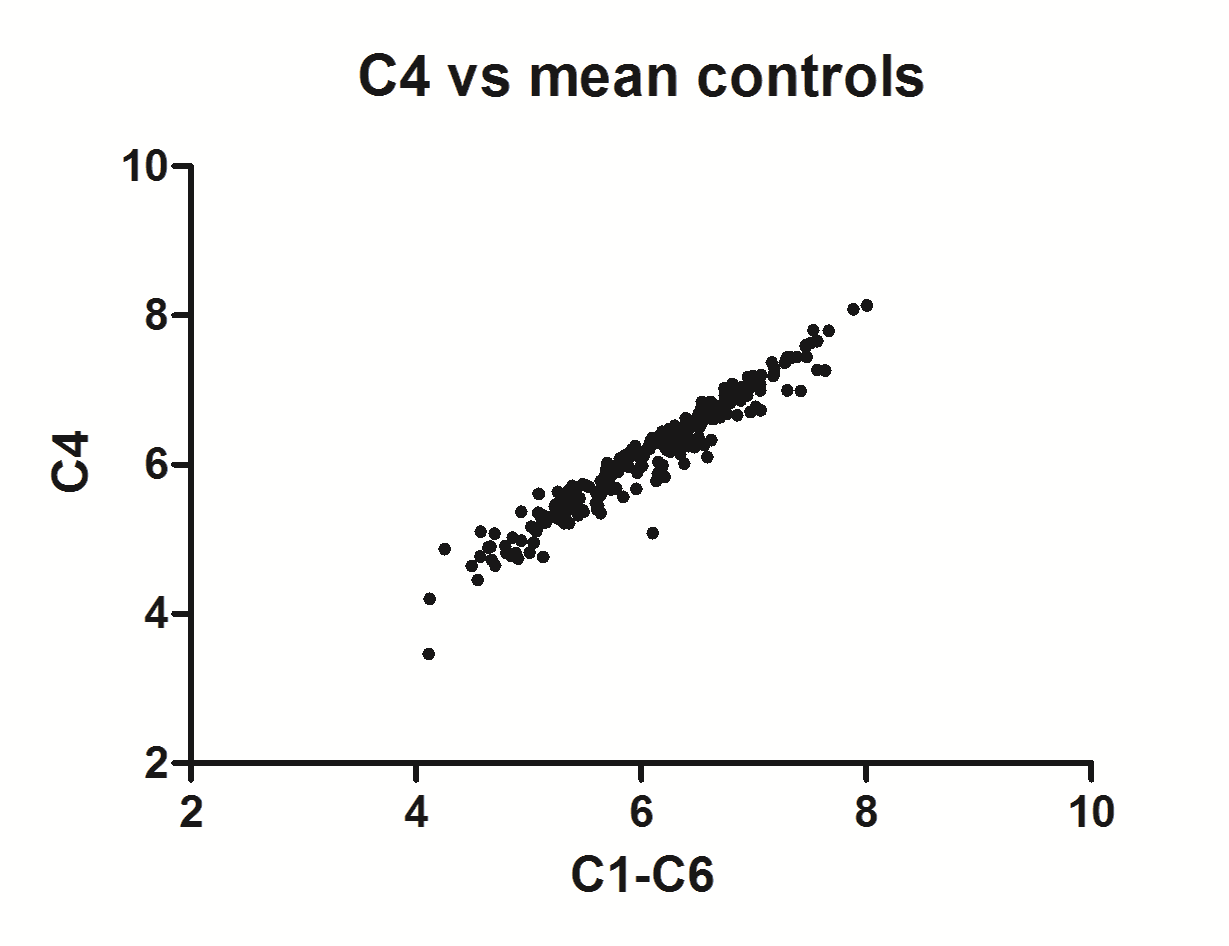**  r^2^=0.94 |
| **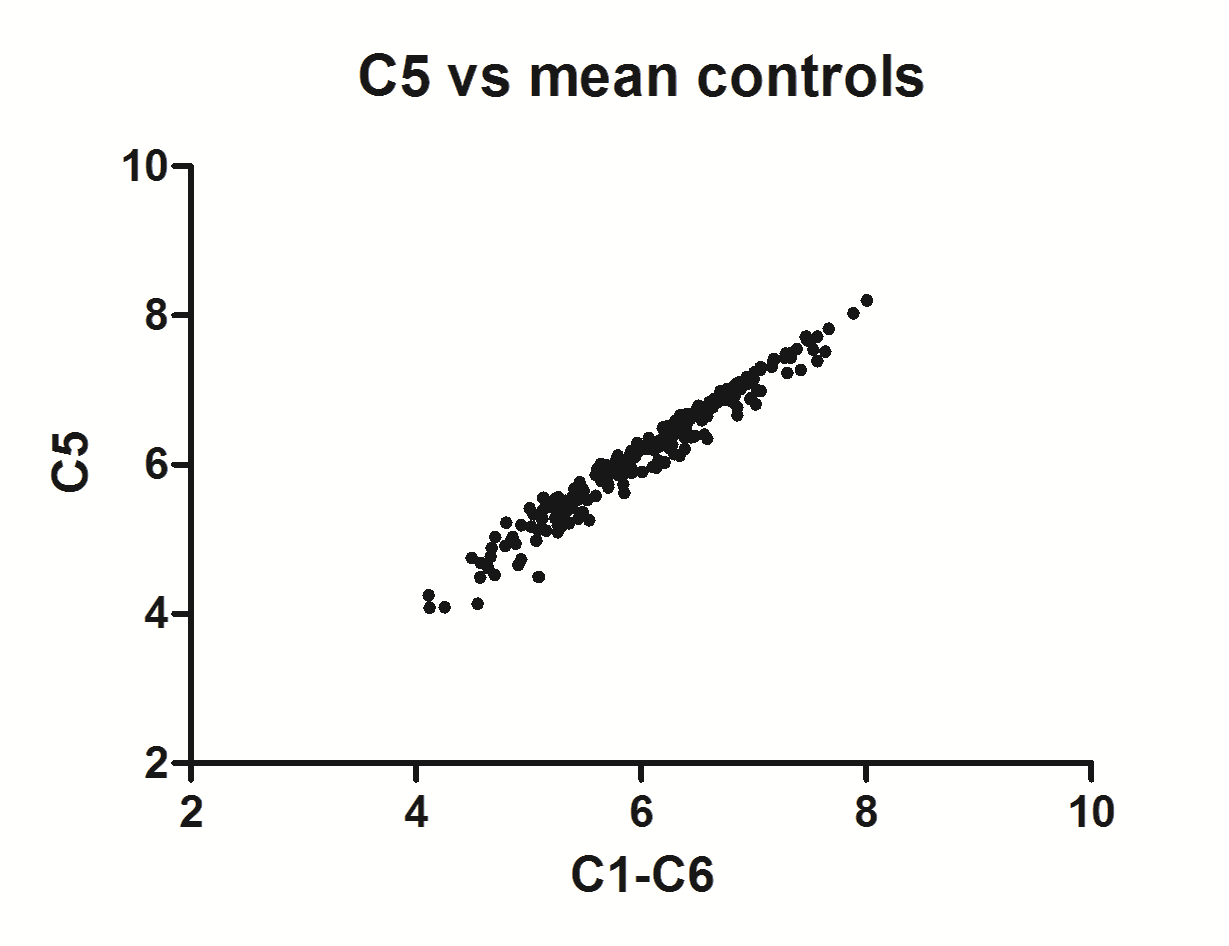** | **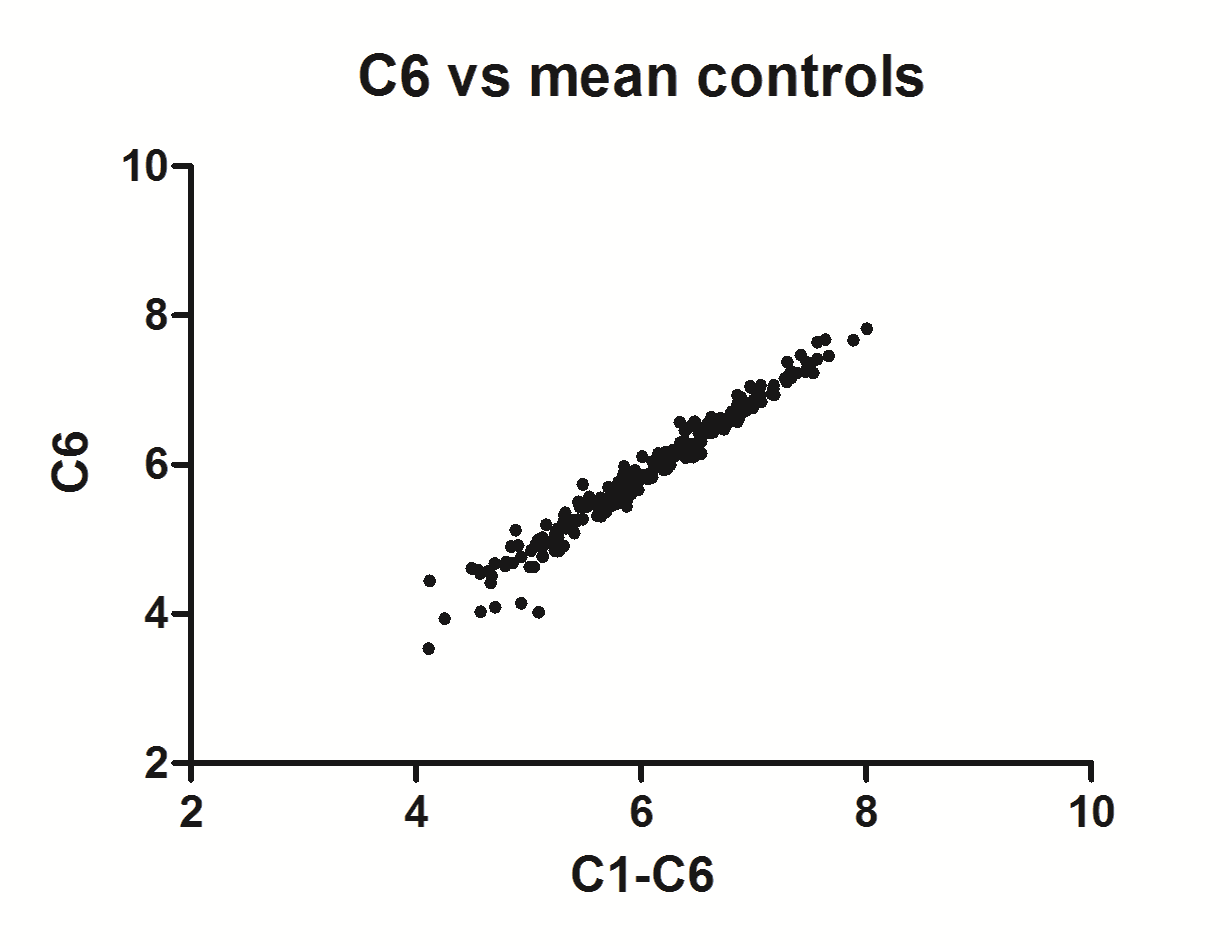**  r^2^=0.97 |

r^2^=0.97

**C. GBM proteomic profiles (relative to peritumoural brain group mean)**

| **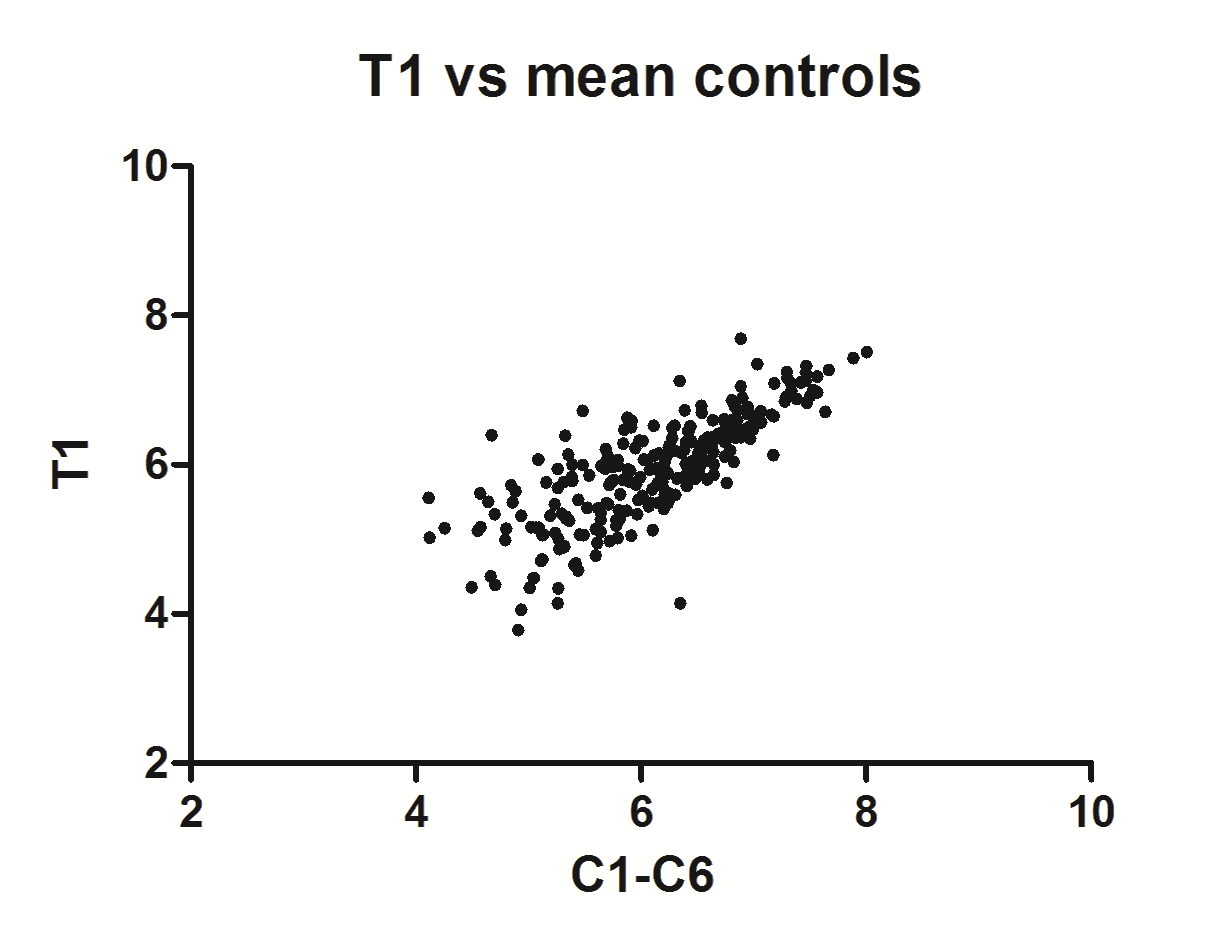**  r^2^=0.62 | **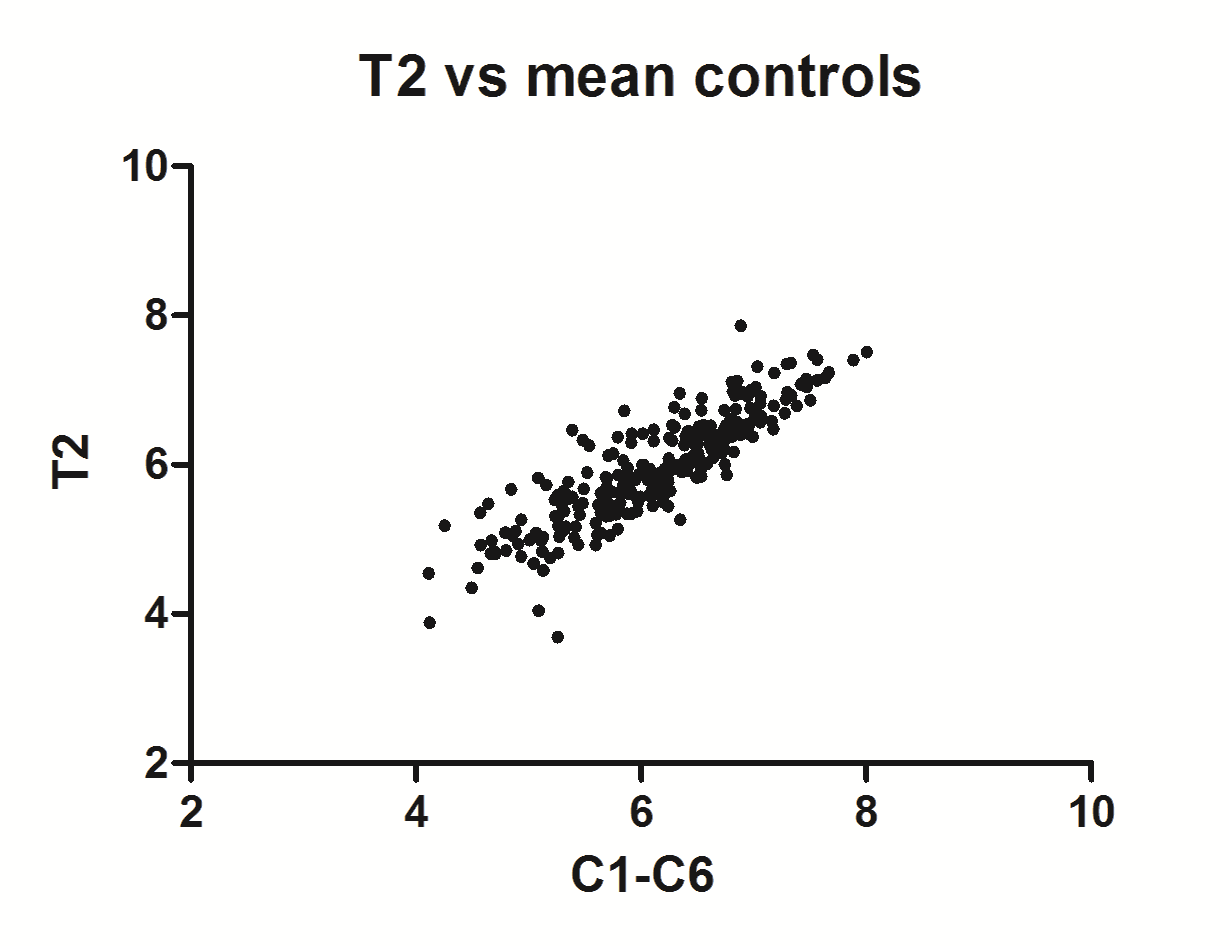**  r^2^=0.76 |
| --- | --- |
| **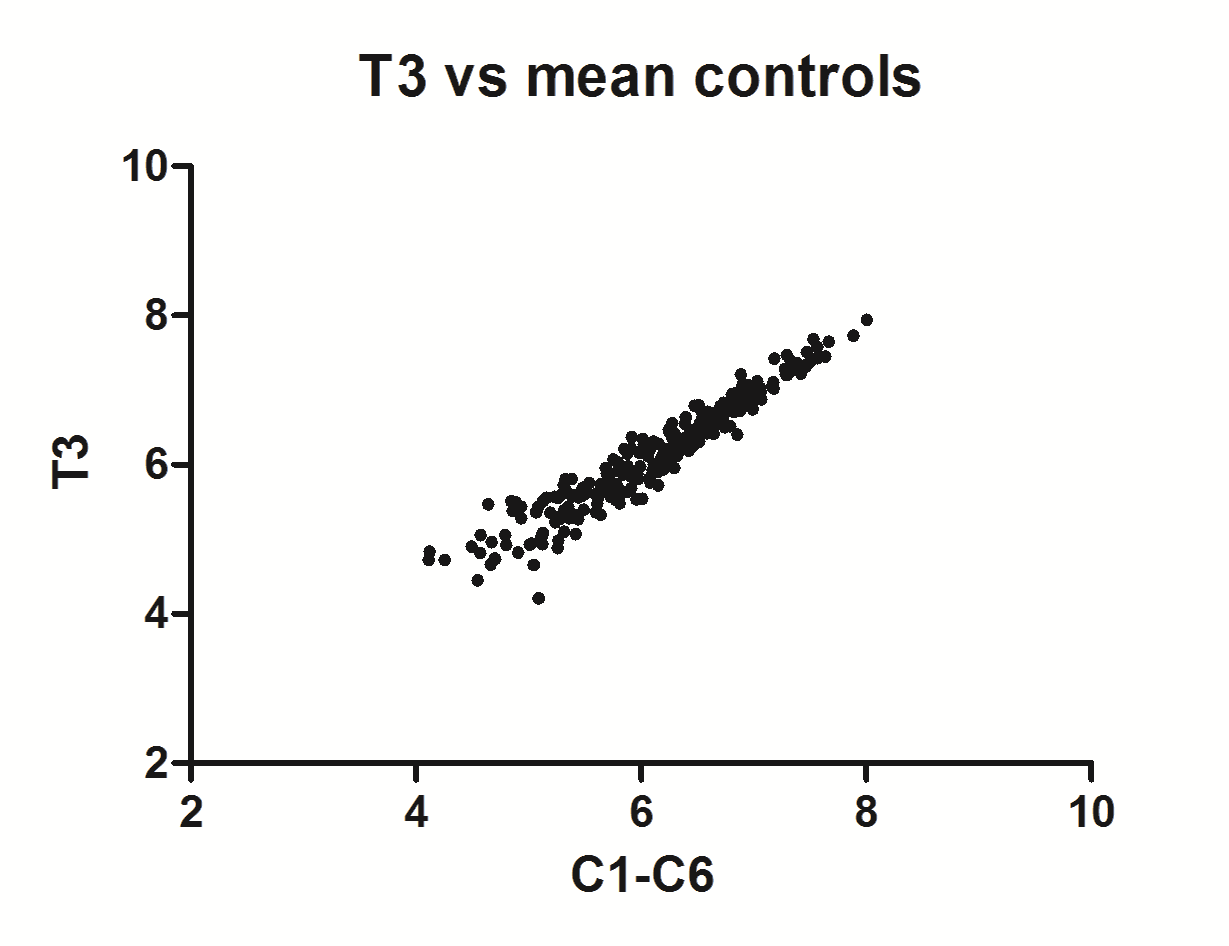**  r^2^=0.92 | **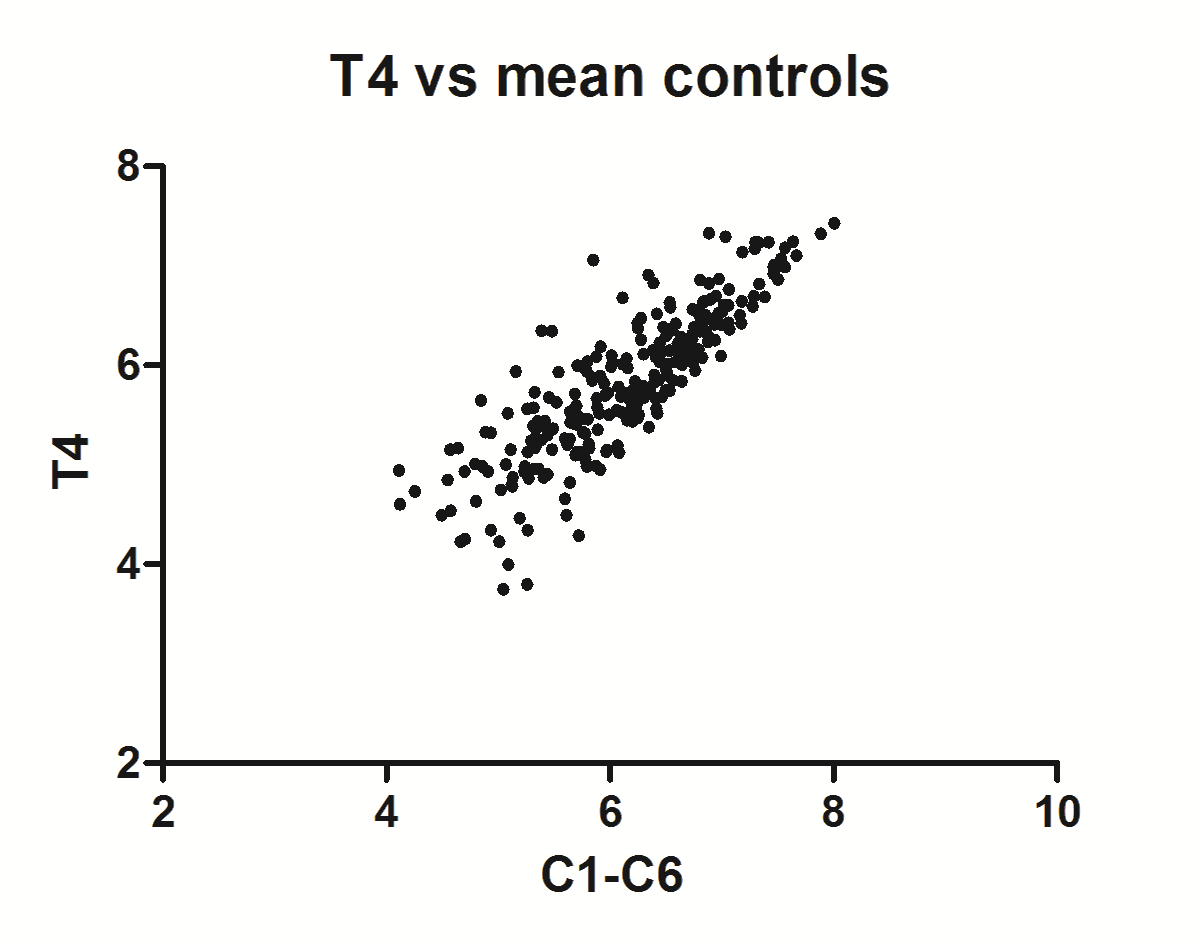**  r^2^=0.72 |
| **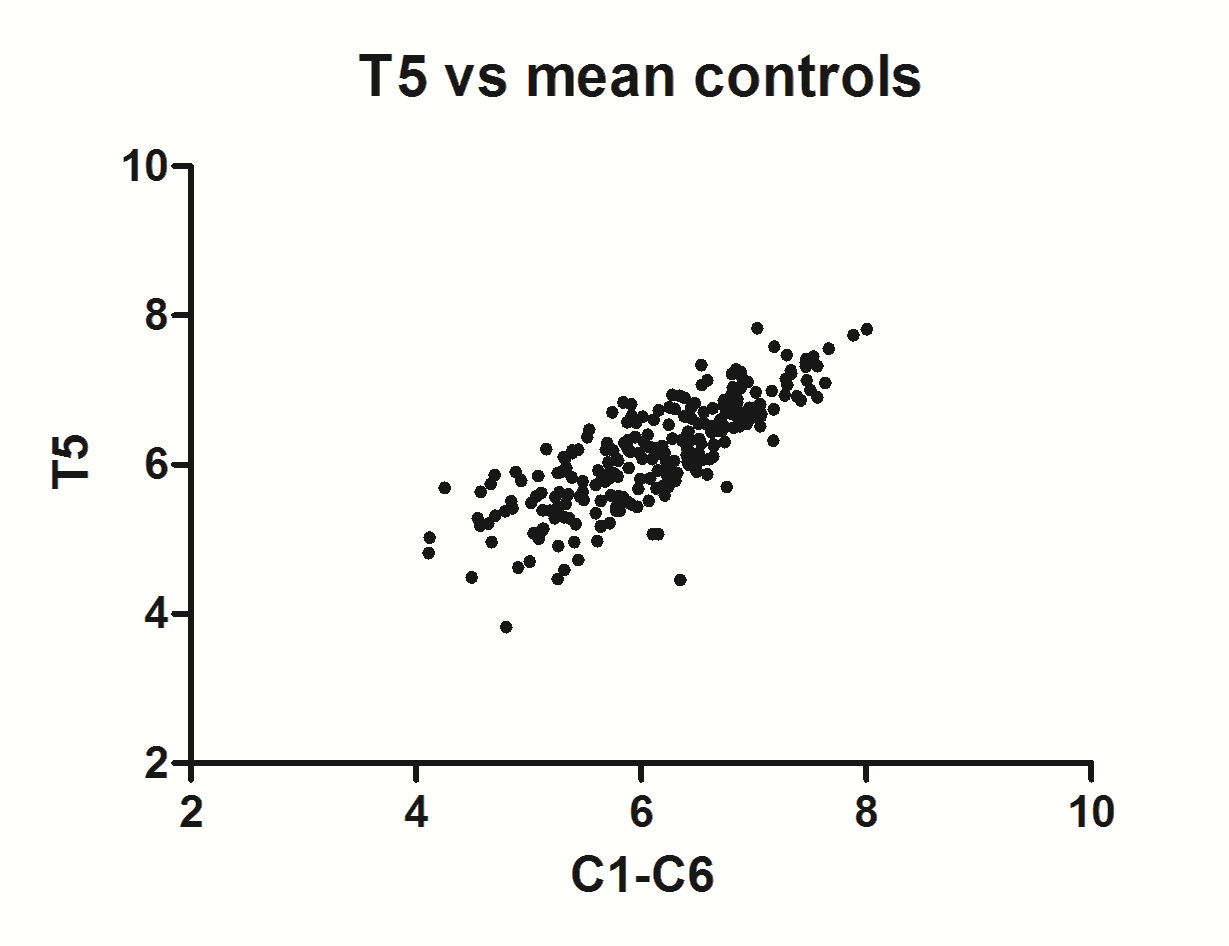**  r^2^=0.64 | **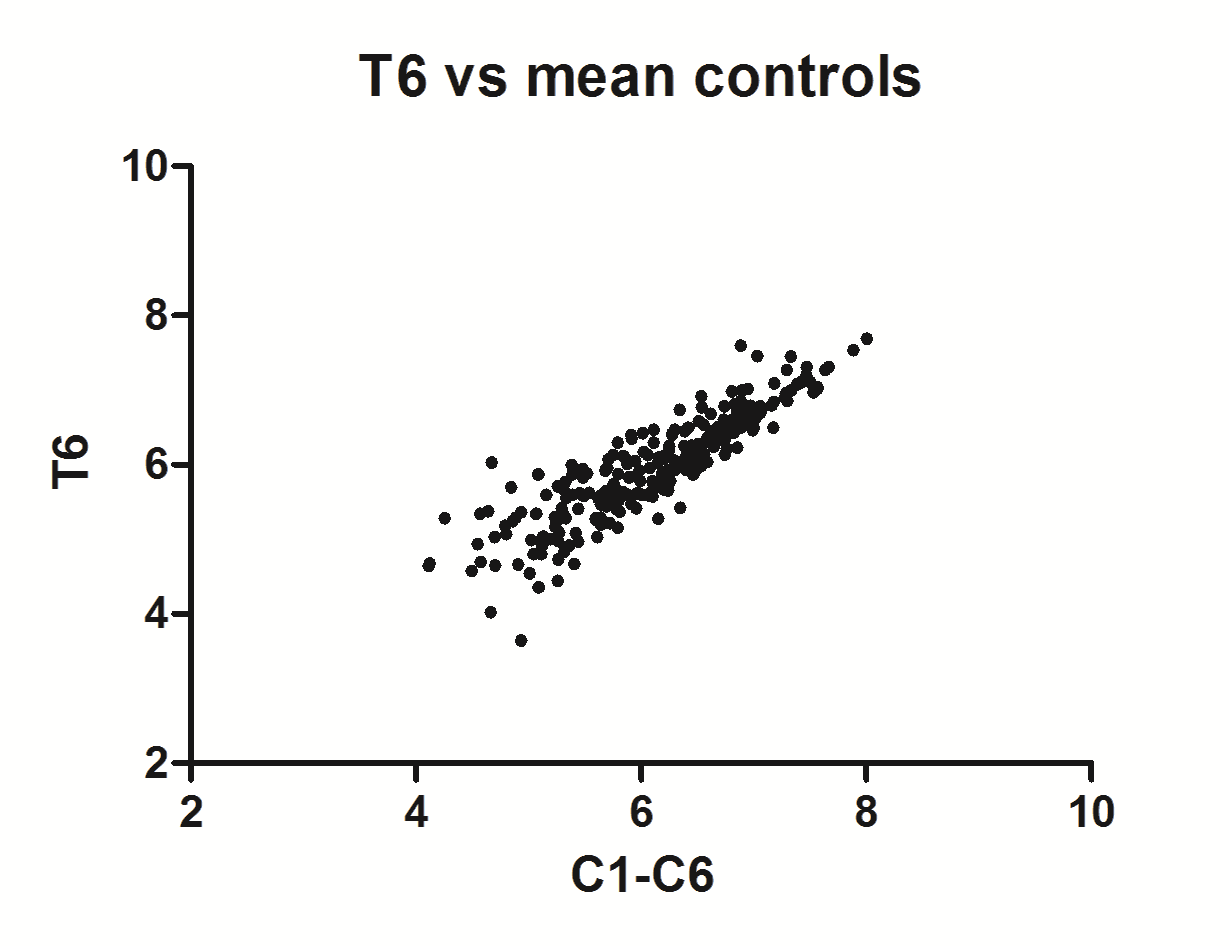**  r^2^=0.79 |

**D**. Electron Microscopy Samples

|  | **Sample ID** | **Age** | **Sex** | **Patient pathology** |
| --- | --- | --- | --- | --- |
| PERITUMOURAL CONTROL  Cohort | 13^p^ | 69 | M | GBM |
|  | 14^p^ | 19 | F | GBM |
|  | 15^p^ | 56 | F | GBM |
|  | 16^p^ | 39 | F | GBM |
|  | 17 | 35 | M | GBM |
|  | 18 | 48 | M | GBM |
|  | 19 | 27 | F | GBM |
| GBM  Cohort | 20^p^ | 69 | M | GBM |
|  | 21^p^ | 19 | F | GBM |
|  | 22 | 57 | F | GBM |
|  | 23 | 40 | F | GBM |
|  | 24 | 45 | F | GBM |
|  | 25^p^ | 56 | F | GBM |
|  | 26^p^ | 39 | F | GBM |

(^P^ paired GBM and peritumoural control samples)
