# Supplementary material for: Interactions among mitochondrial proteins altered in glioblastoma
Source: J Neurooncol. 2014 Apr 13;118(2):247–56. doi: 10.1007/s11060-014-1430-5 (PMC4048470; doi:10.1007/s11060-014-1430-5)
Supplement: Supplementary file 2 — S2: Data for all proteins in the enriched mitochondrial fractions identified by ≥2 peptides by LC–MS. Proteins are listed by category: 1) mitochondrial proteins significantly increased in GBM (p ≤ 0.05, ≥2-fold change), 2) mitochondrial proteins significantly decreased in GBM (p ≤ 0.05, ≥2-fold change), 3) non-mitochondrial proteins significantly increased in GBM (p ≤ 0.05, ≥2-fold change), 4) non-mitochondrial proteins significantly decreased in GBM (p ≤ 0.05, ≥2-fold change), 5) all other proteins identified in GBM (no significant change). The protein accession number (IPI), gene name, p-value, magnitude of protein response, the number of peptides for protein identification and the Mascot identification score are listed for each protein. The raw data are available on the public data repository PRIDE (see methods). Supplementary material 2 (DOC 1004 kb) [file 11060_2014_1430_MOESM2_ESM.doc]

**Supplementary Information 2**

| **Protein ID** | **Gene Name** | **Protein Name** | **p-value^a^** | **Fold change^b^** | **#**  **Pep ^c^** | **Score ^d^** |
| --- | --- | --- | --- | --- | --- | --- |
| **INCREASED in GBM – MITOCHONDRIAL PROTEINS** | | | | | | |
| IPI00465436 | CAT | Catalase | 1.45E-07 | 6.1 | 3 | 97 |
| IPI00002520 | SHMT2 | Serine hydroxymethyltransferase | 3.48E-06 | 5.5 | 2 | 82 |
| IPI00419237 | LAP3 | Isoform1 of aminopeptidase | 0.0001 | 5.1 | 7 | 347 |
| IPI00026105 | SCP2 | Isoform SCPx of Non-specific lipid-transfer protein | 0.0002 | 5.5 | 2 | 62 |
| IPI00215901 | AK2 | Adenylate kinase 2 | 0.0002 | 5.3 | 4 | 223 |
| IPI00017726 | HSD17B10 | Isoform 1 of 3-hydroxyacyl-CoA dehydrogenase type2 | 0.0003 | 3.4 | 7 | 538 |
| IPI00032103 | GATM | Glycine amidinotransferase | 0.0006 | 6.0 | 9 | 595 |
| IPI00096066 | SUCLG2 | Succinyl-CoA ligase [GDP-forming] subunit beta | 0.0008 | 10 | 4 | 167 |
| IPI00033217 | AASS | Alpha-aminoadipic semialdehyde synthase | 0.0012 | 4.5 | 4 | 184 |
| IPI00019906 | BSG | Isoform 2 of Basigin | 0.0014 | 2.1 | 3 | 137 |
| IPI00026958 | FDXR | NADPH:adrenodoxin oxidoreductase | 0.0015 | 3.2 | 3 | 107 |
| IPI00306748 | ABCB7 | ATP-binding cassette sub-family B member | 0.0016 | 2.2 | 2 | 133 |
| IPI00011201 | ME2 | NAD-dependent malic enzyme | 0.0023 | 2.2 | 4 | 309 |
| IPI00218342 | MTHFD1 | C-1-tetrahydrofolate synthase | 0.0024 | 8.3 | 2 | 102 |
| IPI00291262 | CLU | Isoform1 of Clusterin | 0.0027 | 5.7 | 8 | 545 |
| IPI00022314 | SOD2 | Superoxide dismutase [Mn] | 0.0029 | 4.5 | 16 | 1408 |
| IPI00910602 | NEFH | Isoform1 of Neurofilament heavy polypeptide | 0.0032 | 3.5 | 9 | 377 |
| IPI00000874 | PRDX1 | Peroxiredoxin-1 | 0.0034 | 2.1 | 13 | 664 |
| IPI00003482 | DECR1 | 2,4-dienoyl-CoA reductas | 0.0045 | 2.4 | 9 | 497 |
| IPI00001960 | CLIC4 | Chloride intracellular channel protein 4 | 0.0048 | 6.3 | 10 | 500 |
| IPI00005040 | ACADM | Medium-chain specific acyl-CoA dehydrogenase | 0.0049 | 4.5 | 6 | 219 |
| IPI00011937 | PRDX4 | Peroxiredoxin-4 | 0.0052 | 2.9 | 5 | 279 |
| IPI00300567 | DCI | Isoform 1 of 3,2-trans-enoyl-CoA isomerase | 0.0052 | 2.8 | 2 | 122 |
| IPI00328156 | MAOB | Amine oxidase [flavin-containing] B | 0.0059 | 2.6 | 22 | 1351 |
| IPI00916939 | HIBCH | 3-hydroxyisobutyryl-CoA hydrolase | 0.0082 | 2.9 | 4 | 127 |
| IPI00554617 | TPP1 | Tripeptidyl-peptidase 1 | 0.0085 | 3.4 | 5 | 337 |
| IPI00295741 | CTSB | Cathepsin B | 0.0086 | 2.2 | 4 | 319 |
| IPI00168603 | CHDH | Choline dehydrogenase | 0.0107 | 3.5 | 6 | 272 |
| IPI00843789 | GLDC | Glycine dehydrogenase [decarboxylating] | 0.0116 | 4.7 | 2 | 59 |
| IPI00294159 | SLC25A1 | Tricarboxylate transport protein | 0.0271 | 2.4 | 3 | 107 |
| IPI00024580 | MCCC1 | Methylcrotonoyl-CoA carboxylase subunit alpha | 0.0271 | 3.8 | 2 | 108 |
| IPI00021805 | MGST1 | Microsomal glutathione S-transferase 1 | 0.0289 | 2.4 | 2 | 104 |
| IPI00299402 | PC | Pyruvate carboxylase | 0.0290 | 2.1 | 11 | 435 |
| IPI00216293 | TST | Thiosulfate sulfurtransferase | 0.0319 | 4.2 | 3 | 169 |
| IPI00008483 | MAOA | Amine oxidase [flavin-containing] A | 0.0323 | 2.4 | 16 | 786 |
| IPI00927606 | GPX1 | glutathione peroxidase 1 isoform 1 | 0.0342 | 2.3 | 4 | 170 |
| IPI00002245 | ACSS3 | Isoform1 of Acyl-CoA synthetase family member 3 | 0.0377 | 2.6 | 2 | 65 |
| IPI00016568 | AK3L1 | Adenylate kinase isoenzyme 4 | 0.0459 | 2.7 | 5 | 305 |
| IPI00001539 | ACAA2 | 3-ketoacyl-CoA thiolase | 0.0478 | 3.1 | 9 | 483 |
| **DECREASED in GBM – MITOCHONDRIAL PROTEINS** | | | | | | |
| IPI00032904 | SNCB | Beta-synuclein | 8.81E-05 | 0.2 | 4 | 355 |
| IPI00333763 | GLRX5 | Glutaredoxin-related protein 5 | 0.0005 | 0.3 | 2 | 64 |
| IPI00018246 | HK1 | Hexokinase-1 | 0.0014 | 0.4 | 48 | 3168 |
| IPI00304814 | GPX4 | Phospholipid hydroperoxide glutathione peroxidase | 0.0016 | 0.4 | 3 | 84 |
| IPI00026516 | OXCT1 | Succinyl-CoA:3-ketoacid-coenzyme A transferase 1 | 0.0018 | 0.4 | 21 | 1684 |
| IPI00658109 | CKMT1B | Creatine kinase, ubiquitous | 0.0019 | 0.3 | 23 | 1698 |
| IPI00017802 | AUH | Methylglutaconyl-CoA hydratase | 0.0021 | 0.1 | 2 | 50 |
| IPI00217232 | SUCLA2 | Isoform2 of Succinyl-CoA ligase [ADP-forming] β | 0.0025 | 0.3 | 9 | 357 |
| IPI00010415 | ACOT7 | Isoform1 of Cytosolic acyl co-A thioester hydrolase | 0.0026 | 0.4 | 3 | 113 |
| IPI00003970 | ME3 | NADP-dependent malic enzyme | 0.0030 | 0.3 | 9 | 466 |
| IPI00011770 | NDUFA4 | NADH dehydrogenase [ubiquinone] 1 alpha, subunit 4 | 0.0035 | 0.4 | 3 | 78 |
| IPI00289159 | GLS | Isoform KGA of Glutaminase kidney isoform | 0.0038 | 0.3 | 22 | 1484 |
| IPI00216085 | COX6B1 | Cytochrome c oxidase subunit 6B1 | 0.0039 | 0.3 | 7 | 434 |
| IPI00386271 | SLC25A12 | Calcium-binding mitochondrial carrier protein Aralar1 | 0.0039 | 0.3 | 22 | 1460 |
| IPI00020510 | CISD1 | CDGSH iron sulfur domain-containing protein 1 | 0.0041 | 0.3 | 6 | 272 |
| IPI00479905 | NDUFB10 | NADH dehydrogenase [ubiquinone] 1 beta, subunit 10 | 0.0042 | 0.4 | 6 | 303 |
| IPI00029558 | NDUFC2 | NADH dehydrogenase [ubiquinone] 1 subunit C2 | 0.0048 | 0.3 | 2 | 45 |
| IPI00003856 | ATP6V1E1 | V-type proton ATPase subunit E 1 | 0.0049 | 0.4 | 5 | 278 |
| IPI00025086 | COX5A | Cytochrome c oxidase subunit 5A | 0.0060 | 0.4 | 8 | 475 |
| IPI00084828 | STXBP1 | Isoform 1 of Syntaxin-binding protein 1 | 0.0061 | 0.4 | 39 | 2872 |
| IPI00015141 | CKMT2 | Creatine kinase, sarcomeric | 0.0061 | 0.2 | 3 | 192 |
| IPI00007682 | ATP6V1A | V-type proton ATPase catalytic subunit A | 0.0061 | 0.3 | 14 | 953 |
| IPI00024920 | ATP5D | ATP synthase subunit delta | 0.0068 | 0.4 | 3 | 192 |
| IPI00003925 | PDHB | Pyruvate dehydrogenase E1 component subunit beta | 0.0068 | 0.4 | 11 | 644 |
| IPI00006579 | COX4l1 | Cytochrome c oxidase subunit 4 isoform 1 | 0.0073 | 0.4 | 12 | 526 |
| IPI00018206 | GOT2 | Aspartate aminotransferase | 0.0076 | 0.4 | 24 | 1549 |
| IPI00028883 | NDUFB8 | NADH dehydrogenase [ubiquinone] 1 beta, subunit 8 | 0.0078 | 0.3 | 5 | 209 |
| IPI00642807 | COX6C | Cytochrome c oxidase subunit 6C | 0.0081 | 0.4 | 7 | 332 |
| IPI00015972 | NDUFA12 | NADH dehydrogenase [ubiquinone] 1 alpha subunit 12 | 0.0082 | 0.4 | 7 | 488 |
| IPI00005966 | DPYSL2 | Dihydropyrimidinase-related protein 2 | 0.0083 | 0.4 | 30 | 2358 |
| IPI00257508 | NDUFS7 | NADH dehydrogenase [ubiquinone] iron-sulfur 7 | 0.0088 | 0.4 | 3 | 114 |
| IPI00307749 | SLC25A11 | Mitochondrial 2-oxoglutarate/malate carrier protein | 0.0098 | 0.4 | 13 | 702 |
| IPI00219729 | IDH3A | Isoform 1 of Isocitrate dehydrogenase [NAD] subunit α | 0.0098 | 0.4 | 16 | 1075 |
| IPI00030702 | NIPSNAP1 | Protein NipSnap homolog 1 | 0.0099 | 0.5 | 9 | 578 |
| IPI00304435 | COX7A2 | Cytochrome c oxidase polypeptide 7A2 | 0.0104 | 0.5 | 3 | 249 |
| IPI00026570 | ATP5H | Isoform 1 of ATP synthase subunit d | 0.0109 | 0.5 | 14 | 679 |
| IPI00220487 | OGDHL | 2-oxoglutarate dehydrogenase E1 component-like | 0.0110 | 0.3 | 15 | 710 |
| IPI00643720 | COX5B | Cytochrome c oxidase subunit 5B | 0.0117 | 0.4 | 7 | 293 |
| IPI00021785 | AP2M1 | Isoform 1 of AP-2 complex subunit mu-1 | 0.0119 | 0.5 | 9 | 364 |
| IPI00022256 | NDUFA11 | NADH dehydrogenase [ubiquinone] 1 alpha subunit 11 | 0.0119 | 0.4 | 5 | 423 |
| IPI00329301 | NDUFV2 | NADH dehydrogenase [ubiquinone] flavoprotein | 0.0124 | 0.3 | 5 | 223 |
| IPI00291328 | SLC25A22 | Mitochondrial glutamate carrier 1 | 0.0128 | 0.3 | 8 | 606 |
| IPI00003004 | NDUFS5 | NADH dehydrogenase [ubiquinone] iron-sulfur 5 | 0.0132 | 0.3 | 3 | 120 |
| IPI00220063 | PCCA | highly similar to Propionyl-CoA carboxylase α-chain | 0.0133 | 0.4 | 5 | 189 |
| IPI00552419 | ACO2 | Aconitase | 0.0141 | 0.5 | 18 | 1078 |
| IPI00382844 | COX7A2L | Cytochrome c oxidase subunit 7A-related protein | 0.0141 | 0.5 | 5 | 236 |
| IPI00022421 | NDUFA9 | NADH dehydrogenase [ubiquinone] 1 alpha subunit 9 | 0.0153 | 0.4 | 15 | 797 |
| IPI00003968 | ATPlF1 | Putative uncharacterized protein DKFZp564G0422 | 0.0159 | 0.4 | 2 | 84 |
| IPI00553153 | VDAC2 | Voltage-dep. anion-selective channel protein 2 | 0.0160 | 0.5 | 15 | 1037 |
| IPI00024145 | VDAC3 | Voltage-dep. anion-selective channel protein3 | 0.0168 | 0.4 | 10 | 650 |
| IPI00031804 | COX6A1P2 | Cytochrome c oxidase subunit 6A1 | 0.0170 | 0.4 | 2 | 94 |
| IPI00021793 | NDUFS6 | NADH dehydrogenase iron-sulfur protein6 | 0.0177 | 0.4 | 6 | 274 |
| IPI00025344 | ATP5A1 | ATP synthase subunit alpha | 0.0179 | 0.5 | 47 | 2843 |
| IPI00440493 | UQCRFS1 | Cytochrome b-c1 complex subunit Rieske | 0.0179 | 0.5 | 7 | 366 |
| IPI00026964 | NDUFA7 | NADH dehydrogenase [ubiquinone] 1 alpha, subunit 7 | 0.0182 | 0.4 | 7 | 370 |
| IPI00452731 | NDUFA10 | NADH dehydrogenase [ubiquinone] 1 alpha, subunit10 | 0.0185 | 0.5 | 9 | 445 |
| IPI00029561 | NDUFS1 | NADH-ubiquinone oxidoreductase 75 kDa subunit | 0.0185 | 0.4 | 32 | 2019 |
| IPI00604664 | IDH3G | Isocitrate dehydrogenase [NAD] subunit gamma | 0.0189 | 0.4 | 8 | 504 |
| IPI00220150 | NDUFV1 | NADH dehydrogenase [ubiquinone] flavoprotein 1 | 0.0197 | 0.4 | 19 | 1265 |
| IPI00028520 | NDUFA2 | NADH dehydrogenase [ubiquinone] 1 alpha, subunit 2 | 0.0200 | 0.3 | 4 | 212 |
| IPI00219381 | NDUFA3 | NADH dehydrogenase [ubiquinone] 1 alpha, subunit 3 | 0.0206 | 0.3 | 2 | 69 |
| IPI00028881 | DLAT | Dihydrolipoyllysine-residue acetyltransferase | 0.0210 | 0.4 | 13 | 589 |
| IPI00021338 | NDUFS4 | NADH dehydrogenase [ubiquinone] iron-sulfur protein | 0.0218 | 0.4 | 4 | 184 |
| IPI00011217 | ACO2 | Aconitate hydratase | 0.0221 | 0.5 | 39 | 2817 |
| IPI00017855 | NDUFS8 | NADH dehydrogenase [ubiquinone] iron-sulfur 8 | 0.0226 | 0.4 | 4 | 240 |
| IPI00010845 | NDUFS2 | NADH dehydrogenase [ubiquinone] iron-sulfur 2 | 0.0234 | 0.4 | 12 | 623 |
| IPI00025239 | CLTC | Isoform 1 of Clathrin heavy chain 1 | 0.0278 | 0.5 | 64 | 4334 |
| IPI00024067 | ATP5O | ATP synthase subunit O | 0.0287 | 0.4 | 13 | 984 |
| IPI00007611 | NDUFB3 | NADH dehydrogenase [ubiquinone] 1 beta, subunit 3 | 0.0290 | 0.4 | 2 | 55 |
| IPI00219383 | ATP5J | ATP synthase-coupling factor 6 | 0.0296 | 0.5 | 6 | 488 |
| IPI00002521 | ATP5B | ATP synthase subunit beta | 0.0313 | 0.5 | 35 | 3242 |
| IPI00303476 | IDH3B | Isocitrate dehydrogenase 3, beta subunit isoform | 0.0332 | 0.4 | 7 | 446 |
| IPI00304417 | MTX2 | Metaxin-2 | 0.0336 | 0.5 | 5 | 218 |
| IPI00025717 | NDUFS3 | NADH dehydrogenase [ubiquinone] iron-sulfur 3 | 0.0355 | 0.5 | 11 | 815 |
| IPI00025796 | NDUFB5 | NADH dehydrogenase [ubiquinone] 1 beta, subunit 5 | 0.0411 | 0.4 | 2 | 111 |
| IPI00013459 | DNAJC5 | Isoform 1 of DnaJ homolog subfamily C member 5 | 0.0421 | 0.3 | 5 | 261 |
| IPI00402231 | BDH1 | D-beta-hydroxybutyrate dehydrogenase | 0.0457 | 0.4 | 8 | 280 |
| IPI00025341 | HK1 | Hexokinase-1 | 0.0014 | 0.4 | 48 | 3168 |
| **INCREASED in GBM – NON-MITOCHONDRIAL (GO Term 0005739) PROTEINS** | | | | | | |
| IPI00472169 | HLA-DRB4 | HLA class II histocompatibility antigen DRB1-15 β | 2.89E-07 | 9.6 | 3 | 128 |
| IPI00473006 | HLA-C | HLA class I histocompatibility antigen Cw-17 α-chain | 5.45E-07 | 50.4 | 2 | 186 |
| IPI00876838 | HLA-DRB4 | MHC class II HLA beta chain (Fragment) | 7.00E-07 | 8.4 | 4 | 133 |
| IPI00004656 | B2M | Beta-2-microglobulin | 8.73E-07 | 9.1 | 2 | 53 |
| IPI00789155 | CALU | clone highly similar to Calumenin | 1.84E-06 | 9.6 | 5 | 263 |
| IPI00020984 | CANX | cDNA FLJ55574, highly similar to Calnexin | 5.80E-06 | 3.4 | 16 | 996 |
| IPI00009342 | IQGAP1 | Ras GTPase-activating-like protein IQGAP1 | 1.13E-05 | 7.1 | 8 | 384 |
| IPI00010397 | HLA-DRB4 | HLA class II DR-beta | 1.14E-05 | 10.6 | 3 | 78 |
| IPI00299571 | PDIA6 | Isoform 2 of Protein disulfide-isomerase A6 | 1.15E-05 | 5.0 | 10 | 602 |
| IPI00329801 | ANXA5 | Annexin A5 | 1.58E-05 | 6.9 | 17 | 1180 |
| IPI00026569 | HLA-A | HLA class I histocompatibility antigen, A-1 alpha chain | 1.77E-05 | 11.9 | 3 | 229 |
| IPI00396485 | EEF1A1 | Elongation factor 1-alpha 1 | 2.61E-05 | 5.7 | 17 | 1052 |
| IPI00221091 | RPS15A | 40S ribosomal protein S15a | 2.63E-05 | 9.3 | 2 | 67 |
| IPI00297084 | DDOST | Dolichyl-diphosphooligosaccharide-protein | 2.86E-05 | 3.6 | 2 | 97 |
| IPI00472855 | HLA-A | HLA class I histocompatibility antigen, A-30 alpha chain | 3.00E-05 | 12.0 | 4 | 277 |
| IPI00219757 | GSTP1 | Glutathione S-transferase P | 3.09E-05 | 3.2 | 3 | 180 |
| IPI00471955 | HLA-B | HLA class I histocompatibility antigen, B-50 alpha chain | 3.17E-05 | 14.5 | 2 | 167 |
| IPI00006752 | MAP4K4 | Mitogen-activated protein kinase kinase kinase kinase 4 | 3.26E-05 | 2.7 | 2 | 52 |
| IPI00025512 | HSPB1 | Heat shock protein beta-1 | 3.48E-05 | 6.7 | 10 | 547 |
| IPI00909453 | HSPB1 | cDNA FLJ52243, highly similar to Heat-shock protein β1 | 3.68E-05 | 7.0 | 9 | 393 |
| IPI00297160 | CD44 | Isoform 12 of CD44 antigen | 3.70E-05 | 6.1 | 7 | 451 |
| IPI00647457 | HLA-A | Major histocompatibility complex, class I, A | 3.87E-05 | 6.2 | 4 | 253 |
| IPI00025091 | RPS11 | 40S ribosomal protein S11 | 4.55E-05 | 6.9 | 2 | 113 |
| IPI00872984 | HLA-DRB4 | Putative uncharacterized protein | 4.82E-05 | 7.3 | 3 | 100 |
| IPI00554723 | RPL10 | 60S ribosomal protein L10 | 4.83E-05 | 8.4 | 3 | 105 |
| IPI00008274 | CAP1 | Isoform 1 of Adenylyl cyclase-associated protein 1 | 5.22E-05 | 3.2 | 8 | 386 |
| IPI00025363 | GFAP | Isoform 1 of Glial fibrillary acidic protein | 5.92E-05 | 5.5 | 50 | 3545 |
| IPI00646304 | PPIB | PPIB Peptidyl-prolyl cis-trans isomerase B | 6.21E-05 | 3.9 | 10 | 657 |
| IPI00216691 | PFN1 | Profilin-1 | 6.69E-05 | 2.9 | 6 | 253 |
| IPI00026154 | PRKCSH | highly similar to Glucosidase 2 subunitβ | 7.00E-05 | 3.5 | 4 | 197 |
| IPI00186290 | EEF2 | Elongation factor 2 | 7.18E-05 | 4.6 | 13 | 767 |
| IPI00550523 | ATL3 | Isoform 1 of Atlastin-3 | 7.57E-05 | 6.5 | 2 | 47 |
| IPI00025252 | PDIA3 | Protein disulfide-isomerase A3 | 8.57E-05 | 3.1 | 22 | 1096 |
| IPI00465070 | HIST1H3D | Histone H3.1 | 8.86E-05 | 17.7 | 5 | 460 |
| IPI00219365 | MSN | Moesin | 8.87E-05 | 6.5 | 21 | 1173 |
| IPI00010796 | P4HB | Protein disulfide-isomerase | 0.0001 | 3.4 | 20 | 996 |
| IPI00215983 | CA1 | Carbonic anhydrase 1 | 0.0001 | 5.8 | 10 | 568 |
| IPI00219219 | LGALS1 | Galectin-1 | 0.0001 | 6.8 | 4 | 397 |
| IPI00012828 | ACAA1 | 3-ketoacyl-CoA thiolase, peroxisomal | 0.0001 | 3.9 | 2 | 151 |
| IPI00020599 | CALR | Calreticulin | 0.0002 | 4.0 | 17 | 1017 |
| IPI00395887 | TMX1 | Thioredoxin-related transmembrane protein 1 | 0.0002 | 7.5 | 2 | 133 |
| IPI00217030 | RPS4X | 40S ribosomal protein S4, X isoform | 0.0002 | 8.0 | 2 | 69 |
| IPI00009904 | PDIA4 | Protein disulfide-isomerase A4 | 0.0002 | 5.1 | 4 | 154 |
| IPI00014177 | sept2 | Septin-2 | 0.0002 | 2.6 | 12 | 737 |
| IPI00023283 | TTN | Isoform 2 of Titin | 0.0002 | 4.1 | 3 | 67 |
| IPI00219525 | PGD | 6-phosphogluconate dehydrogenase, decarboxylating | 0.0002 | 3.3 | 2 | 103 |
| IPI00014958 | PON2 | Isoform 1 of Serum paraoxonase/arylesterase 2 | 0.0002 | 5.8 | 6 | 308 |
| IPI00171611 | HIST2H3C | Histone H3.2 | 0.0002 | 14.0 | 4 | 405 |
| IPI00219910 | - | 22 kDa protein | 0.0002 | 7.2 | 5 | 325 |
| IPI00843975 | EZR | Ezrin | 0.0002 | 3.8 | 16 | 767 |
| IPI00298860 | LTF | cDNA FLJ78440, highly similar to Human lactoferrin | 0.0002 | 6.8 | 3 | 130 |
| IPI00141318 | CKAP4 | Isoform 1 of Cytoskeleton-associated protein 4 | 0.0002 | 3.6 | 6 | 342 |
| IPI00003362 | HSPA5 | HSPA5 protein | 0.0002 | 2.9 | 29 | 1939 |
| IPI00007676 | HSD17B12 | Estradiol 17-beta-dehydrogenase 12 | 0.0002 | 3.0 | 2 | 99 |
| IPI00008529 | RPLP2 | 60S acidic ribosomal protein P2 | 0.0002 | 3.3 | 2 | 133 |
| IPI00376005 | EIF5A | Isoform 2 Eukaryotic translation initiation factor 5A-1 | 0.0003 | 3.6 | 7 | 326 |
| IPI00383751 | - | Calreticulin (Fragment) | 0.0003 | 3.4 | 5 | 284 |
| IPI00028635 | RPN2 | Dolichyl-diphosphooligosaccharide-protein 2 | 0.0003 | 4.4 | 5 | 276 |
| IPI00550363 | TAGLN2 | Transgelin-2 | 0.0003 | 5.8 | 6 | 349 |
| IPI00017367 | RDX | Radixin, isoform CRA_a | 0.0003 | 4.0 | 14 | 590 |
| IPI00027230 | HSP90B1 | Endoplasmin | 0.0003 | 2.4 | 21 | 1072 |
| IPI00298994 | TLN1 | Talin-1 | 0.0004 | 5.4 | 8 | 464 |
| IPI00479877 | ALDH9A1 | aldehyde dehydrogenase 9A1 | 0.0004 | 2.2 | 7 | 267 |
| IPI00010182 | DBI | Isoform 1 of Acyl-CoA-binding protein | 0.0004 | 2.8 | 5 | 341 |
| IPI00383581 | GANAB | Clone highly similar to Neutral α-glucosidase AB | 0.0004 | 3.0 | 17 | 805 |
| IPI00219156 | RPL30 | 60S ribosomal protein L30 | 0.0005 | 3.2 | 2 | 129 |
| IPI00329389 | RPL6 | 60S ribosomal protein L6 | 0.0005 | 4.2 | 3 | 242 |
| IPI00000875 | EEF1G | highly similar to Elongation factor 1-gamma | 0.0005 | 4.7 | 5 | 245 |
| IPI00026272 | HIST1H2AG | Histone H2A type 1-B/E | 0.0006 | 18.1 | 7 | 413 |
| IPI00019502 | MYH9 | Isoform 1 of Myosin-9 | 0.0006 | 5.9 | 30 | 1847 |
| IPI00018873 | NAMPT | Isoform 1 of Nicotinamide phosphoribosyltransferase | 0.0006 | 6.1 | 3 | 232 |
| IPI00032140 | SERPINH1 | Serpin H1 | 0.0006 | 22.1 | 8 | 388 |
| IPI00027341 | CAPG | Macrophage-capping protein | 0.0006 | 6.4 | 3 | 121 |
| IPI00216730 | HIST2H2AB | Histone H2A type 2-B | 0.0006 | 14.3 | 6 | 274 |
| IPI00216049 | HNRNPK | Isoform 1 Heterogeneous nuclear ribonucleoprotein K | 0.0006 | 17.3 | 4 | 273 |
| IPI00335168 | MYL6 | Isoform Non-muscle of Myosin light polypeptide 6 | 0.0006 | 4.9 | 6 | 456 |
| IPI00219037 | H2AFX | Histone H2A.x | 0.0007 | 17.3 | 7 | 344 |
| IPI00003935 | HIST2H2BE | Histone H2B type 2-E | 0.0007 | 9.8 | 4 | 254 |
| IPI00028120 | EPB42 | Isoform long Erythrocyte membrane protein band 4.2 | 0.0007 | 5.6 | 5 | 173 |
| IPI00025874 | RPN1 | Dolichyl-diphosphooligosaccharide-protein | 0.0007 | 4.6 | 3 | 83 |
| IPI00216457 | HIST2H2A4 | Histone H2A type 2-A | 0.0007 | 17.3 | 7 | 513 |
| IPI00081836 | HIST1H2A | Histone H2A type 1-H | 0.0007 | 17.3 | 7 | 436 |
| IPI00299301 | SYNM | Isoform 1 of Synemin | 0.0007 | 6.4 | 2 | 54 |
| IPI00384051 | PSME2 | Putative uncharacterized protein PSME2 | 0.0008 | 9.2 | 2 | 106 |
| IPI00746165 | WDR1 | Isoform 1 of WD repeat-containing protein 1 | 0.0008 | 2.8 | 3 | 119 |
| IPI00903292 | WDR1 | Isoform 3 of WD repeat-containing protein 1 | 0.0008 | 2.8 | 3 | 119 |
| IPI00026087 | BANF1 | Barrier-to-autointegration factor | 0.0009 | 6.4 | 2 | 144 |
| IPI00018278 | H2AFV | Histone H2A.V | 0.0009 | 17.9 | 4 | 244 |
| IPI00299573 | RPL7A | 60S ribosomal protein L7a | 0.0010 | 4.9 | 3 | 137 |
| IPI00014424 | EEF1A2 | Elongation factor 1-alpha 2 | 0.0010 | 3.5 | 10 | 595 |
| IPI00829896 | HBD | Hemoglobin Lepore-Baltimore (fragment) | 0.0011 | 3.2 | 10 | 834 |
| IPI00418471 | VIM | Vimentin | 0.0012 | 17.2 | 48 | 3181 |
| IPI00218918 | ANXA1 | Annexin A1 | 0.0012 | 11.4 | 18 | 1571 |
| IPI00002372 | ABCD3 | Isoform 1 ATP-binding cassette sub-family D 3 | 0.0013 | 6.2 | 2 | 79 |
| IPI00654755 | HBB | Hemoglobin subunit beta | 0.0013 | 2.9 | 20 | 1659 |
| IPI00220741 | SPTA1 | Isoform 1 of Spectrin alpha chain, erythrocyte | 0.0013 | 3.7 | 32 | 1724 |
| IPI00005721 | LOC728358 | Neutrophil defensin 1 | 0.0013 | 18.1 | 2 | 108 |
| IPI00003918 | RPL4 | 60S ribosomal protein L4 | 0.0016 | 2.3 | 3 | 131 |
| IPI00410714 | HBA1 | Hemoglobin subunit alpha | 0.0016 | 2.8 | 15 | 1308 |
| IPI00020557 | LRP1 | Prolow-density lipoprotein receptor-related protein 1 | 0.0016 | 10.5 | 2 | 90 |
| IPI00473011 | HBD | Hemoglobin subunit delta | 0.0017 | 3.1 | 11 | 770 |
| IPI00296141 | DPP7 | Dipeptidyl-peptidase 2 | 0.0018 | 4.9 | 2 | 62 |
| IPI00465279 | LOC1002941 | HLA class II histocompatibility antigen, DRB1-7 beta | 0.0019 | 7.1 | 2 | 53 |
| IPI00930351 | HBD | Hbbm fused globin protein (Fragment) | 0.0019 | 3.1 | 8 | 616 |
| IPI00550991 | SERPINA3 | highly similar to alpha-1-antichymotrypsin | 0.0020 | 5.5 | 4 | 268 |
| IPI00397526 | MYH10 | Isoform 1 of Myosin-10 | 0.0020 | 3.9 | 7 | 348 |
| IPI00028055 | TMED10 | Transmembrane emp24 domain-containing protein 10 | 0.0023 | 3.5 | 4 | 196 |
| IPI00012772 | RPL8 | 60S ribosomal protein L8 | 0.0024 | 3.8 | 4 | 142 |
| IPI00453473 | HIST1H4L | Histone H4 | 0.0026 | 16.4 | 11 | 640 |
| IPI00217465 | HIST1H1C | Histone H1.2 | 0.0026 | 19.2 | 10 | 646 |
| IPI00027463 | S100A6 | Protein S100-A6 | 0.0026 | 3.0 | 3 | 156 |
| IPI00030179 | RPL7P32 | 60S ribosomal protein L7 | 0.0026 | 2.9 | 5 | 224 |
| IPI00375676 | FTL | Ferritin light chain | 0.0027 | 2.4 | 4 | 213 |
| IPI00455315 | ANXA2 | Annexin A2 | 0.0027 | 10.5 | 20 | 1505 |
| IPI00017342 | RHOG | Rho-related GTP-binding protein RhoG | 0.0027 | 3.1 | 2 | 58 |
| IPI00217467 | HIST1H1E | Histone H1.4 | 0.0028 | 18.9 | 10 | 653 |
| IPI00031131 | C20orf3 | Isoform 1 Adipocyte plasma membrane-assoc protein | 0.0028 | 2.1 | 3 | 105 |
| IPI00011284 | COMT | Membrane-bound Catechol O-methyltransferase | 0.0029 | 2.8 | 2 | 138 |
| IPI00017596 | MAPRE1 | Microtubule-assoc protein RP/EB family member 1 | 0.0029 | 6.7 | 3 | 117 |
| IPI00179964 | PTBP1 | Isoform 1 of Polypyrimidine tract-binding protein 1 | 0.0030 | 8.2 | 3 | 181 |
| IPI00220271 | AKR1A1 | Alcohol dehydrogenase [NADP+] | 0.0030 | 3.1 | 2 | 124 |
| IPI00016539 | SCD5 | Isoform 2 of Stearoyl-CoA desaturase 5 | 0.0030 | 8.3 | 2 | 80 |
| IPI00917549 | LOC646471 | Conserved hypothetical protein | 0.0033 | 3.1 | 2 | 30 |
| IPI00302592 | FLNA | Isoform 2 of Filamin-A | 0.0035 | 10.8 | 53 | 2528 |
| IPI00419258 | HMGB1 | High mobility group protein B1 | 0.0038 | 12.9 | 8 | 432 |
| IPI00027429 | FABP7 | Fatty acid binding protein 7, brain, isoform CRA_b | 0.0038 | 5.8 | 3 | 238 |
| IPI00025491 | EIF4A1 | Eukaryotic initiation factor 4A-I | 0.0040 | 2.1 | 6 | 289 |
| IPI00002824 | CSRP2 | Cysteine and glycine-rich protein 2 | 0.0040 | 27.4 | 2 | 92 |
| IPI00013485 | RPS2 | 40S ribosomal protein S2 | 0.0041 | 3.3 | 2 | 72 |
| IPI00552276 | SNX3 | Isoform 4 of Sorting nexin-3 | 0.0041 | 4.2 | 2 | 49 |
| IPI00018274 | EGFR | Isoform 1 of Epidermal growth factor receptor | 0.0042 | 5.7 | 9 | 431 |
| IPI00215965 | HNRNPA1 | Isoform A1-B heterogeneous nuclear ribonucleoprotein | 0.0051 | 9.8 | 6 | 290 |
| IPI00893541 | PDIA3 | PDIA3 14 kDa protein | 0.0051 | 3.2 | 5 | 285 |
| IPI00033494 | MYL12B | Myosin regulatory light chain 12B | 0.0053 | 3.2 | 3 | 96 |
| IPI00018534 | HIST1H2BL | Histone H2B type 1-L | 0.0056 | 8.2 | 4 | 269 |
| IPI00291866 | SERPING1 | Plasma protease C1 inhibitor | 0.0058 | 7.9 | 2 | 85 |
| IPI00332371 | PFKL | Isoform 1 of 6-phosphofructokinase, liver type | 0.0059 | 2.3 | 4 | 172 |
| IPI00215743 | RRBP1 | Isoform 3 of Ribosome-binding protein 1 | 0.0059 | 6.6 | 2 | 57 |
| IPI00182533 | RPL28 | 60S ribosomal protein L28 | 0.0061 | 5.5 | 2 | 63 |
| IPI00419373 | HNRNPA3 | Isoform 1 Heterogeneous nuclear ribonucleoprotein A3 | 0.0065 | 23.0 | 2 | 124 |
| IPI00218414 | CA2 | Carbonic anhydrase 2 | 0.0067 | 2.2 | 11 | 591 |
| IPI00470528 | RPL15 | 60S ribosomal protein L15 | 0.0068 | 3.6 | 2 | 105 |
| IPI00022463 | TF | Serotransferrin | 0.0069 | 2.7 | 22 | 1127 |
| IPI00419802 | HIBCH | Isoform 1 of 3-hydroxyisobutyryl-CoA hydrolase | 0.0069 | 2.6 | 6 | 310 |
| IPI00219038 | LOC440926 | Histone H3.3 | 0.0070 | 4.8 | 4 | 422 |
| IPI00942498 | LOC440926 | Histone H3 (Fragment) | 0.0070 | 4.4 | 3 | 169 |
| IPI00219097 | HMGB2 | High mobility group protein B2 | 0.0074 | 10.3 | 6 | 286 |
| IPI00003443 | IFI16 | Isoform 1 Gamma-interferon-inducible protein Ifi-16 | 0.0082 | 14.0 | 4 | 175 |
| IPI00220194 | SLC2A1 | Solute carrier family2, glucose transporter-member 1 | 0.0083 | 6.8 | 4 | 182 |
| IPI00016670 | C11orf59 | UPF0404 protein C11orf59 | 0.0085 | 2.8 | 2 | 103 |
| IPI00008527 | RPLP1 | 60S acidic ribosomal protein P1 | 0.0088 | 2.2 | 2 | 137 |
| IPI00298828 | APOH | Beta-2-glycoprotein 1 | 0.0089 | 3.1 | 3 | 196 |
| IPI00465431 | LGALS3 | Galectin-3 | 0.0091 | 5.0 | 5 | 247 |
| IPI00297779 | CCT2 | T-complex protein 1 subunit beta | 0.0091 | 2.4 | 2 | 124 |
| IPI00219067 | GSTM2 | Glutathione S-transferase Mu 2 | 0.0095 | 2.8 | 2 | 80 |
| IPI00219682 | STOM | Erythrocyte band 7 integral membrane protein | 0.0102 | 3.9 | 4 | 205 |
| IPI00028564 | GBP1 | Interferon-induced guanylate-binding protein 1 | 0.0103 | 11.0 | 3 | 117 |
| IPI00183695 | S100A10 | Protein S100-A10 | 0.0111 | 10.6 | 2 | 119 |
| IPI00791534 | - | 104 kDa protein | 0.0111 | 7.0 | 21 | 1574 |
| IPI00010800 | NES | Nestin | 0.0118 | 8.2 | 9 | 394 |
| IPI00216138 | TAGLN | Transgelin | 0.0122 | 5.6 | 6 | 251 |
| IPI00216697 | ANK1 | Isoform Er1 of Ankyrin-1 | 0.0123 | 4.0 | 16 | 749 |
| IPI00219301 | MARCKS | Myristoylated alanine-rich C-kinase substrate | 0.0128 | 2.7 | 7 | 473 |
| IPI00022300 | METTL7A | Methyltransferase-like protein 7A | 0.0132 | 3.0 | 2 | 100 |
| IPI00013881 | HNRNPH1 | Heterogeneous nuclear ribonucleoprotein H | 0.0142 | 8.5 | 2 | 154 |
| IPI00178352 | FLNC | Isoform 1 of Filamin-C | 0.0144 | 5.4 | 8 | 297 |
| IPI00217766 | SCARB2 | Lysosome membrane protein 2 | 0.0147 | 2.7 | 4 | 158 |
| IPI00029745 | NFIA | Isoform 1 of Nuclear factor 1 A-type | 0.0166 | 12.5 | 2 | 74 |
| IPI00217975 | LMNB1 | Lamin-B1 | 0.0173 | 10.3 | 6 | 285 |
| IPI00217975 | LMNB1 | Lamin-B1 | 0.0173 | 10.3 | 6 | 285 |
| IPI00001639 | KPNB1 | Importin subunit beta-1 | 0.0175 | 2.4 | 5 | 174 |
| IPI00087498 | ESCO2 | N-acetyltransferase ESCO2 | 0.0178 | 4.7 | 2 | 62 |
| IPI00745872 | ALB | ALB Isoform 1 of Serum albumin | 0.0178 | 2.3 | 57 | 4471 |
| IPI00059366 | H2AFY | H2A histone family, member Y isoform 2 | 0.0186 | 8.2 | 3 | 227 |
| IPI00017704 | COTL1 | Coactosin-like protein | 0.0189 | 2.9 | 3 | 104 |
| IPI00465084 | DES | Desmin | 0.0214 | 7.4 | 8 | 298 |
| IPI00027223 | IDH1 | Isocitrate dehydrogenase [NADP] cytoplasmic | 0.0224 | 2.2 | 3 | 108 |
| IPI00386854 | HNRNPA2B | Putative uncharacterized protein HNRNPA2B1 | 0.0229 | 7.8 | 5 | 380 |
| IPI00844578 | DHX9 | ATP-dependent RNA helicase A | 0.0242 | 13.2 | 3 | 125 |
| IPI00298971 | VTN | Vitronectin | 0.0255 | 9.7 | 5 | 328 |
| IPI00028565 | GBP2 | Interferon-induced guanylate-binding protein 2 | 0.0257 | 60.6 | 2 | 78 |
| IPI00164215 | TSPYL1 | TSPYL protein | 0.0267 | 2.1 | 2 | 54 |
| IPI00215911 | APEX1 | DNA-(apurinic or apyrimidinic site) lyase | 0.0276 | 17.1 | 4 | 240 |
| IPI00306332 | RPL24 | 60S ribosomal protein L24 | 0.0289 | 2.6 | 2 | 69 |
| IPI00032825 | TMED7 | Transmembrane emp24 domain-containing protein 7 | 0.0293 | 2.3 | 2 | 83 |
| IPI00021891 | FGG | Isoform Gamma-B of Fibrinogen gamma chain | 0.0338 | 4.8 | 8 | 458 |
| IPI00000877 | HYOU1 | Hypoxia up-regulated protein 1 | 0.0361 | 2.1 | 7 | 358 |
| IPI00021885 | FGA | Isoform 1 of Fibrinogen alpha chain | 0.0375 | 4.0 | 7 | 340 |
| IPI00216682 | CNN3 | Calponin-3 | 0.0387 | 3.1 | 3 | 197 |
| IPI00013808 | ACTN4 | Alpha-actinin-4 | 0.0394 | 2.7 | 16 | 789 |
| IPI00010740 | SFPQ | Isoform long Splicing factor, proline- & glutamine-rich | 0.0397 | 7.7 | 2 | 86 |
| IPI00298497 | FGB | Fibrinogen beta chain | 0.0397 | 4.3 | 11 | 639 |
| IPI00217468 | HIST1H1B | Histone H1.5 | 0.0410 | 6.8 | 8 | 385 |
| IPI00013698 | ASAH1 | Isoform 1 of Acid ceramidase | 0.0412 | 2.1 | 3 | 141 |
| IPI00021841 | APOA1 | Apolipoprotein A-I | 0.0417 | 2.3 | 11 | 514 |
| IPI00024689 | AQP1 | Aquaporin-1 | 0.0422 | 7.3 | 3 | 379 |
| IPI00218831 | GSTM1 | Isoform 1 of Glutathione S-transferase Mu 1 | 0.0426 | 2.8 | 4 | 268 |
| IPI00002147 | CHI3L1 | Chitinase-3-like protein 1 | 0.0430 | 7.8 | 6 | 217 |
| IPI00300273 | CAMKV | Isoform 2 CaM kinase-like vesicle-associated protein | 0.0453 | 2.1 | 2 | 61 |
| IPI00007797 | FABP5L7 | Fatty acid-binding protein, epidermal | 0.0466 | 9.5 | 6 | 356 |
| IPI00027462 | S100A9 | Protein S100-A9 | 0.0467 | 2.8 | 2 | 204 |
| IPI00884105 | LAMP1 | Lysosome-associated membrane glycoprotein 1 | 0.0485 | 2.5 | 2 | 197 |
| IPI00154742 | IGLC1 | - | 0.0494 | 2.6 | 4 | 270 |
| **DECREASED in GBM – NON- MITOCHONDRIAL (GO Term 0005739) PROTEINS** | | | | | | |
| IPI00022892 | THY1 | Thy-1 membrane glycoprotein | 4.68E-05 | 4.5 | 4 | 408 |
| IPI00018311 | NPTN | Isoform 2 of Neuroplastin | 5.29E-05 | 2.8 | 2 | 111 |
| IPI00334532 | L1CAM | Isoform 2 of Neural cell adhesion molecule L1 | 5.33E-05 | 12.4 | 5 | 189 |
| IPI00010470 | SNAP25 | Isoform SNAP-25b of Synaptosomal-assoc protein 25 | 6.97E-05 | 5.0 | 11 | 801 |
| IPI00009802 | VCAN | Isoform V0 of Versican core protein | 7.25E-05 | 6.7 | 7 | 412 |
| IPI00013043 | TPPP | Tubulin polymerization-promoting protein | 8.21E-05 | 3.4 | 7 | 417 |
| IPI00306429 | MAP6 | Isoform 2 of Microtubule-associated protein 6 | 8.51E-05 | 7.5 | 4 | 180 |
| IPI00215715 | CAMK2A | Ca^2+^/calmodulin-dependent protein kinase II α 1 | 0.0001 | 4.9 | 16 | 1384 |
| IPI00220002 | PALM | Isoform 2 of Paralemmin | 0.0001 | 3.3 | 3 | 86 |
| IPI00023625 | GNG3 | Guanine nucleotide-binding protein subunit γ-3 | 0.0001 | 7.2 | 2 | 61 |
| IPI00187158 | GPM6B | glycoprotein M6B isoform 1 | 0.0002 | 4.3 | 2 | 92 |
| IPI00029184 | HAPLN2 | Hyaluronan & proteoglycan link protein 2 | 0.0002 | 27.4 | 2 | 88 |
| IPI00172450 | CAMK2G | Isoform 4 Ca^2+^/calmodulin-dep protein kinase type IIγ | 0.0002 | 4.7 | 7 | 431 |
| IPI00295601 | CEND1 | Cell cycle exit and neuronal differentiation protein 1 | 0.0002 | 5.2 | 7 | 317 |
| IPI00023504 | RAB3A | Ras-related protein Rab-3A | 0.0002 | 2.8 | 8 | 423 |
| IPI00301180 | SLC12A5 | Isoform 2 of Solute carrier family 12 member 5 | 0.0002 | 4.2 | 3 | 125 |
| IPI00027464 | PPP3R1 | Calcineurin subunit B type 1 | 0.0002 | 5.5 | 5 | 269 |
| IPI00029751 | CNTN1 | Isoform 1 of Contactin-1 | 0.0002 | 5.5 | 16 | 935 |
| IPI00018829 | SPTBN4 | Isoform 1 of Spectrin beta chain, brain 3 | 0.0002 | 2.2 | 2 | 60.6 |
| IPI00394655 | NFASC | Isoform 4 of Neurofascin | 0.0002 | 5.5 | 6 | 311 |
| IPI00074962 | ANK2 | Isoform 4 of Ankyrin-2 | 0.0003 | 3.8 | 22 | 1252 |
| IPI00011515 | PACSIN1 | Protein kinase C & casein kinase substrate protein 1 | 0.0003 | 3.4 | 11 | 471 |
| IPI00009791 | ATP2B2 | Ca^2+^ transporting ATPase2 | 0.0003 | 3.7 | 17 | 898 |
| IPI00024107 | SNCA | Isoform 1 of Alpha-synuclein | 0.0003 | 3.8 | 5 | 249 |
| IPI00553138 | VAMP2 | Vesicle-associated membrane protein 2 | 0.0003 | 4.2 | 5 | 315 |
| IPI00012441 | SYNJ1 | Synaptojanin 1 isoform a | 0.0003 | 7.1 | 2 | 52 |
| IPI00748037 | NAPB | Beta-soluble NSF attachment protein | 0.0003 | 4.1 | 11 | 696 |
| IPI00410675 | STX1B | Syntaxin-1B | 0.0004 | 4.1 | 14 | 1012 |
| IPI00216313 | VSNL1 | Visinin-like protein 1 | 0.0004 | 4.2 | 14 | 839 |
| IPI00006612 | SNAP91 | Isoform 1 of Clathrin coat assembly protein AP180 | 0.0004 | 5.8 | 9 | 512 |
| IPI00061114 | RAB3C | Ras-related protein Rab-3C | 0.0004 | 2.7 | 5 | 221 |
| IPI00220281 | GNAO1 | Isoform α -1 of Guanine nucleotide-binding protein α | 0.0004 | 4.5 | 18 | 1428 |
| IPI00021695 | ATP2B1 | IsoformD plasma membrane Ca^2+^-transport ATPase 1 | 0.0004 | 3.2 | 14 | 606 |
| IPI00019213 | DLG4 | Isoform 2 of Disks large homolog 4 | 0.0004 | 8.4 | 4 | 130 |
| IPI00398700 | GNAO1 | Isoform α-2 of Guanine nucleotide-binding protein α | 0.0004 | 4.3 | 16 | 1132 |
| IPI00028723 | GDAP1L1 | Isoform 2 Ganglioside differentiation-assoc protein 1 | 0.0004 | 4.1 | 2 | 76 |
| IPI00328128 | GNAZ | Guanine nucleotide-binding protein G(z) subunit alpha | 0.0005 | 5.8 | 2 | 50 |
| IPI00182944 | CAMK2B | Isoform 3 Ca^2+^/calmodulin-dep protein kinase type II β | 0.0005 | 4.7 | 10 | 667 |
| IPI00006451 | NSF | Vesicle-fusing ATPase | 0.0005 | 2.9 | 25 | 1462 |
| IPI00032808 | RAB3D | Ras-related protein Rab-3D | 0.0005 | 2.6 | 6 | 283 |
| IPI00299024 | BASP1 | Brain acid soluble protein 1 | 0.0006 | 5.5 | 9 | 605 |
| IPI00654569 | CAMK2D | Ca^2+^/calmodulin-dependent protein kinase type II λ | 0.0006 | 4.1 | 10 | 650 |
| IPI00033022 | DNM2 | Isoform 1 of Dynamin-2 | 0.0006 | 4.0 | 7 | 327 |
| IPI00012759 | CPLX2 | Complexin-2 | 0.0007 | 8.3 | 2 | 116 |
| IPI00019952 | GPM6A | Neuronal membrane glycoprotein M6-a | 0.0007 | 4.0 | 6 | 425 |
| IPI00014439 | QDPR | Dihydropteridine reductase | 0.0007 | 2.3 | 7 | 365 |
| IPI00219029 | GOT1 | Aspartate aminotransferase, cytoplasmic | 0.0008 | 3.7 | 10 | 323 |
| IPI00300568 | SYN1 | Isoform IA of Synapsin-1 | 0.0008 | 4.5 | 23 | 1735 |
| IPI00216171 | ENO2 | Gamma-enolase | 0.0008 | 2.7 | 18 | 1361 |
| IPI00014964 | LY6H | lymphocyte antigen 6 complex, locus H isoform b | 0.0009 | 2.5 | 2 | 66 |
| IPI00384975 | EPB41L1 | Putative uncharacterized protein DKFZp451F1711 | 0.0009 | 3.5 | 3 | 72 |
| IPI00003370 | STX1A | Isoform 1 of Syntaxin-1A | 0.0009 | 3.5 | 14 | 859 |
| IPI00003831 | ATP2B3 | XB Plasma membrane Ca^2+^-transporting ATPase 3 | 0.0010 | 2.6 | 7 | 281 |
| IPI00024062 | EPB41L1 | Isoform 1 of Band 4.1-like protein 1 | 0.0010 | 3.0 | 6 | 143 |
| IPI00005981 | TAGLN3 | Neuronal protein NP25 | 0.0011 | 4.3 | 7 | 300 |
| IPI00332887 | SIRPA | Signal-regulatory protein alpha precursor | 0.0011 | 5.2 | 8 | 453 |
| IPI00172636 | CAMK2D | Delta 6 Ca^2+/^calmodulin-dep protein kinase II λ chain | 0.0011 | 3.1 | 10 | 588 |
| IPI00013303 | LSAMP | Limbic system-associated membrane protein | 0.0012 | 4.9 | 3 | 225 |
| IPI00026268 | GNB1 | Guanine nucleotide-binding protein subunit beta-1 | 0.0012 | 2.8 | 17 | 1329 |
| IPI00011727 | SYT5 | Synaptotagmin-5 | 0.0012 | 6.9 | 2 | 54 |
| IPI00009619 | CADM3 | Isoform 2 of Cell adhesion molecule 3 | 0.0013 | 6.6 | 4 | 220 |
| IPI00001662 | OPCML | OPCML protein | 0.0015 | 4.4 | 2 | 69 |
| IPI00221332 | DNM3 | Isoform 1 of Dynamin-3 | 0.0015 | 4.4 | 7 | 493 |
| IPI00003842 | MAP2 | Isoform 1 of Microtubule-associated protein 2 | 0.0015 | 2.5 | 16 | 821 |
| IPI00001618 | RAB39 | Ras-related protein Rab-39A | 0.0017 | 2.3 | 2 | 111 |
| IPI00021475 | RAB33B | Ras-related protein Rab-33B | 0.0017 | 2.3 | 2 | 111 |
| IPI00009439 | SYT1 | Synaptotagmin-1 | 0.0018 | 3.4 | 17 | 1055 |
| IPI00000949 | CRYM | Mu-crystallin homolog | 0.0018 | 4.6 | 3 | 187 |
| IPI00032227 | RPH3A | Isoform 1 of Rabphilin-3A | 0.0018 | 4.7 | 2 | 75 |
| IPI00002280 | PCSK1N | ProSAAS | 0.0020 | 4.8 | 3 | 143 |
| IPI00009253 | NAPA | Alpha-soluble NSF attachment protein | 0.0020 | 3.0 | 3 | 269 |
| IPI00291928 | RAB14 | Ras-related protein Rab-14 | 0.0020 | 2.1 | 4 | 189 |
| IPI00657691 | DNM1 | Isoform 5 of Dynamin-1 | 0.0021 | 3.0 | 25 | 1460 |
| IPI00075248 | CALM3 | Calmodulin | 0.0022 | 2.7 | 8 | 597 |
| IPI00007812 | ATP6V1B2 | V-type proton ATPase subunit B, brain isoform | 0.0022 | 2.8 | 13 | 818 |
| IPI00006482 | ATP1A1 | Isoform Long of Na/K-transporting ATPase alpha-1 | 0.0023 | 2.2 | 38 | 2392 |
| IPI00747849 | ATP1B1 | Isoform 1 of Sodium/potassium-transport ATPase β1 | 0.0024 | 4.7 | 8 | 516 |
| IPI00218493 | HPRT1 | Hypoxanthine-guanine phosphoribosyltransferase | 0.0028 | 4.0 | 3 | 187 |
| IPI00019171 | SH3GL2 | Endophilin-A1 | 0.0028 | 4.0 | 3 | 254 |
| IPI00022021 | PHYHIP | Phytanoyl-CoA hydroxylase-interacting protein | 0.0029 | 6.6 | 2 | 49 |
| IPI00302840 | ATP1A3 | Sodium/potassium-transporting ATPase subunit α-3 | 0.0029 | 2.4 | 37 | 2736 |
| IPI00026846 | SLC6A1 | Sodium- and chloride-dependent GABA transporter 1 | 0.0033 | 8.9 | 2 | 153 |
| IPI00549543 | NCDN | Isoform 1 of Neurochondrin | 0.0034 | 4.6 | 4 | 163 |
| IPI00010343 | SLC8A2 | Sodium/calcium exchanger 2 | 0.0034 | 3.9 | 4 | 201 |
| IPI00300020 | SLC1A2 | Isoform 1 of Excitatory amino acid transporter 2 | 0.0035 | 3.5 | 11 | 938 |
| IPI00220175 | MAPT | Isoform Tau-E of Microtubule-associated protein tau | 0.0035 | 3.2 | 10 | 512 |
| IPI00032230 | EPB41L3 | Isoform A of Band 4.1-like protein 3 | 0.0036 | 3.2 | 15 | 696 |
| IPI00219628 | PRKCB | Isoform Beta-II of Protein kinase C beta type | 0.0036 | 3.3 | 3 | 137 |
| IPI00293836 | CADM2 | Isoform 3 of Cell adhesion molecule 2 | 0.0038 | 4.4 | 3 | 145 |
| IPI00220642 | YWHAG | 14-3-3 protein gamma | 0.0040 | 2.3 | 14 | 949 |
| IPI00291005 | MDH1 | Malate dehydrogenase, cytoplasmic | 0.0040 | 2.2 | 11 | 620 |
| IPI00220173 | MAPT | Isoform Tau-B of Microtubule-associated protein tau | 0.0041 | 3.4 | 10 | 537 |
| IPI00034159 | ATP6V0D1 | V-type proton ATPase subunit d 1 | 0.0042 | 3.3 | 8 | 424 |
| IPI00001568 | ATP6V1D | V-type proton ATPase subunit D | 0.0043 | 5.9 | 2 | 50 |
| IPI00017731 | SEPT5 | Septin-5 | 0.0043 | 4.3 | 9 | 534 |
| IPI00743576 | ATP6V0A1 | Isoform 2 V-type proton ATPase 116 kDa subunit a 1 | 0.0047 | 3.5 | 17 | 1090 |
| IPI00177884 | SYNGAP1 | Isoform 1 of Ras GTPase-activating protein SynGAP | 0.0049 | 2.4 | 2 | 50 |
| IPI00384187 | SEPT3 | Isoform 1 of Neuronal-specific septin-3 | 0.0053 | 3.5 | 4 | 225 |
| IPI00023302 | SYN2 | Isoform IIa of Synapsin-2 | 0.0054 | 4.6 | 11 | 587 |
| IPI00252731 | DPP6 | Isoform DPPX-S dipeptidyl aminopeptidase protein 6 | 0.0057 | 3.0 | 4 | 177 |
| IPI00011932 | HSPA12A | Heat shock 70 kDa protein 12A | 0.0058 | 5.2 | 5 | 195 |
| IPI00160552 | TNR | Isoform 1 of Tenascin-R | 0.0059 | 3.0 | 6 | 309 |
| IPI00084828 | STXBP1 | Isoform 1 of Syntaxin-binding protein 1 | 0.0061 | 2.7 | 39 | 2872 |
| IPI00877014 | GDAP1 | Ganglioside-induced differentiation-assoc protein 1 a | 0.0072 | 2.1 | 10 | 557 |
| IPI00644025 | SV2A | Isoform 1 of Synaptic vesicle glycoprotein 2A | 0.0078 | 2.7 | 5 | 166 |
| IPI00218130 | PYGM | Glycogen phosphorylase, muscle form | 0.0089 | 2.6 | 3 | 101 |
| IPI00017597 | MAPRE3 | Isoform 1 Microtubule-assoc protein RP/EB member 3 | 0.0094 | 2.4 | 3 | 132 |
| IPI00017510 | MT-CO2 | Cytochrome c oxidase subunit 2 | 0.0103 | 3.0 | 7 | 382 |
| IPI00023598 | TUBB4 | Tubulin beta-4 chain | 0.0103 | 2.4 | 32 | 2850 |
| IPI00296191 | ATP6V1H | Isoform 1 of V-type proton ATPase subunit H | 0.0107 | 2.8 | 3 | 176 |
| IPI00032402 | ATP8A1 | Isoform Long of phospholipid-transporting ATPase IA | 0.0118 | 4.1 | 2 | 80 |
| IPI00784156 | AP2B1 | Isoform 1 of AP-2 complex subunit beta-1 | 0.0127 | 2.1 | 22 | 1205 |
| IPI00791716 | ATP6V0A1 | 27 kDa protein | 0.0128 | 3.1 | 7 | 453 |
| IPI00552419 | PCCA | similar to Propionyl-CoA carboxylase alpha chain | 0.0133 | 2.7 | 5 | 189 |
| IPI00103530 | ATL1 | Atlastin-1 | 0.0140 | 2.6 | 2 | 118 |
| IPI00005614 | SPTBN1 | Isoform Long of Spectrin beta chain, brain 1 | 0.0154 | 2.3 | 79 | 5317 |
| IPI00641706 | TUBB6 | 46 kDa protein | 0.0157 | 2.3 | 18 | 1353 |
| IPI00027770 | SYP | Synaptophysin | 0.0158 | 3.8 | 2 | 99 |
| IPI00020356 | MAP1A | 331 kDa protein | 0.0163 | 2.2 | 3 | 141 |
| IPI00220993 | CNP | CNPI 2,3-cyclic-nucleotide3 phosphodiesterase | 0.0165 | 2.2 | 36 | 2484 |
| IPI00744194 | - | ATPase, P-type transporter family protein | 0.0166 | 2.2 | 5 | 320 |
| IPI00871535 | SPTAN1 | Isoform 2 of Spectrin alpha chain, brain | 0.0172 | 2.2 | 77 | 5080 |
| IPI00003971 | RTN1 | Isoform RTN1-A of Reticulon-1 | 0.0172 | 3.4 | 5 | 237 |
| IPI00395510 | NCALD | Neurocalcin-delta | 0.0188 | 2.7 | 2 | 84 |
| IPI00219813 | RTN1 | Isoform RTN1-C of Reticulon-1 | 0.0189 | 3.5 | 3 | 183 |
| IPI00470535 | CACNA2D1 | Dihydropyridine receptor alpha 2 subunit | 0.0197 | 3.4 | 2 | 101 |
| IPI00007752 | TUBB2C | Tubulin beta-2C chain | 0.0216 | 2.1 | 30 | 2698 |
| IPI00925250 | ABHD11 | ABHD11 Protein | 0.0217 | 2.6 | 2 | 112 |
| IPI00292496 | - | Tubulin beta-8 chain | 0.0224 | 2.2 | 12 | 730 |
| IPI00019901 | ADD1 | Isoform 1 of Alpha-adducin | 0.0236 | 2.6 | 8 | 382 |
| IPI00942474 | - | 50 kDa protein | 0.0242 | 2.2 | 10 | 706 |
| IPI00007750 | TUBA4A | Tubulin alpha-4A chain | 0.0261 | 2.1 | 29 | 2291 |
| IPI00930688 | TUBA1B | Tubulin alpha-1B chain | 0.0267 | 2.0 | 34 | 2741 |
| IPI00013475 | TUBB2A | Tubulin beta-2A chain | 0.0280 | 2.1 | 31 | 2645 |
| IPI00479997 | STMN1 | Stathmin-1 | 0.0291 | 2.2 | 3 | 85 |
| IPI00218667 | STMN2 | Stathmin-2 | 0.0291 | 2.2 | 3 | 85 |
| IPI00028509 | GNG7 | Guanine nucleotide-binding protein subunit gamma-7 | 0.0296 | 2.1 | 2 | 108 |
| IPI00219685 | YJEFN3 | NADH dehydrogenase(ubiquinone) 1α subcomplex, 13 | 0.0301 | 2.1 | 6 | 291 |
| IPI00790571 | CLTA | Isoform 3 of Clathrin light chain A | 0.0307 | 3.0 | 3 | 101 |
| IPI00011654 | TUBB | Tubulin beta chain | 0.0320 | 2.0 | 29 | 2361 |
| IPI00013683 | TUBB3 | Tubulin beta-3 chain | 0.0352 | 2.1 | 26 | 1967 |
| IPI00470619 | GNG2 | Guanine nucleotide-binding protein subunit gamma-2 | 0.0492 | 2.3 | 2 | 65 |
| **OTHER PROTEINS IDENTIFIED in GBM** | | | | | | |
| IPI00031169 | RAB2A | Ras-related protein Rab-2A | 3.97E-05 | 1.8 | 5 | 319 |
| IPI00016342 | RAB7A | Ras-related protein Rab-7a | 0.0002 | 1.7 | 2 | 97 |
| IPI00102896 | RAB2B | Ras-related protein Rab-2B | 0.0003 | 1.8 | 5 | 225 |
| IPI00414676 | HSP90AB1 | Heat shock protein HSP 90-beta | 0.0008 | 1.9 | 19 | 950 |
| IPI00003269 | ACTBL2 | Beta-actin-like protein 2 | 0.0016 | 2.0 | 9 | 386 |
| IPI00028091 | ACTR3 | Actin-related protein 3 | 0.0020 | 1.9 | 3 | 243 |
| IPI00003021 | ATP1A2 | Sodium/potassium-transporting ATPase subunit α-2 | 0.0023 | 1.9 | 29 | 2017 |
| IPI00021439 | ACTB | Actin, cytoplasmic 1 | 0.0029 | 2.0 | 25 | 1842 |
| IPI00027497 | GPI | Glucose-6-phosphate isomerase | 0.0035 | 1.7 | 14 | 774 |
| IPI00479743 | POTEE | Isoform 1 of POTE ankyrin domain family member E | 0.0036 | 1.7 | 9 | 810 |
| IPI00604590 | NME1 | Nucleoside diphosphate kinase | 0.0040 | 1.7 | 7 | 343 |
| IPI00027175 | SRI | Sorcin | 0.0042 | 1.8 | 2 | 100 |
| IPI00219078 | ATP2A2 | Sarcoplasmic/endoplasmic reticulum Ca2+ ATPase 2 | 0.0043 | 1.7 | 8 | 309 |
| IPI00015148 | RAP1B | Ras-related protein Rap-1b | 0.0047 | 1.7 | 6 | 281 |
| IPI00012048 | NME1 | Isoform 1 of Nucleoside diphosphate kinase A | 0.0051 | 1.6 | 6 | 317 |
| IPI00016513 | RAB10 | Ras-related protein Rab-10 | 0.0059 | 1.7 | 6 | 307 |
| IPI00554521 | FTH1 | Ferritin heavy chain | 0.0078 | 1.9 | 9 | 411 |
| IPI00448725 | RAB4B | HCG1995540, isoform CRA_b | 0.0083 | 2.0 | 2 | 87 |
| IPI00411680 | PCMT1 | Protein-L-isoaspartate O-methyltransferase 1 | 0.0083 | 1.9 | 6 | 280 |
| IPI00021428 | ACTA1 | Actin, alpha skeletal muscle | 0.0084 | 2.0 | 18 | 928 |
| IPI00016891 | RAB6B | Ras-related protein Rab-6B | 0.0107 | 1.9 | 2 | 101 |
| IPI00219446 | PEBP1 | Phosphatidylethanolamine-binding protein 1 | 0.0116 | 1.9 | 10 | 689 |
| IPI00012490 | ATP2B4 | Plasma membrane calcium-transporting ATPase 4 | 0.0120 | 2.0 | 14 | 776 |
| IPI00553177 | SERPINA1 | Isoform 1 of Alpha-1-antitrypsin | 0.0126 | 1.5 | 13 | 617 |
| IPI00328415 | CYB5R3 | highly similar to NADH-cytochrome b5 reductase | 0.0130 | 1.5 | 3 | 153 |
| IPI00021812 | AHNAK | Neuroblast differentiation-associated protein AHNAK | 0.0136 | 1.7 | 5 | 155 |
| IPI00009790 | PFKP | PFKP 6-phosphofructokinase type C | 0.0162 | 1.9 | 5 | 182 |
| IPI00012011 | CFL1 | Cofilin-1 | 0.0170 | 1.5 | 10 | 957 |
| IPI00337415 | GNAI1 | Guanine nucleotide-binding protein G(i), α-1 subunit | 0.0171 | 1.6 | 14 | 827 |
| IPI00003348 | GNB2 | Guanine nucleotide-binding protein β-2 | 0.0175 | 1.8 | 15 | 1015 |
| IPI00179589 | - | 14 kDa protein | 0.0181 | 12.0 | 2 | 128 |
| IPI00295386 | CBR1 | Carbonyl reductase [NADPH] 1 | 0.0186 | 1.5 | 7 | 455 |
| IPI00296190 | C10orf58 | Uncharacterized protein C10orf58 | 0.0187 | 1.9 | 3 | 106 |
| IPI00220416 | UQCRB | Cytochrome b-c1 complex subunit 7 | 0.0207 | 1.9 | 7 | 360 |
| IPI00026237 | MAG | Myelin-associated glycoprotein | 0.0211 | 1.8 | 5 | 208 |
| IPI00290928 | GNA13 | Guanine nucleotide-binding protein subunit alpha-13 | 0.0216 | 1.9 | 2 | 93 |
| IPI00009368 | SFXN1 | Sideroflexin-1 | 0.0219 | 1.8 | 10 | 510 |
| IPI00021263 | YWHAZ | 14-3-3 protein zeta/delta | 0.0223 | 1.8 | 13 | 951 |
| IPI00220828 | TMSB4X | Thymosin beta-4 | 0.0223 | 1.9 | 4 | 260 |
| IPI00005160 | ARPC1B | Actin-related protein 2/3 complex subunit 1B | 0.0225 | 1.9 | 2 | 99 |
| IPI00217048 | SPTBN4 | Isoform 4 of Spectrin beta chain, brain 3 | 0.0227 | 1.8 | 2 | 61 |
| IPI00383449 | RAB15 | Isoform 2 of Ras-related protein Rab-15 | 0.0234 | 1.7 | 2 | 77 |
| IPI00300096 | RAB35 | Ras-related protein Rab-35 | 0.0241 | 1.7 | 3 | 245 |
| IPI00216319 | YWHAH | 14-3-3 protein eta | 0.0263 | 2.0 | 11 | 667 |
| IPI00013847 | UQCRC1 | Cytochrome b-c1 complex subunit 1 | 0.0265 | 1.9 | 26 | 1956 |
| IPI00306301 | PDHA1 | PDHA1 | 0.0273 | 2.0 | 17 | 901 |
| IPI00305166 | SDHA | similar to Succinate dehydrogenase flavoprotein | 0.0277 | 1.9 | 12 | 821 |
| IPI00006721 | OPA1 | Isoform 1 of Dynamin-like 120 kDa protein | 0.0277 | 1.9 | 23 | 1424 |
| IPI00220342 | DDAH1 | Dimethylarginine dimethylaminohydrolase 1 | 0.0289 | 1.9 | 7 | 370 |
| IPI00646909 | TUBA8 | Tubulin alpha-8 chain | 0.0300 | 2.0 | 17 | 1375 |
| IPI00029133 | ATP5F1 | ATP synthase subunit b | 0.0305 | 1.8 | 11 | 760 |
| IPI00291006 | MDH2 | Malate dehydrogenase | 0.0309 | 1.9 | 24 | 1646 |
| IPI00333776 | NRCAM | Isoform 1 of Neuronal cell adhesion molecule | 0.0309 | 1.6 | 3 | 156 |
| IPI00412607 | RPL35 | 60S ribosomal protein L35 | 0.0315 | 1.5 | 2 | 47 |
| IPI00218343 | TUBA1C | Tubulin alpha-1C chain | 0.0317 | 2.0 | 32 | 2457 |
| IPI00784614 | SEPT9 | Septin 9 isoform a | 0.0326 | 1.9 | 10 | 382 |
| IPI00478410 | ATP5C1 | Isoform Liver of ATP synthase subunit gamma | 0.0330 | 2.0 | 16 | 1004 |
| IPI00645078 | UBA1 | Ubiquitin-like modifier-activating enzyme 1 | 0.0330 | 1.6 | 5 | 181 |
| IPI00022793 | HADHB | Trifunctional enzyme subunit beta | 0.0334 | 1.9 | 21 | 1137 |
| IPI00435020 | NCAM1 | Isoform 2 of Neural cell adhesion molecule 1 | 0.0335 | 2.0 | 17 | 1088 |
| IPI00180675 | TUBA1A | Tubulin alpha-1A chain | 0.0341 | 2.0 | 34 | 2743 |
| IPI00930124 | IGHV4-31 | Putative uncharacterized protein DKFZp686C11235 | 0.0349 | 1.9 | 12 | 944 |
| IPI00025366 | CS | Citrate synthase | 0.0350 | 1.8 | 17 | 993 |
| IPI00018352 | UCHL1 | Ubiquitin carboxyl-terminal hydrolase isozyme L1 | 0.0366 | 2.0 | 14 | 882 |
| IPI00006091 | DMD | Isoform 4 of Dystrophin | 0.0367 | 1.5 | 3 | 70 |
| IPI00031370 | TUBB2B | Tubulin beta-2B chain | 0.0378 | 2.0 | 31 | 2649 |
| IPI00305383 | UQCRC2 | Cytochrome b-c1 complex subunit 2 | 0.0379 | 2.0 | 18 | 1480 |
| IPI00022488 | HPX | Hemopexin | 0.0380 | 1.8 | 3 | 98 |
| IPI00455599 | HSP90AB2P | Similar to Heat shock protein HSP 90-beta | 0.0381 | 1.6 | 6 | 219 |
| IPI00168728 | IGHG3 | FLJ00385 protein (Fragment) | 0.0385 | 1.8 | 9 | 449 |
| IPI00014898 | PLEC1 | Isoform 1 of Plectin-1 | 0.0403 | 1.8 | 24 | 1087 |
| IPI00217871 | ALDH4A1 | Delta-1-pyrroline-5-carboxylate dehydrogenase | 0.0406 | 1.9 | 12 | 713 |
| IPI00479722 | PSME1 | Proteasome activator complex subunit 1 | 0.0409 | 1.6 | 2 | 85 |
| IPI00219664 | MOG | Isoform 2 of Myelin-oligodendrocyte glycoprotein | 0.0410 | 1.5 | 7 | 393 |
| IPI00328257 | AP1B1 | Isoform A of AP-1 complex subunit beta-1 | 0.0416 | 1.7 | 8 | 344 |
| IPI00019346 | RAP2A | Ras-related protein Rap-2a | 0.0430 | 1.9 | 2 | 112 |
| IPI00420108 | DLST | Dihydrolipoyllysine-residue succinyltransferase | 0.0431 | 2.0 | 7 | 448 |
| IPI00216318 | YWHAB | Isoform Long of 14-3-3 protein beta/alpha | 0.0437 | 1.7 | 14 | 1145 |
| IPI00220637 | SARS | Seryl-tRNA synthetase, cytoplasmic | 0.0441 | 1.9 | 2 | 129 |
| IPI00012451 | GNB4 | Guanine nucleotide-binding protein subunit beta-4 | 0.0452 | 1.6 | 9 | 317 |
| IPI00413641 | AKR1B1 | Aldose reductase | 0.0462 | 1.4 | 2 | 49 |
| IPI00914938 | AP2A2 | Isoform 1 of AP-2 complex subunit alpha-2 | 0.0465 | 1.7 | 8 | 454 |
| IPI00216308 | VDAC1 | Voltage-dependent anion-selective channel protein 1 | 0.0466 | 1.9 | 21 | 1744 |
| IPI00220301 | PRDX6 | Peroxiredoxin-6 | 0.0467 | 1.7 | 5 | 203 |
| IPI00830132 | IGHG4 | Putative uncharacterized protein IGHG4 (Fragment) | 0.0476 | 1.8 | 7 | 427 |
| IPI00031522 | HADHA | Trifunctional enzyme subunit alpha | 0.0485 | 1.6 | 36 | 2306 |
| IPI00022977 | CKB | Creatine kinase B-type | 0.0488 | 1.6 | 22 | 1634 |
| IPI00642329 | MFN2 | Isoform 1 of Mitofusin-2 | 0.0499 | 1.6 | 2 | 86 |
| IPI00942271 | AP2A1 | adaptor-related protein complex 2, alpha 1 isoform 1 | 0.0501 | 1.7 | 9 | 398 |
| IPI00288947 | GNAQ | Guanine nucleotide-binding protein G(q) subunit α | 0.0510 | 1.5 | 2 | 83 |
| IPI00646779 | TUBB6 | TUBB6 protein | 0.0511 | 1.9 | 17 | 1443 |
| IPI00784044 | MCCC2 | Methylcrotonoyl-CoA carboxylase beta chain | 0.0513 | 1.9 | 6 | 282 |
| IPI00470674 | CYB5R1 | NADH-cytochrome b5 reductase 1 | 0.0521 | 2.0 | 6 | 278 |
| IPI00375339 | ATP1A4 | Isoform 1 of Na/K-transporting ATPase subunit α-4 | 0.0522 | 1.6 | 9 | 730 |
| IPI00930442 | IGHG4 | Putative uncharacterized protein DKFZp686M24218 | 0.0523 | 1.7 | 6 | 252 |
| IPI00010779 | TPM4 | Isoform 1 of Tropomyosin alpha-4 chain | 0.0528 | 1.5 | 6 | 287 |
| IPI00386524 | IGHA1 | highly similar to Protein Tro alpha1 H,myeloma | 0.0536 | 2.9 | 5 | 232 |
| IPI00219661 | PLP1 | Isoform 1 of Myelin proteolipid protein | 0.0537 | 1.0 | 6 | 293 |
| IPI00216592 | HNRNPC | Heterogeneous nuclear ribonucleoproteins C1/C2 | 0.0537 | 3.8 | 2 | 92 |
| IPI00000041 | RHOB | Rho-related GTP-binding protein RhoB | 0.0551 | 2.3 | 4 | 207 |
| IPI00026182 | CAPZA2 | F-actin-capping protein subunit alpha-2 | 0.0557 | 1.6 | 2 | 92 |
| IPI00026185 | CAPZB | Isoform 1 of F-actin-capping protein subunit beta | 0.0558 | 2.2 | 2 | 137 |
| IPI00177728 | CNDP2 | Cytosolic non-specific dipeptidase | 0.0568 | 1.7 | 3 | 123 |
| IPI00419585 | PPIA | Peptidyl-prolyl cis-trans isomerase A | 0.0569 | 1.7 | 12 | 860 |
| IPI00032292 | TIMP1 | Metalloproteinase inhibitor 1 | 0.0576 | 2.9 | 2 | 83 |
| IPI00375577 | TMEM65 | Transmembrane protein 65 | 0.0582 | 2.3 | 2 | 56 |
| IPI00015602 | TOMM70A | Mitochondrial import receptor subunit TOM70 | 0.0587 | 1.7 | 12 | 615 |
| IPI00064966 | ATP6V1G3 | Isoform 4 of V-type proton ATPase subunit G 3 | 0.0607 | 1.0 | 2 | 23 |
| IPI00217519 | RALA | Ras-related protein Ral-A | 0.0615 | 1.6 | 4 | 331 |
| IPI00022418 | FN1 | Isoform 1 of Fibronectin | 0.0638 | 2.2 | 4 | 153 |
| IPI00021854 | APOA2 | Apolipoprotein A-II | 0.0641 | 2.0 | 5 | 240 |
| IPI00414384 | HSDL2 | Hydroxysteroid dehydrogenase-like protein 2 | 0.0641 | 2.0 | 7 | 423 |
| IPI00232891 | FNDC1 | 179 kDa protein | 0.0643 | 1.8 | 2 | 51 |
| IPI00013508 | ACTN1 | Alpha-actinin-1 | 0.0644 | 2.9 | 21 | 1145 |
| IPI00554681 | NDUFA5 | NADH dehydrogenase [ubiquinone] 1 α- subunit 5 | 0.0658 | 1.8 | 6 | 522 |
| IPI00414123 | CRMP1 | Dihydropyrimidinase-related protein 1 | 0.0660 | 1.7 | 8 | 389 |
| IPI00218319 | TPM3 | Isoform 2 of Tropomyosin alpha-3 chain | 0.0663 | 1.4 | 8 | 402 |
| IPI00011416 | ECH1 | Delta(3,5)-Delta(2,4)-dienoyl-CoA isomerase | 0.0665 | 1.9 | 7 | 359 |
| IPI00060715 | KCTD12 | BTB/POZ domain-containing protein KCTD12 | 0.0666 | 5.2 | 2 | 58 |
| IPI00220578 | GNAI3 | Guanine nucleotide-binding protein G(k) subunit α | 0.0668 | 1.4 | 8 | 504 |
| IPI00797709 | CORO1C | 18 kDa protein | 0.0670 | 2.1 | 2 | 118 |
| IPI00449049 | PARP1 | Poly [ADP-ribose] polymerase 1 | 0.0675 | 9.1 | 5 | 186 |
| IPI00007084 | SLC25A13 | Calcium-binding carrier protein Aralar2 | 0.0675 | 1.8 | 8 | 381 |
| IPI00645805 | IVD | Isovaleryl-CoA dehydrogenase | 0.0675 | 2.5 | 5 | 189 |
| IPI00024742 | UQCRQ | Cytochrome b-c1 complex subunit 8 | 0.0683 | 1.8 | 3 | 164 |
| IPI00386755 | ERO1L | ERO1-like protein alpha | 0.0686 | 3.6 | 2 | 109 |
| IPI00022891 | SLC25A4 | ADP/ATP translocase 1 | 0.0688 | 1.9 | 25 | 1668 |
| IPI00179415 | PPP3CA | Ser/thr-protein phosphatase 2B catalytic α-isoform | 0.0696 | 1.7 | 7 | 245 |
| IPI00255052 | NDUFB9 | NADH dehydrogenase [ubiquinone] 1 beta subunit 9 | 0.0698 | 2.0 | 7 | 275 |
| IPI00215948 | CTNNA1 | Isoform 1 of Catenin alpha-1 | 0.0703 | 1.7 | 2 | 57 |
| IPI00008214 | NDUFV3 | NADH dehydrogenase [ubiquinone] flavoprotein 3 | 0.0721 | 3.3 | 3 | 136 |
| IPI00216478 | MBP | Isoform 6 of Myelin basic protein | 0.0727 | 1.2 | 12 | 733 |
| IPI00399007 | IGHG2 | Putative uncharacterized protein DKFZp686I04196 | 0.0730 | 1.6 | 9 | 431 |
| IPI00021907 | MBP | Isoform 1 of Myelin basic protein | 0.0734 | 1.2 | 12 | 684 |
| IPI00444375 | RHCE | Rh blood group, CcEe antigens | 0.0734 | 10.3 | 2 | 65 |
| IPI00031622 | CHCHD6 | Coiled-helix-coiled-helix domain-containing protein 6 | 0.0736 | 1.7 | 4 | 240 |
| IPI00011604 | GCSH | Glycine cleavage system H protein | 0.0742 | 1.8 | 2 | 176 |
| IPI00021842 | APOE | Apolipoprotein E | 0.0753 | 1.9 | 2 | 78 |
| IPI00029744 | SSBP1 | Single-stranded DNA-binding protein | 0.0755 | 1.4 | 8 | 587 |
| IPI00179109 | SIRT2 | Isoform 1 of NAD-dependent deacetylase sirtuin-2 | 0.0760 | 1.4 | 3 | 74 |
| IPI00216139 | SEPT6 | Isoform I of Septin-6 | 0.0766 | 1.7 | 5 | 206 |
| IPI00156689 | VAT1 | Synaptic vesicle membrane protein VAT-1 homolog | 0.0768 | 1.9 | 3 | 67 |
| IPI00296022 | UQCRH | Cytochrome b-c1 complex subunit 6 | 0.0773 | 1.8 | 3 | 264 |
| IPI00219568 | PGK2 | Phosphoglycerate kinase 2 | 0.0783 | 1.5 | 5 | 242 |
| IPI00453476 | - | 29 kDa protein | 0.0787 | 1.5 | 8 | 465 |
| IPI00217143 | SDHA | 57 kDa protein | 0.0788 | 1.8 | 12 | 748 |
| IPI00008994 | NDRG2 | Isoform 1 of Protein NDRG2 | 0.0795 | 1.7 | 4 | 256 |
| IPI00298423 | PDHX | Pyruvate dehydrogenase protein X component, | 0.0801 | 1.8 | 5 | 240 |
| IPI00294911 | SDHB | Succinate dehydrogenase [ubiquinone] iron-sulfur | 0.0808 | 1.9 | 5 | 213 |
| IPI00295992 | ATAD3A | ATPase family AAA domain-containing protein 3A | 0.0816 | 1.5 | 8 | 370 |
| IPI00022429 | ORM1 | ORM1 Alpha-1-acid glycoprotein 1 | 0.0823 | 1.8 | 4 | 204 |
| IPI00095891 | GNAS | Guanine nucleotide-binding protein G(s) subunit α | 0.0829 | 1.4 | 4 | 225 |
| IPI00305486 | AMPH | Isoform 1 of Amphiphysin | 0.0840 | 1.8 | 5 | 236 |
| IPI00216704 | SPTB | Isoform 2 of Spectrin beta chain, erythrocyte | 0.0845 | 1.6 | 44 | 2136. |
| IPI00005159 | ACTR2 | Actin-related protein 2 | 0.0860 | 1.6 | 2 | 102 |
| IPI00382470 | HSP90AA1 | Heat shock 90kDa protein 1, alpha isoform 1 | 0.0863 | 1.4 | 23 | 1142 |
| IPI00220300 | ATP5J2 | Isoform 1 of ATP synthase subunit f | 0.0872 | 1.9 | 4 | 193 |
| IPI00019580 | PLG | Plasminogen | 0.0885 | 5.0 | 11 | 555 |
| IPI00555902 | OCIAD2 | Isoform 1 of OCIA domain-containing protein 2 | 0.0888 | 13.7 | 4 | 153 |
| IPI00021304 | KRT2 | Keratin, type II cytoskeletal 2 epidermal | 0.0929 | 2.2 | 8 | 483 |
| IPI00027448 | ATP5L | ATP synthase subunit g | 0.0931 | 1.7 | 6 | 414 |
| IPI00789008 | FLOT2 | Flotillin-2 | 0.0932 | 1.5 | 3 | 77 |
| IPI00005089 | TMOD2 | Tropomodulin-2 | 0.0937 | 2.8 | 3 | 181 |
| IPI00479217 | HNRNPU | Heterogeneous nuclear ribonucleoprotein U | 0.0973 | 3.8 | 4 | 157 |
| IPI00296360 | DCLK2 | Isoform 3 of Serine/threonine-protein kinase DCLK2 | 0.0981 | 2.2 | 2 | 154 |
| IPI00554811 | ARPC4 | Actin-related protein 2/3 complex subunit 4 | 0.0990 | 1.6 | 3 | 118 |
| IPI00479186 | PKM2 | Isoform M2 of Pyruvate kinase isozymes M1/M2 | 0.1005 | 1.4 | 32 | 2374 |
| IPI00887739 | LOC1001335 | LOC100133511 hypothetical protein, partial | 0.1021 | 2.0 | 5 | 236 |
| IPI00783987 | C3 | Complement C3 (Fragment) | 0.1021 | 2.0 | 5 | 236 |
| IPI00029264 | CYC1 | Cytochrome c1, heme protein | 0.1032 | 1.7 | 8 | 470 |
| IPI00019884 | ACTN2 | Alpha-actinin-2 | 0.1045 | 2.4 | 7 | 311 |
| IPI00022395 | C9 | Complement component C9 | 0.1049 | 5.6 | 3 | 144 |
| IPI00221234 | ALDH7A1 | Aldehyde dehydrogenase 7 family, member A1 | 0.1058 | 1.6 | 12 | 673 |
| IPI00298547 | PARK7 | Protein DJ-1 | 0.1058 | 1.6 | 5 | 456 |
| IPI00218487 | GJA1 | Gap junction alpha-1 protein | 0.1084 | 1.4 | 3 | 197 |
| IPI00169383 | PGK1 | Phosphoglycerate kinase 1 | 0.1087 | 1.4 | 15 | 1101 |
| IPI00001734 | C8orf62 | Phosphoserine aminotransferase | 0.1093 | 4.0 | 4 | 127 |
| IPI00008528 | MT-ATP6 | ATP synthase protein 8 | 0.1096 | 1.4 | 2 | 44 |
| IPI00465256 | AK3 | GTP:AMP phosphotransferase | 0.1116 | 1.7 | 2 | 62 |
| IPI00007047 | S100A8 | Protein S100-A8 | 0.1117 | 7.2 | 2 | 69 |
| IPI00013895 | S100A11 | Protein S100-A11 | 0.1118 | 1.6 | 3 | 190 |
| IPI00294398 | HADH | Hydroxyacyl-coenzyme A dehydrogenase | 0.1130 | 1.6 | 12 | 666 |
| IPI00793874 | SFXN3 | Sideroflexin 3 | 0.1132 | 1.5 | 10 | 527 |
| IPI00217561 | ITGB1 | Isoform Beta-1C of Integrin beta-1 | 0.1132 | 5.3 | 2 | 84 |
| IPI00291467 | SLC25A6 | ADP/ATP translocase 3 | 0.1147 | 1.7 | 24 | 1601 |
| IPI00218733 | SOD1 | Superoxide dismutase [Cu-Zn] | 0.1147 | 1.6 | 6 | 532 |
| IPI00021369 | CRYAB | Alpha-crystallin B chain | 0.1179 | 3.1 | 13 | 844 |
| IPI00219018 | GAPDH | Glyceraldehyde-3-phosphate dehydrogenase | 0.1186 | 1.4 | 26 | 2327 |
| IPI00927156 | ARPC2 | 19 kDa protein | 0.1195 | 1.4 | 2 | 54 |
| IPI00010154 | GDI1 | Rab GDP dissociation inhibitor alpha | 0.1211 | 1.4 | 20 | 1122 |
| IPI00239077 | HINT1 | Histidine triad nucleotide-binding protein 1 | 0.1226 | 1.6 | 2 | 73 |
| IPI00303568 | PTGES2 | Prostaglandin E synthase 2 | 0.1226 | 1.3 | 4 | 129 |
| IPI00026219 | CPSF1 | Cleavage & polyadenylation specificity factor subunit1 | 0.1245 | 2.1 | 2 | 50 |
| IPI00019345 | RAP1A | Ras-related protein Rap-1A | 0.1252 | 1.5 | 6 | 305 |
| IPI00021766 | RTN4 | Isoform 1 of Reticulon-4 | 0.1253 | 1.4 | 8 | 384 |
| IPI00289862 | SCRN1 | Secernin-1 | 0.1263 | 1.3 | 4 | 173 |
| IPI00017454 | TUBA4B | Putative tubulin-like protein alpha-4B | 0.1266 | 2.0 | 2 | 118 |
| IPI00015964 | GAP43 | Neuromodulin | 0.1267 | 2.0 | 16 | 1209 |
| IPI00217683 | AKAP12 | A kinase (PRKA) anchor protein 12 isoform 2 | 0.1283 | 1.9 | 2 | 65 |
| IPI00293971 | ATP1B2 | Sodium/potassium-transporting ATPase subunit β-2 | 0.1286 | 1.6 | 5 | 304 |
| IPI00942979 | TKT | Transketolase | 0.1308 | 1.9 | 11 | 601 |
| IPI00011229 | CTSD | Cathepsin D | 0.1317 | 1.6 | 9 | 518 |
| IPI00031461 | GDI2 | highly similar to Rab GDP dissociation inhibitor beta | 0.1339 | 1.3 | 12 | 771 |
| IPI00418262 | ALDOC | Fructose-bisphosphate aldolase | 0.1362 | 1.5 | 20 | 1161 |
| IPI00220059 | NDUFB4 | NADH dehydrogenase [ubiquinone] 1 beta subunit 4 | 0.1377 | 1.9 | 2 | 77 |
| IPI00029111 | DPYSL3 | Collapsin response mediator protein 4 long variant | 0.1390 | 1.5 | 12 | 586 |
| IPI00290770 | CCT3 | Chaperonin containing TCP1, subunit 3 isoform b | 0.1436 | 1.5 | 2 | 127 |
| IPI00060031 | ARL8A | ADP-ribosylation factor-like protein 8A | 0.1447 | 1.2 | 2 | 77 |
| IPI00024915 | PRDX5 | Isoform Mitochondrial of Peroxiredoxin-5 | 0.1452 | 1.4 | 9 | 642 |
| IPI00922081 | - | Fructose-bisphosphate aldolase | 0.1494 | 1.5 | 19 | 1134 |
| IPI00171445 | ATAD1 | ATPase family AAA domain-containing protein 1 | 0.1512 | 1.7 | 2 | 190 |
| IPI00018871 | ARL8B | similar to ADP-ribosylation factor-like protein 8B | 0.1525 | 1.2 | 3 | 107 |
| IPI00005158 | LONP1 | Lon protease homolog, mitochondrial | 0.1531 | 1.4 | 11 | 596 |
| IPI00028031 | ACADVL | highly similar to long-chain acyl-CoAdehydrogenase | 0.1535 | 1.4 | 13 | 721 |
| IPI00218848 | ATP5I | ATP synthase, H+ transporting, F0 complex, subunit E | 0.1558 | 1.7 | 2 | 126 |
| IPI00607708 | LDHA | Isoform 2 of L-lactate dehydrogenase A chain | 0.1573 | 1.3 | 16 | 789 |
| IPI00886911 | LOC646048 | Similar to actin alpha 1 skeletal muscle protein | 0.1574 | 4.6 | 2 | 134 |
| IPI00444262 | NCL | highly similar to Nucleolin | 0.1584 | 4.1 | 3 | 140 |
| IPI00027350 | PRDX2 | Peroxiredoxin-2 | 0.1585 | 1.3 | 7 | 381 |
| IPI00098902 | OGDH | 2-oxoglutarate dehydrogenase E1 component | 0.1586 | 1.7 | 29 | 1406 |
| IPI00337541 | NNT | NAD(P) transhydrogenase, mitochondrial | 0.1623 | 1.5 | 23 | 1104 |
| IPI00021405 | LMNA | Isoform A of Lamin-A/C | 0.1648 | 4.9 | 13 | 700 |
| IPI00550731 | - | Putative uncharacterized protein | 0.1652 | 1.4 | 5 | 442 |
| IPI00019359 | KRT9 | Keratin, type I cytoskeletal 9 | 0.16583 | 1.6 | 7 | 289 |
| IPI00290078 | KRT4 | Keratin 4 | 0.1663 | 1.5 | 3 | 219 |
| IPI00186966 | BIN1 | Isoform IIA Myc box-dependent-interacting protein 1 | 0.1668 | 1.5 | 4 | 160 |
| IPI00003420 | MAPRE2 | Isoform 1 Microtubule-associated protein RP/EB 2 | 0.1678 | 1.7 | 2 | 121 |
| IPI00007188 | SLC25A5 | ADP/ATP translocase 2 | 0.1712 | 1.6 | 24 | 1676 |
| IPI00027769 | ELANE | Neutrophil elastase | 0.1715 | 1.5 | 4 | 176 |
| IPI00215914 | ARF1 | ADP-ribosylation factor 1 | 0.1731 | 1.5 | 4 | 212 |
| IPI00792375 | ALDOC | Putative uncharacterized protein ALDOC | 0.1732 | 1.4 | 21 | 1198 |
| IPI00026314 | GSN | Isoform 1 of Gelsolin | 0.1745 | 1.2 | 19 | 1092 |
| IPI00219585 | PFKM | Isoform 2 of 6-phosphofructokinase, muscle type | 0.1776 | 1.7 | 4 | 226 |
| IPI00163187 | FSCN1 | Fascin | 0.1811 | 1.4 | 9 | 543 |
| IPI00465248 | ENO1 | Isoform alpha-enolase of Alpha-enolase | 0.1848 | 1.3 | 20 | 1593 |
| IPI00012645 | SPTBN2 | Isoform 1 of Spectrin beta chain, brain 2 | 0.1900 | 1.4 | 7 | 325 |
| IPI00220644 | PKM2 | Isoform M1 of Pyruvate kinase isozymes M1/M2 | 0.1906 | 1.3 | 32 | 2323 |
| IPI00419266 | NDUFA6 | NADH dehydrogenase (ubiquinone) 1α subcomplex 6 | 0.1975 | 1.5 | 3 | 208 |
| IPI00219249 | CNTNAP1 | Contactin-associated protein 1 | 0.1996 | 1.6 | 3 | 148 |
| IPI00000792 | CRYZ | Quinone oxidoreductase | 0.1996 | 1.7 | 5 | 257 |
| IPI00465315 | CYCS | Cytochrome c | 0.2016 | 1.6 | 9 | 612 |
| IPI00016801 | GLUD1 | Glutamate dehydrogenase 1, mitochondrial | 0.2058 | 1.5 | 33 | 2118 |
| IPI00376379 | KRT77 | Keratin 77 | 0.2070 | 2.2 | 2 | 102 |
| IPI00334190 | STOML2 | Stomatin-like protein 2 | 0.2091 | 1.7 | 4 | 280 |
| IPI00295542 | NUCB1 | Nucleobindin-1 | 0.2120 | 1.4 | 2 | 65 |
| IPI00332106 | PBXIP1 | Pre-B-cell leukemia TF-interacting protein 1 | 0.2154 | 2. 8 | 2 | 106 |
| IPI00019888 | ALDH5A1 | Succinate-semialdehyde dehydrogenase, mitochondrial | 0.2196 | 1.4 | 18 | 844 |
| IPI00014850 | PEA15 | Astrocytic phosphoprotein PEA-15 | 0.2201 | 1.3 | 2 | 75 |
| IPI00910754 | LDHA | L-lactate dehydrogenase A isoform 2 | 0.2201 | 1.2 | 12 | 584 |
| IPI00027107 | TUFM | Translation elongation factor, mitochondrial precursor | 0.2202 | 1.3 | 18 | 1178 |
| IPI00005537 | MRPL12 | 39S ribosomal protein L12, mitochondrial | 0.2226 | 1.4 | 3 | 176 |
| IPI00026053 | CLDN11 | Claudin-11 | 0.2245 | 3.1 | 2 | 89 |
| IPI00295240 | NFS1 | Isoform Mitochondrial of Cysteine desulfurase | 0.2297 | 2.1 | 2 | 115 |
| IPI00001541 | TIMM9 | Mitochondrial inner membrane translocase Tim9 | 0.2350 | 1.5 | 3 | 216 |
| IPI00157414 | ENPP6 | Ectonucleotide pyrophosphatase/phosphodiesterase 6 | 0.2353 | 1.5 | 2 | 118 |
| IPI00024266 | MGST3 | Microsomal glutathione S-transferase 3 | 0.2360 | 1.7 | 4 | 262 |
| IPI00020075 | ABHD10 | Abhydrolase domain-containing protein 10 | 0.2360 | 2.0 | 4 | 168 |
| IPI00456429 | UBA52 | Ubiquitin and ribosomal protein L40 precursor | 0.2362 | 1.4 | 6 | 315 |
| IPI00179330 | UBB | Ubiquitin and ribosomal protein S27a precursor | 0.2362 | 1.4 | 6 | 315 |
| IPI00005161 | ARPC2 | Actin-related protein 2/3 complex subunit 2 | 0.2390 | 1.3 | 3 | 74.73 |
| IPI00000816 | YWHAE | 14-3-3 protein epsilon | 0.2405 | 1.3 | 17 | 1109 |
| IPI00386258 | MTCH1 | Isoform 1 of Mitochondrial carrier homolog 1 | 0.2417 | 1.5 | 4 | 167 |
| IPI00015973 | EPB41L2 | Band 4.1-like protein 2 | 0.2437 | 2.9 | 4 | 100 |
| IPI00217966 | LDHA | L-lactate dehydrogenase | 0.2459 | 1.3 | 14 | 715 |
| IPI00442073 | CSRP1 | Cysteine and glycine-rich protein 1 | 0.2469 | 1.5 | 5 | 403 |
| IPI00940553 | HSPA1L | 25 kDa protein | 0.2523 | 1.1 | 4 | 246 |
| IPI00643152 | HSPA1L | highly similar to Heat shock 70 kDa -1L | 0.2523 | 1.1 | 4 | 246 |
| IPI00007959 | PDPN | Isoform 3 of Podoplanin | 0.2557 | 49.9 | 2 | 228 |
| IPI00024993 | ECHS1 | Enoyl-CoA hydratase, mitochondrial | 0.2654 | 1.4 | 9 | 702 |
| IPI00008530 | RPLP0 | 60S acidic ribosomal protein P0 | 0.2664 | 1.3 | 2 | 69 |
| IPI00289501 | VGF | Neurosecretory protein VGF | 0.2667 | 2.3 | 3 | 82 |
| IPI00008998 | PTPLAD1 | Protein tyrosine phosphatase-like protein PTPLAD1 | 0.2689 | 1.3 | 3 | 95 |
| IPI00017292 | CTNNB1 | Isoform 1 of Catenin beta-1 | 0.2715 | 1.2 | 2 | 128 |
| IPI00216298 | TXN | Thioredoxin | 0.2717 | 1.3 | 2 | 107 |
| IPI00220834 | XRCC5 | ATP-dependent DNA helicase 2 subunit 2 | 0.2720 | 2.6 | 6 | 382 |
| IPI00019329 | DYNLL1 | Dynein light chain 1, cytoplasmic | 0.2726 | 1.3 | 2 | 73 |
| IPI00042580 | APOO | Isoform 1 of Apolipoprotein O | 0.2734 | 1.5 | 3 | 135 |
| IPI00219217 | LDHB | L-lactate dehydrogenase B chain | 0.2787 | 1.2 | 13 | 834 |
| IPI00008964 | RAB1B | Ras-related protein Rab-1B | 0.2791 | 1.2 | 5 | 183 |
| IPI00000190 | CD81 | CD81 antigen | 0.2799 | 1.3 | 2 | 211 |
| IPI00465439 | ALDOA | Fructose-bisphosphate aldolase A | 0.2819 | 1.4 | 24 | 1648. |
| IPI00872762 | SUCLG1 | Succinyl-CoA ligase [GDP-forming] subunit alpha | 0.2825 | 1.4 | 3 | 218 |
| IPI00926319 | DLD | highly similar to Dihydrolipoyl dehydrogenase | 0.2842 | 1.4 | 13 | 805 |
| IPI00009771 | LMNB2 | Lamin-B2 | 0.2849 | 2.8 | 2 | 56 |
| IPI00003921 | EPB41 | Isoform 1 of Protein 4.1 | 0.2856 | 1.2 | 3 | 113 |
| IPI00385449 | PRKCA | Protein kinase C alpha type | 0.2861 | 1.5 | 2 | 139 |
| IPI00908791 | LDHA | L-lactate dehydrogenase | 0.2872 | 1.2 | 14 | 562 |
| IPI00465028 | RCTPI1 | Triosephosphate isomerase 1 isoform 2 | 0.2887 | 1.2 | 13 | 930 |
| IPI00020567 | ARHGAP1 | Rho GTPase-activating protein 1 | 0.2889 | 1.6 | 2 | 72 |
| IPI00909143 | DLD | Dihydrolipoyl dehydrogenase | 0.2911 | 1.4 | 13 | 788 |
| IPI00019463 | EIF2AK2 | Interferon-induced, dble-strand RNA-activated kinase | 0.2960 | 1.4 | 2 | 85 |
| IPI00002459 | ANXA6 | Annexin VI isoform 2 | 0.3003 | 1.3 | 22 | 1385 |
| IPI00015911 | DLD | Dihydrolipoyl dehydrogenase, mitochondrial | 0.3005 | 1.4 | 13 | 843 |
| IPI00022334 | OAT | Ornithine aminotransferase, mitochondrial | 0.3016 | 1.5 | 3 | 192 |
| IPI00015423 | MAP1LC3A | Microtubule-assoc proteins 1A/1B light chain 3A | 0.3029 | 1.6 | 2 | 96 |
| IPI00013860 | HIBADH | 3-hydroxyisobutyrate dehydrogenase, mitochondrial | 0.3033 | 1.8 | 4 | 261 |
| IPI00643041 | RAN | GTP-binding nuclear protein Ran | 0.3179 | 1.5 | 2 | 103 |
| IPI00644712 | XRCC6 | ATP-dependent DNA helicase 2 subunit 1 | 0.3203 | 2.6 | 6 | 282 |
| IPI00016457 | CRAT | Isoform 1 of Carnitine O-acetyltransferase | 0.3264 | 1.3 | 2 | 79 |
| IPI00374970 | SEPT10 | Isoform 1 of Septin-10 | 0.3339 | 1.2 | 2 | 71 |
| IPI00554737 | PPP2R1A | Ser/thr-protein phosphatase 2A 65 kDa regulatory Aα | 0.3339 | 1.2 | 6 | 271 |
| IPI00010810 | ETFA | Electron transfer flavoprotein subunit alpha | 0.3397 | 1.3 | 7 | 363 |
| IPI00003865 | HSPA8 | Isoform 1 of Heat shock cognate 71 kDa protein | 0.3413 | 1.2 | 25 | 1570 |
| IPI00024919 | PRDX3 | Thioredoxin-dependent peroxide reductase | 0.3432 | 1.3 | 9 | 552 |
| IPI00016077 | GBAS | Protein NipSnap homolog 2 | 0.3435 | 1.3 | 10 | 490 |
| IPI00843878 | SRGAP3 | KIAA1156 protein | 0.3438 | 1.9 | 2 | 37 |
| IPI00018146 | YWHAQ | 14-3-3 protein theta | 0.3440 | 1.3 | 12 | 684 |
| IPI00304596 | NONO | Non-POU domain-containing octamer-binding protein | 0.3473 | 2.4 | 4 | 189 |
| IPI00027434 | RHOC | Rho-related GTP-binding protein RhoC | 0.3494 | 1.1 | 5 | 275 |
| IPI00304925 | HSPA1B | Heat shock 70 kDa protein 1 | 0.3509 | 1.1 | 8 | 395 |
| IPI00007426 | ARL6IP5 | PRA1 family protein 3 | 0.3550 | 1.2 | 2 | 85 |
| IPI00029468 | ACTR1A | Alpha-centractin | 0.3569 | 1.2 | 2 | 95 |
| IPI00019376 | SEPT11 | cDNA FLJ53374, highly similar to Septin-11 | 0.3575 | 1.4 | 12 | 664 |
| IPI00003815 | ARHGDIA | Rho GDP-dissociation inhibitor 1 | 0.3623 | 1.2 | 4 | 366 |
| IPI00016179 | S100A13 | Protein S100-A13 | 0.3654 | 1.2 | 2 | 91 |
| IPI00027626 | CCT6A | T-complex protein 1 subunit zeta | 0.3661 | 1.2 | 4 | 171 |
| IPI00011107 | IDH2 | Isocitrate dehydrogenase [NADP], mitochondrial | 0.3671 | 1.3 | 26 | 1621 |
| IPI00219673 | GSTK1 | Glutathione S-transferase kappa 1 | 0.3672 | 1.3 | 5 | 291 |
| IPI00027493 | LOC442497 | SLC3A2 4F2 cell-surface antigen heavy chain | 0.3754 | 1.2 | 9 | 502 |
| IPI00056478 | IGSF8 | Isoform 1 of Immunoglobulin superfamily member 8 | 0.3774 | 1.6 | 3 | 162 |
| IPI00329600 | SCCPDH | Probable saccharopine dehydrogenase | 0.3800 | 1.2 | 7 | 374 |
| IPI00411522 | C14orf159 | Isoform 4 of UPF0317 protein C14orf159 | 0.3801 | 1.0 | 8 | 390 |
| IPI00030275 | TRAP1 | Heat shock protein 75 kDa, mitochondrial | 0.3836 | 1.4 | 8 | 310 |
| IPI00013968 | COX7C | Cytochrome c oxidase subunit 7C, mitochondrial | 0.3858 | 2.4 | 2 | 54 |
| IPI00000690 | AIFM1 | Isoform 1 of Apoptosis-inducing factor 1 | 0.3869 | 1.3 | 6 | 264 |
| IPI00019912 | HSD17B4 | Peroxisomal multifunctional enzyme type 2 | 0.3900 | 1.1 | 7 | 404 |
| IPI00010271 | RAC1 | Isoform A Ras-related C3 botulinum toxin substrate 1 | 0.3948 | 1.2 | 3 | 158 |
| IPI00011200 | PHGDH | D-3-phosphoglycerate dehydrogenase | 0.3965 | 1.2 | 5 | 266 |
| IPI00167215 | HEPACAM | Isoform 1 of Hepatocyte cell adhesion molecule | 0.3966 | 1.1 | 3 | 163 |
| IPI00465170 | NT5DC3 | Isoform 1, 5'-nucleotidase domain-containing protein 3 | 0.4011 | 1.5 | 2 | 69 |
| IPI00220362 | HSPE1 | 10 kDa heat shock protein, mitochondrial | 0.4059 | 1.2 | 11 | 486 |
| IPI00296337 | PRKDC | Isoform 1 DNA-dependent protein kinase catalytic | 0.4189 | 1.4 | 5 | 207 |
| IPI00000335 | HINT2 | Histidine triad nucleotide-binding protein 2 | 0.4202 | 1.2 | 2 | 148 |
| IPI00456623 | BCAN | Isoform 1 of Brevican core protein | 0.4222 | 1.1 | 6 | 308 |
| IPI00020495 | MRPS36 | 28S ribosomal protein S36, mitochondrial | 0.4252 | 1.3 | 3 | 86 |
| IPI00016949 | SLC4A4 | Isoform 4 Electrogenic Na+bicarbonate cotransporter 1 | 0.4418 | 1.3 | 3 | 210 |
| IPI00012503 | PSAP | Isoform Sap-mu-0 of Proactivator polypeptide | 0.4479 | 1.4 | 10 | 611 |
| IPI00004902 | ETFB | Isoform 1 of Electron transfer flavoprotein subunit beta | 0.4495 | 1.3 | 9 | 359 |
| IPI00028946 | RTN3 | Isoform 3 of Reticulon-3 | 0.4510 | 1.1 | 2 | 177 |
| IPI00296053 | FH | Isoform Mitochondrial of Fumarate hydratase | 0.4552 | 1.2 | 13 | 822 |
| IPI00010720 | CCT5 | T-complex protein 1 subunit epsilon | 0.4564 | 1.2 | 2 | 131 |
| IPI00010130 | GLUL | Glutamine synthetase | 0.4591 | 1.1 | 8 | 447 |
| IPI00783271 | LRPPRC | Leucine-rich PPR motif-containing protein | 0.4600 | 1.2 | 14 | 818 |
| IPI00010133 | CORO1A | Coronin-1A | 0.4638 | 1.3 | 6 | 264 |
| IPI00473014 | DSTN | Destrin | 0.4679 | 1.4 | 3 | 220 |
| IPI00152426 | SFXN5 | Sideroflexin-5 | 0.4685 | 2.3 | 9 | 377 |
| IPI00784154 | HSPD1 | 60 kDa heat shock protein, mitochondrial | 0.4698 | 1.1 | 47 | 3306 |
| IPI00743859 | CTNNA2 | cDNA FLJ59799, highly similar to Alpha-2 catenin | 0.4713 | 1.4 | 4 | 156 |
| IPI00016339 | RAB5C | Ras-related protein Rab-5C | 0.4794 | 1.1 | 4 | 129 |
| IPI00412785 | BRP44L | Brain protein 44-like protein | 0.4831 | 1.2 | 2 | 64 |
| IPI00019903 | TACO1 | Coiled-coil domain-containing protein 44 | 0.5020 | 1.4 | 2 | 62 |
| IPI00554469 | IMMT | Isoform 2 of Mitochondrial inner membrane protein | 0.5031 | 1.3 | 22 | 1419 |
| IPI00024670 | REEP5 | Receptor expression-enhancing protein 5 | 0.5165 | 1.2 | 2 | 115 |
| IPI00215997 | CD9 | CD9 antigen | 0.5182 | 1.1 | 3 | 179 |
| IPI00328443 | C14orf159 | Isoform 3 of UPF0317 protein C14orf159 | 0.5231 | 1.1 | 8 | 392 |
| IPI00063903 | USMG5 | Up-regulated during skeletal muscle growth protein 5 | 0.5232 | 1.2 | 2 | 121 |
| IPI00219034 | NDUFA8 | NADH dehydrogenase [ubiquinone] 1 alpha subunit 8 | 0.5251 | 1.6 | 2 | 79 |
| IPI00006663 | ALDH2 | Aldehyde dehydrogenase, mitochondrial | 0.5305 | 1.1 | 26 | 1394 |
| IPI00014230 | C1QBP | Component 1 Q subcomponent-binding protein | 0.5385 | 1.1 | 7 | 543 |
| IPI00029739 | CFH | Isoform 1 of Complement factor H | 0.5408 | 1.1 | 2 | 79 |
| IPI00176824 | - | 10 kDa protein | 0.5526 | 1.3 | 2 | 190 |
| IPI00301994 | FAHD2B | Fumarylacetoacetate hydrolase domain-contain prot 2B | 0.5726 | 1.2 | 2 | 115 |
| IPI00019353 | AGK | Isoform 1 of Acylglycerol kinase, mitochondrial | 0.5765 | 1.0 | 3 | 121 |
| IPI00246975 | GSTM3 | Glutathione S-transferase Mu 3 | 0.5819 | 1.4 | 6 | 383 |
| IPI00299399 | S100B | Protein S100-B | 0.5838 | 1.4 | 4 | 295 |
| IPI00022799 | AQP4 | Isoform 2 of Aquaporin-4 | 0.5842 | 1.4 | 7 | 257 |
| IPI00027776 | FECH | Ferrochelatase, mitochondrial | 0.5875 | 1.1 | 2 | 65 |
| IPI00001091 | AFG3L2 | AFG3-like protein 2 | 0.5880 | 1.2 | 5 | 201 |
| IPI00869219 | NEFM | Neurofilament, medium polypeptide 150kDa isoform 1 | 0.5910 | 1.2 | 30 | 1822 |
| IPI00015473 | SLC1A3 | Excitatory amino acid transporter 1 | 0.5996 | 1.0 | 5 | 523 |
| IPI00030363 | ACAT1 | Acetyl-CoA acetyltransferase, mitochondrial | 0.6130 | 1.1 | 15 | 768 |
| IPI00011276 | BCKDHB | 2-oxoisovalerate dehydrogenase subunit beta | 0.6156 | 1.3 | 2 | 67 |
| IPI00002412 | PPT1 | Palmitoyl-protein thioesterase 1 | 0.6174 | 1.3 | 3 | 186 |
| IPI00024913 | PWP2 | Isoform Long of ES1 protein homolog, mitochondrial | 0.6288 | 1.2 | 10 | 508 |
| IPI00009865 | KRT10 | Keratin, type I cytoskeletal 10 | 0.6308 | 1.5 | 17 | 768 |
| IPI00477090 | - | Protein | 0.6377 | 1.3 | 7 | 364 |
| IPI00005038 | HRSP12 | Ribonuclease UK114 | 0.6396 | 1.1 | 2 | 143 |
| IPI00448095 | DCXR | L-xylulose reductase | 0.6458 | 1.2 | 6 | 244 |
| IPI00101645 | AHCYL2 | Putative adenosylhomocysteinase 3 | 0.6616 | 1.1 | 3 | 138 |
| IPI00022774 | VCP | Transitional endoplasmic reticulum ATPase | 0.6627 | 1.1 | 4 | 227 |
| IPI00011302 | CD59 | CD59 glycoprotein | 0.6784 | 1.3 | 3 | 176 |
| IPI00220906 | ACOT2 | Isoform 1 of Acyl-coenzyme A thioesterase 2 | 0.6823 | 1.1 | 2 | 58 |
| IPI00456969 | DYNC1H1 | Cytoplasmic dynein 1 heavy chain 1 | 0.6960 | 1.1 | 21 | 975 |
| IPI00306413 | TPPP3 | Tubulin polymerization-promoting protein 3 | 0.6965 | 1.0 | 2 | 76 |
| IPI00017895 | GPD2 | Isoform 1 of Glycerol-3-phosphate dehydrogenase | 0.7030 | 1.3 | 15 | 755 |
| IPI00016786 | CDC42 | Isoform 2 of Cell division control protein 42 homolog | 0.7044 | 1.1 | 3 | 132 |
| IPI00293665 | KRT6B | Keratin, type II cytoskeletal 6B | 0.7076 | 1.3 | 5 | 219 |
| IPI00412713 | SAMM50 | Sorting and assembly machinery component 50 | 0.7081 | 1.1 | 2 | 189 |
| IPI00015476 | SLC1A4 | Neutral amino acid transporter A | 0.7131 | 1.1 | 2 | 76 |
| IPI00220739 | PGRMC1 | Membrane-assoc progesterone receptor component 1 | 0.721 | 1.2 | 3 | 191 |
| IPI00020530 | ACOT13 | Acyl-coenzyme A thioesterase 13 | 0.7212 | 1.1 | 3 | 218 |
| IPI00033025 | SEPT7 | 51 kDa protein, septin 7 | 0.7253 | 1.1 | 17 | 1041 |
| IPI00220710 | ACOT9 | Isoform 1 of Acyl-coenzyme A thioesterase 9 | 0.7311 | 1.1 | 3 | 94 |
| IPI00027252 | PHB2 | Prohibitin-2 | 0.7321 | 1.1 | 16 | 923 |
| IPI00658113 | SEPT8 | Putative uncharacterized protein SEPT8 | 0.7325 | 1.2 | 12 | 536 |
| IPI00296333 | ACSL6 | Isoform 3 of Long-chain-fatty-acid-CoA ligase 6 | 0.7351 | 1.0 | 5 | 162 |
| IPI00024976 | TOMM22 | Mitochondrial import receptor subunit TOM22 | 0.7375 | 1.2 | 2 | 123 |
| IPI00003479 | MAPK1 | Mitogen-activated protein kinase 1 | 0.7448 | 1.0 | 8 | 319 |
| IPI00478493 | HP | Haptoglobin isoform 2 preproprotein | 0.7472 | 1.1 | 2 | 130 |
| IPI00748145 | GNAI2 | Isoform 1 Guanine nucleotide-binding protein G(i), α-2 | 0.7625 | 1.1 | 15 | 1000 |
| IPI00003944 | DBT | Lipoamide acyltransferase, α-keto acid dehydrogenase | 0.7635 | 1.1 | 2 | 152 |
| IPI00220327 | KRT1 | Keratin, type II cytoskeletal 1 | 0.7698 | 1.0 | 14 | 882 |
| IPI00008868 | MAP1B | Microtubule-associated protein 1B | 0.7714 | 1.2 | 30 | 1444 |
| IPI00023001 | FAM162A | UPF0389 protein FAM162A | 0.7754 | 1.0 | 6 | 226 |
| IPI00182655 | KRT81 | Keratin, type II cuticular Hb6 | 0.7907 | 2.3 | 2 | 81 |
| IPI00004358 | PYGB | Glycogen phosphorylase, brain form | 0.7939 | 1.0 | 5 | 181 |
| IPI00017334 | PHB | Prohibitin | 0.7945 | 1.1 | 14 | 904 |
| IPI00027438 | FLOT1 | Flotillin-1 | 0.8030 | 1.1 | 2 | 127 |
| IPI00215919 | ARF5 | ADP-ribosylation factor 5 | 0.8043 | 1.1 | 3 | 128 |
| IPI00017283 | IARS2 | Isoleucyl-tRNA synthetase, mitochondrial | 0.8092 | 1.1 | 3 | 212 |
| IPI00215777 | SLC25A3 | Isoform B of Phosphate carrier protein | 0.8120 | 1.0 | 18 | 869 |
| IPI00418497 | TIMM50 | Mitochondrial inner membrane translocase TIM50 | 0.8221 | 1.2 | 3 | 127 |
| IPI00007765 | HSPA9 | Stress-70 protein | 0.8251 | 1.0 | 29 | 1873 |
| IPI00151462 | MAP1lc3B2 | Microtubule-assoc proteins 1A/1B light chain 3 β2 | 0.8283 | 1.1 | 2 | 85 |
| IPI00006662 | APOD | Apolipoprotein D | 0.8291 | 1.3 | 3 | 124 |
| IPI00015833 | CHCHD3 | Coiled-helix-coil-helix domain-containing protein 3 | 0.8368 | 1.1 | 5 | 324 |
| IPI00296365 | CENPE | Isoform 1 of Centromere-associated protein E | 0.8409 | 1.1 | 2 | 57 |
| IPI00009532 | ABAT | highly similar to 4-aminobutyrate aminotransferase | 0.8454 | 1.1 | 25 | 1845 |
| IPI00014232 | ARL6IP1 | ADP-ribosylation like protein 6-interacting protein 1 | 0.8502 | 1.8 | 2 | 50 |
| IPI00001453 | INA | Alpha-internexin | 0.8502 | 1.1 | 20 | 1123 |
| IPI00848328 | - | Delta(3,5)-Delta(2,4)-dienoyl-CoA isomerase | 0.8604 | 1.5 | 2 | 120 |
| IPI00216932 | ACSS1 | Isoform 1 of Acetyl-coenzyme A synthetase 2-like | 0.8675 | 2.6 | 3 | 117 |
| IPI00003833 | MTCH2 | Mitochondrial carrier homolog 2 | 0.8898 | 1.0 | 10 | 619 |
| IPI00022082 | SEPT8 | Isoform 2 of Septin-8 | 0.8918 | 1.1 | 12 | 536 |
| IPI00027834 | HNRNPL | Heterogeneous nuclear ribonucleoprotein L | 0.8939 | 1.1 | 2 | 154 |
| IPI00024990 | ALDH6A1 | Methylmalonate-semialdehyde dehydrogenase | 0.8977 | 1.2 | 19 | 986 |
| IPI00411706 | ESD | S-formylglutathione hydrolase | 0.9098 | 1.0 | 2 | 89 |
| IPI00410079 | FAM82A2 | Isoform 1 Regulator of microtubule dynamics protein 3 | 0.9269 | 1.1 | 4 | 301 |
| IPI00018195 | MAPK3 | Mitogen-activated protein kinase 3 | 0.9324 | 1.0 | 3 | 164 |
| IPI00237671 | NEFL | Neurofilament light polypeptide | 0.9334 | 1.1 | 23 | 1198 |
| IPI00017592 | LETM1 | Isoform 1 LETM1 & EF-hand domain-contain 1 | 0.9443 | 1.1 | 6 | 413 |
| IPI00029162 | CDC2L5 | Isoform 2 of Cell division cycle 2-like protein kinase 5 | 0.9505 | 1.0 | 3 | 73 |
| IPI00004845 | NIPSNAP3A | Protein NipSnap homolog 3A | 0.9519 | 1.1 | 4 | 240 |
| IPI00383798 | PRODH | Isoform 4 of Proline dehydrogenase | 0.9609 | 1.9 | 2 | 145 |
| IPI00339269 | HSPA6 | Heat shock 70 kDa protein 6 | 0.9644 | 1.0 | 6 | 313 |
| IPI00431405 | C5orf33 | Isoform 2 of UPF0465 protein C5orf33 | 0.9671 | 1.1 | 2 | 132 |
| IPI00554701 | UCRC | Cytochrome b-c1 complex subunit 9 | 0.9693 | 1.2 | 2 | 129 |
| IPI00220827 | TMSB10 | Thymosin beta-10 | 0.9845 | 1.3 | 3 | 154 |
| IPI00550021 | RPL3 | 60S ribosomal protein L3 | 0.9900 | 1.0 | 2 | 62 |
| IPI00018342 | AK1 | Adenylate kinase isoenzyme 1 | 0.9914 | 1.1 | 2 | 118 |
| IPI00003031 | ISOC2 | Isoform 2 Isochorismatase domain-containing protein 2 | 0.9916 | 1.1 | 5 | 411 |
| IPI00100656 | TECR | Isoform 1 of Synaptic glycoprotein SC2 | 0.9970 | 1.0 | 3 | 112 |

1. P-value evaluated by one-way ANOVA on intensity data (computed using Progenesis); (b) Ratio of the average protein intensity in GBM and peritumoural control samples, measured by Progenesis; (c) Number of peptides used for quantitation; (d) Protein identification score (calculated by Mascot)
