# Supplementary figures and images for: Interactions among mitochondrial proteins altered in glioblastoma
Source: J Neurooncol. 2014 Apr 13;118(2):247–56. doi: 10.1007/s11060-014-1430-5 (PMC4048470; doi:10.1007/s11060-014-1430-5)

## Slide 1
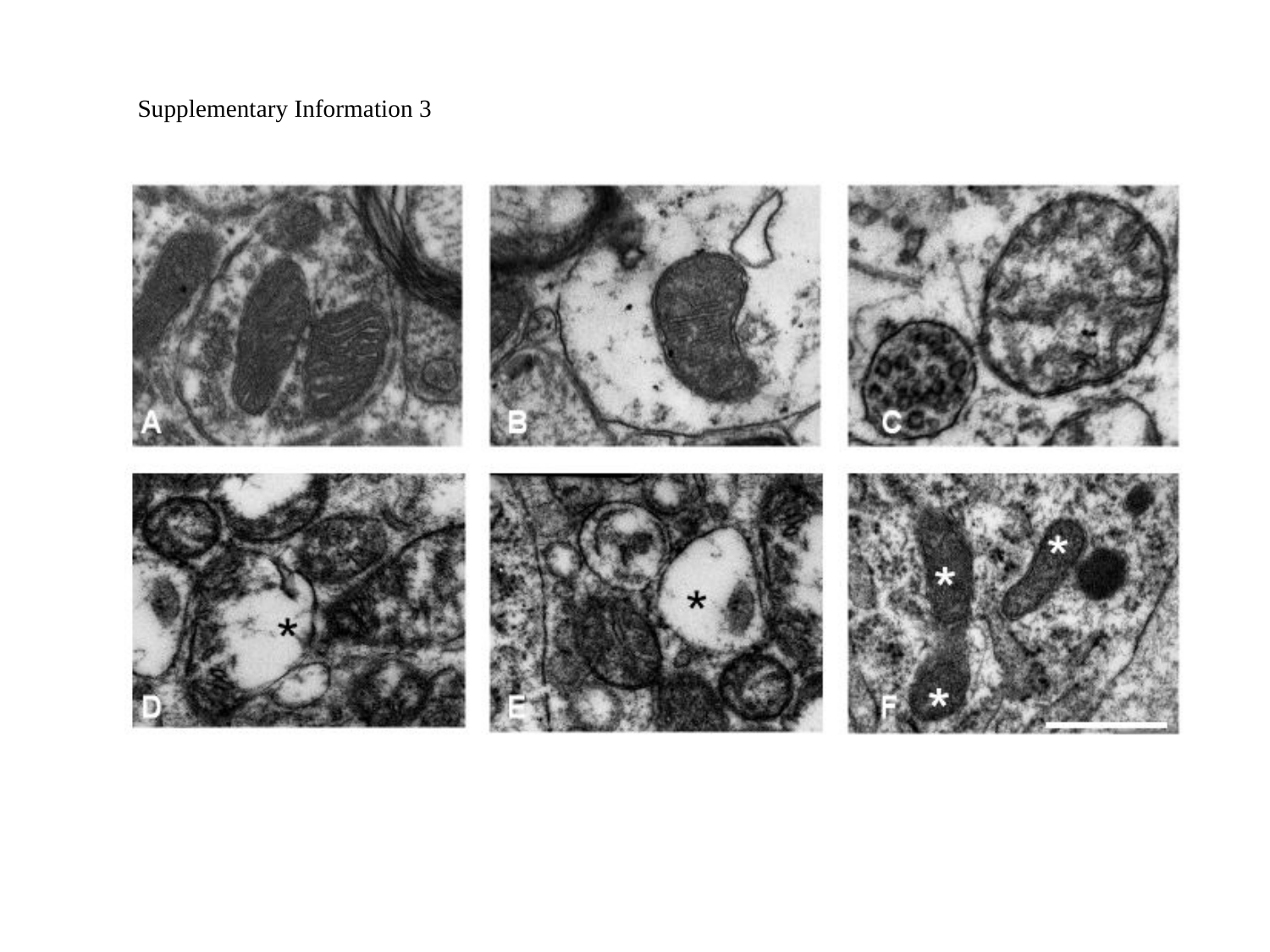

Supplementary Information 3

Supplement: Supplementary file 3 — S3: Morphological Classification of Mitochondria. The morphology of mitochondria in GBM (relative to control brain) was assessed using Electron Microscopy and 6 a priori categories of morphology represented by images A – F. The asterisks indicate the mitochondria that represent the category in each image where there is more than one mitochondrion present. Image [A] represents ‘normal’ mitochondria, where cristae are visible throughout the mitochondria or in at least 50 % (as judged qualitatively) of the mitochondria interior area. Image [B] shows a mitochondrion that is predominantly normal but shows few cristae occupying less than 50 % of the interior area. Images [C, D, E,] depict ‘abnormal’ mitochondria with very few cristae, interior matrix condensed and dark or round swollen with interior missing. The images show a progression of morphological disruption with image [C] showing mitochondria that are vesicular, [D] showing mitochondria that are part swollen and part vesicular, and [E] showing mitochondria that are significantly swollen. Image [F] shows mitochondria that the evaluator could not classify due to undetermined abnormality. These might be arising from artefacts i.e. cutting, fixation or obstruction. Images [B & F] represent mitochondria that could not be straightforwardly classified as ‘normal’ or ‘abnormal’. The scale bar represents 0.5 μm. Supplementary material 3 (PPT 294 kb) [file 11060_2014_1430_MOESM3_ESM.ppt]
