# Supplementary material for: Interactions among mitochondrial proteins altered in glioblastoma
Source: J Neurooncol. 2014 Apr 13;118(2):247–56. doi: 10.1007/s11060-014-1430-5 (PMC4048470; doi:10.1007/s11060-014-1430-5)
Supplement: Supplementary file 4 — S4: Integrity of enriched mitochondrial fractions. Western blot analysis of 3 mitochondrial membrane proteins (VDAC1 localised to the outer mitochondrial membrane; COXI and COXIV localised to the inner mitochondrial membrane) in the supernatant (s) and mitochondrial (m) fractions generated for LC–MS (n = 6 pertitumoural control; n = 6 GBM; see methods section on mitochondrial fractionation). All 3 proteins are present in the mitochondrial fractions (m) but absent in the supernatant fractions (s) in both GBM and peritumoural control. There is no evidence that there are differential effects of fractionation in GBM and peritumoural control brain tissue. Supplementary material 4 (PPT 174 kb) [file 11060_2014_1430_MOESM4_ESM.ppt]

## Slide 1
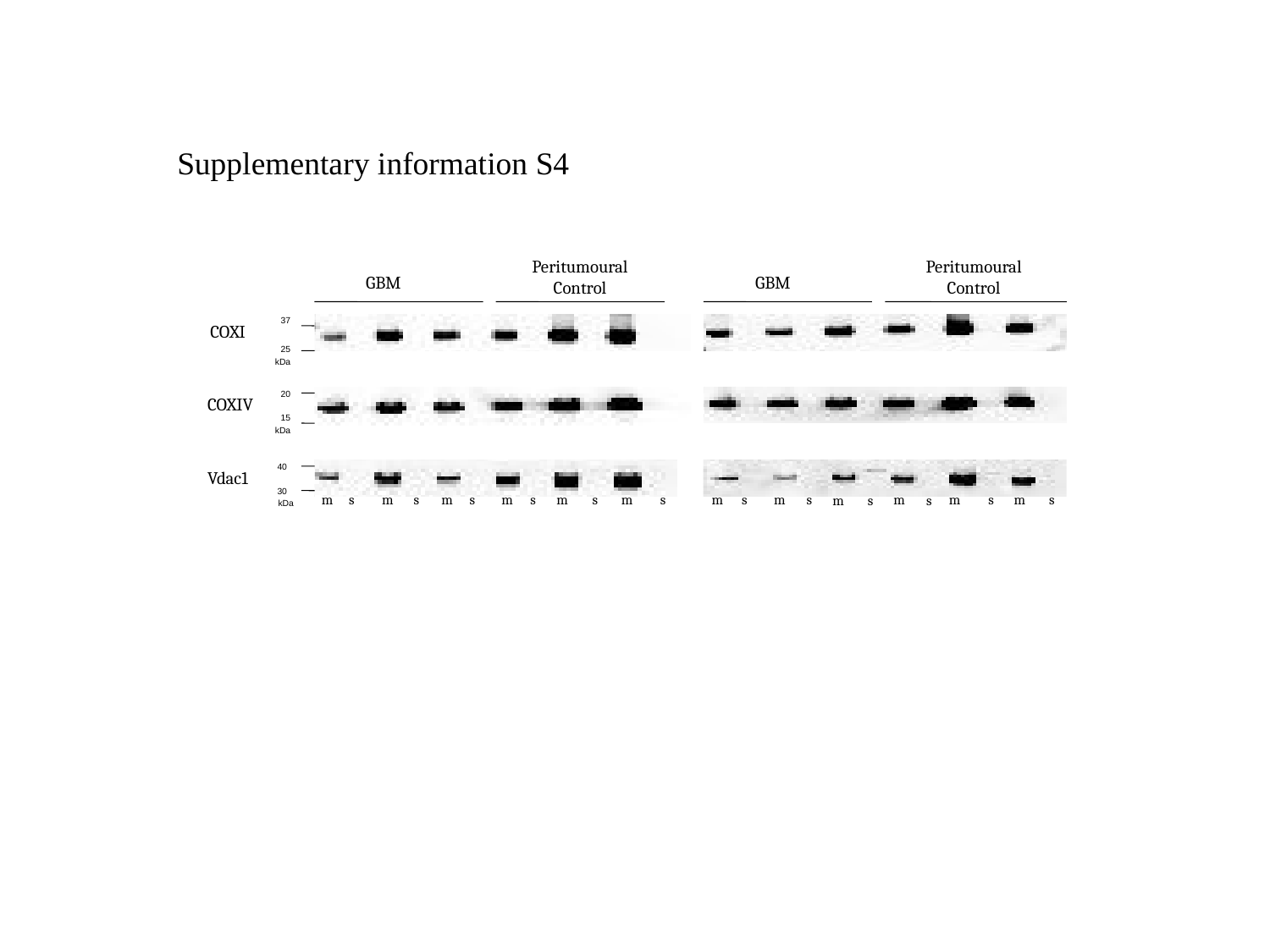

Supplementary information S4
PeritumouralControl
Peritumoural Control
GBM
GBM
37
COXI
25
kDa
20
COXIV
15
kDa
40
Vdac1
30
m
s
m
s
m
s
m
s
m
s
m
s
m
s
m
s
m
m
s
m
s
m
s
s
kDa
