# Supplementary material for: Interactions among mitochondrial proteins altered in glioblastoma
Source: J Neurooncol. 2014 Apr 13;118(2):247–56. doi: 10.1007/s11060-014-1430-5 (PMC4048470; doi:10.1007/s11060-014-1430-5)
Supplement: Supplementary file 5 — S5: Summary of LC–MS identifications from GBM and peritumoural control brain mitochondrial fractions. Total number of proteins identified (with ≥2 peptides) in the mitochondrial enriched fractions extracted from GBM and peritumoural control brain; total number of proteins recognised by DAVID software; total number of proteins identified as being mitochondrially-associated based on the GO designation mitochondrion; and total number of dysregulated mitochondrial proteins (p ≤ 0.05, ≥2-fold change) in GBM compared to peritumoural control brain. Supplementary material 5 (DOC 27 kb) [file 11060_2014_1430_MOESM5_ESM.doc]

Supplementary Information 5

| **Breakdown of protein identifications and protein level alterations** | **#** |
| --- | --- |
| Proteins identified ≥2 peptides | 902 |
| Proteins (≥2 peptides) matched to DAVID identifications | 833 |
| Mitochondrial proteins (≥2 peptides) – GO Term 0005739 mitochondrion | 256 |
| Total mitochondrial proteins regulated in expression level (up/down; p≤0.05) | 117 |
| *- upregulated mitochondrial proteins (p≤0.05)* | *39* |
| *- downregulated mitochondrial proteins (p≤0.05)* | *78* |
