# Supplementary material for: Interactions among mitochondrial proteins altered in glioblastoma
Source: J Neurooncol. 2014 Apr 13;118(2):247–56. doi: 10.1007/s11060-014-1430-5 (PMC4048470; doi:10.1007/s11060-014-1430-5)
Supplement: Supplementary file 6 — S6: Putative interactions between mitochondrial proteins altered in GBM. Protein–protein interaction networks (‘interactomes’) generated by Ingenuity Pathway Analysis (http://www.ingenuity.com). The proteins highlighted in bold are the mitochondrial proteins found significantly altered in the study (t-test p ≤ 0.05, ≥2-fold change) in GBM, and are termed ‘Focus Molecules’. Proteins not in bold have been inserted by IPA and are proteins (not exclusive to mitochondria) that interact with the focus molecules. The coloured arrows indicate the direction of response of the focus molecule in GBM (red = increased; green = decreased). Each network is assigned a score by IPA. Network scores are putatively a measure of probability for the network (but see Deighton et al. [16], for critical analysis of this issue). Supplementary material 6 (DOC 47 kb) [file 11060_2014_1430_MOESM6_ESM.doc]

**Supplementary Information 6**

| **Network ID** | **Molecules in MITOCHONDRIA GBM Networks** | **Score** | **Focus Molecules** |
| --- | --- | --- | --- |
| 1. ET chain  (complex I) | **↑GLDC**, MT-ND3, MT-ND5, MT-ND4L, NADH dehydrogenase, NADH2 dehydrogenase, NADH2 dehydrogenase (ubiquinone), **↓NDUFA2**, **↓NDUFA3**, **↓NDUFA4**, NDUFA6, **↓NDUFA7**, **↓NDUFA9**, **↓NDUFA10**, **↓NDUFA11**, **↓NDUFA12**, NDUFAF1, **↓NDUFB3**, **↓NDUFB5**, NDUFB6, **↓NDUFB8**, **↓NDUFB10**, **↓NDUFC2**, **↓NDUFS1**, **↓NDUFS2**, **↓NDUFS3**, **↓NDUFS4**, **↓NDUFS5**, **↓NDUFS6**, **↓NDUFS7**, **↓NDUFS8**, **↓NDUFV1**, **↓NDUFV2**, **↑TST** | 52 | 25 |
| 2. ET chain  (synuclein interaction) | **↑ACADM**, APP, **↑CAT**, **↓COX6B1**, **↓COX6C**, **↓DLAT**, DLD, **↓DNAJC5**, DNAJC11, ETFB, GRK1, **↓HK1**, **↑HSD17B10**, HTT, IAPP, KLF11, MAPK3, **↓MTX2**, **↓NDUFA9**, **↓NDUFA11**, **↓NDUFA12**, **↓NDUFB8**, **↓NDUFB10**, **↓NDUFS4**, **↓NDUFS7**, **↓NDUFV1**, **↑PC**, Pdha1, **↓PDHB**, **↓SLC25A11**, **↓SLC25A12**, **↓SLC25A22**, SNCA, **↓SNCB**, **↑TPP1** | 46 | 24 |
| 3. ET chain (complex IV) | **↑BSG**, COX17, **↓COX4I1**, **↓COX5A**, **↓COX5B**, **↓COX6B1**, **↓COX6C**, **↓COX7A2**, **↓COX7A2L**, COX7C, Cytochrome C, Cytochrome C oxidase, **↑DECR1**, **↑ECI1**, EHHADH, **↑GPX1**, **↓IDH3A**, **↓IDH3B**, **↓IDH3G**, isocitrate dehydrogenase (NAD), malate dehydrogenase (oxaloacetate-decarboxylating) (NADH), ME1, **↑ME2**, **↓ME3**, MT-CO2, MT-CO3, MT-COI, nitric oxide, **↓OGDHL**, **↓OXCT1**, PPARGC1A, **↑SCP2**, SLC27A1 | 37 | 19 |
| 4. HNF4A | **↑AASS**, **↑ACAA2**, **↓ACO2**, **↑ACSS3**, **↑AK2**, **BDH1**, CDCA7L, **↑CHDH**, DHRS2, **↑FDXR**, FHIT, HNF4A, LOC10431/TIMM23, **↑MAOA**, **↑MCCC1**, **↑MGST1**, **↑MTHFD1**, PKM2, Pld, PLD2, SERINC3, **↓STXBP1**, SYTL4, TIMM9, TIMM10, TIMM17A, TIMM17B, TIMM8B, Tomm5, TOMM6, TOMM7, Tomm20l, TOMM40L, **↓VDAC2**, **↓VDAC3** | 27 | 15 |
| 5. Energy production (complex V) | Adenosine-tetraphosphatase, ALAD, ATP synthase, **↓ATP5A1**, **↓ATP5B**, ATP5C1, **↓ATP5D**, ATP5E, ATP5F1, **↓ATP5H**, ATP5I, **↓ATP5J**, ATP5J2, ATP5L, **↓ATP5O**, ATP5S, ATP6V0E1, **↓ATP6V1A**, **↓ATP6V1E1**, ATPV1G1, ATPV1G2, ATP6V1H, ATPAF1, **↓GLRX5**, H+-transporting two sector ATPase, Naca, **↑NEFH**, **↑PRDX1**, **↑PRDX4**, SRXN1, **↓UQCRFS1**, VHL | 23 | 15 |
| 6. MYC + Creatine Kinase | ANP32B, **↓ATPIF1**, CKB, **↓CKMT2**, **↓CKMT1A/CKMT1B**, **↑CLIC4**, **↑CLU**, Creatine Kinase, CTNNA1, CTNNB1, **↑CTSB**, DDB2, DMWD, FOXO3, **↓GLS**, HSP90B1, HSPD1, IQGAP2, KLF4, **↑LAP3**, LRP2, MT3, MYC, **↓NIPSNAP1**, REN, **↑SLC25A1**, SMARCA5, **↑SOD2**, **↓SUCLA2**, **↑SUCLG2**, TFAP2A, USP19, VCL | 22 | 13 |
| 7. Metabolism + Ion Transport | AAK1, ABL2, ACAP1, **↓ACOT7**, alpha actinin, AP1G2, **↓AP2M1**, AP2S1, AP3B2, **↓ATP5O**, CACNA1B, CD22, **↓CISD1**, **↓CLTC**, **↑CLU**, DLG4, **↑DPYSL2**, EPN2, **↓GOT2**, **↑GPX4**, GRB2, **↑HIBCH**, LRP8, LY9, **↑MAOB**, NCKIPSD, OCRL, OTUD5, PICALM, PLD2, PRKAB1, RTKN2, SCYL2, **↑SHMT2**, TGOLN2 | 20 | 12 |
| 8. | **↑GATM**, glycine amidinotransferase | 2 | 1 |
| 9. | **↑ABCB7**, FECH | 2 | 1 |
| 10 | **↓AUH**, enoyl-CoA hydratase, methylglutaconyl-CoA hydratase | 2 | 1 |
| 11 | KLF5, **↓PCCA**, PCCB, propionyl-CoA carboxylase | 2 | 1 |
