# Supplementary material for: Interactions among mitochondrial proteins altered in glioblastoma
Source: J Neurooncol. 2014 Apr 13;118(2):247–56. doi: 10.1007/s11060-014-1430-5 (PMC4048470; doi:10.1007/s11060-014-1430-5)
Supplement: Supplementary file 7 — S7: Mitochondrial Interactomes in GBM. Visual representations of putative protein–protein interactions in the high scoring networks (‘ETC Networks 1-3 and 5’ (A-D); ‘MYC + Creatine Kinase’ network (E); and ‘Ion Transport’ network (F); as listed in S4) generated by IPA from mitochondrial proteins altered in GBM. In the network, each node (shape) represents a protein and its association with other proteins is represented by a line. Nodes have different shapes that represent different molecule types, for example transcription factors, enzymes, kinases and phosphatases (refer to Ingenuity Systems Software for detailed node information). Mitochondrial proteins or ‘nodes’ with a coloured background were regulated in the study (green = decreased; red = increased) whilst other interacting proteins with no background are proteins not detected in this study that have been inserted by IPA (and are not exclusive to mitochondria) to produce a highly connected network. The solid lines represent direct interactions or associations between proteins. Supplementary material 7 (PPT 819 kb) [file 11060_2014_1430_MOESM7_ESM.ppt]

## Slide 1
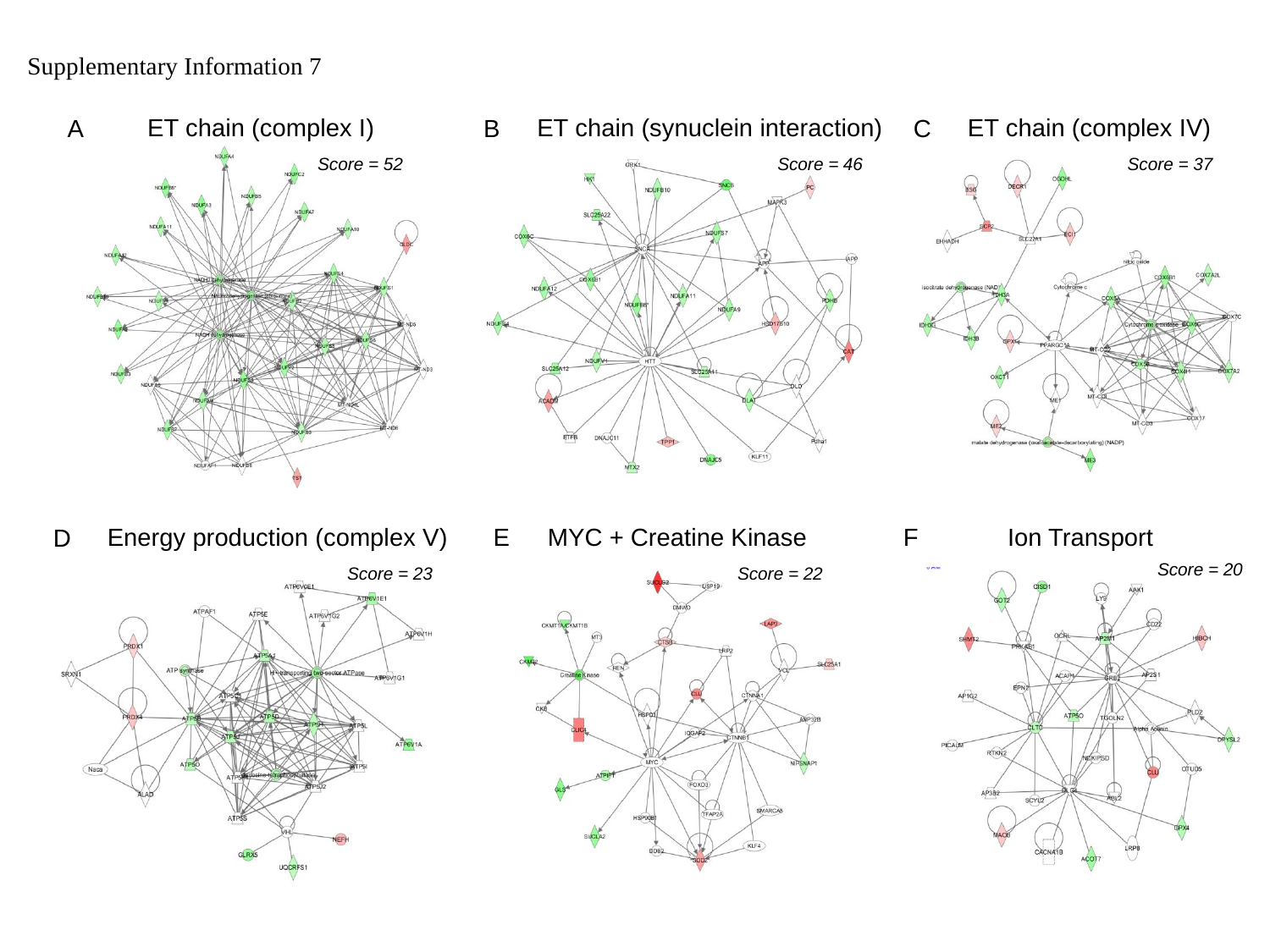

Supplementary Information 7
ET chain (complex I)
ET chain (synuclein interaction)
ET chain (complex IV)
A
B
C
Score = 52
Score = 46
Score = 37
Energy production (complex V)
E
MYC + Creatine Kinase
F
Ion Transport
D
Score = 20
Score = 23
Score = 22
